# Substrate specific closed-loop optimization of carbohydrate protective group chemistry using Bayesian optimization and transfer learning†

**DOI:** 10.1039/d3sc01261a

**Published:** 2023-05-18

**Authors:** Natasha Videcrantz Faurschou, Rolf Hejle Taaning, Christian Marcus Pedersen

**Affiliations:** a Department of Chemistry, University of Copenhagen Universitetsparken 5 2100 Copenhagen Ø Denmark cmp@chem.ku.dk; b LEO Pharma A/S Industriparken 55 2750 Ballerup Denmark

## Abstract

A new way of performing reaction optimization within carbohydrate chemistry is presented. This is done by performing closed-loop optimization of regioselective benzoylation of unprotected glycosides using Bayesian optimization. Both 6-*O*-monobenzoylations and 3,6-*O*-dibenzoylations of three different monosaccharides are optimized. A novel transfer learning approach, where data from previous optimizations of different substrates is used to speed up the optimizations, has also been developed. The optimal conditions found by the Bayesian optimization algorithm provide new insight into substrate specificity, as the conditions found are significantly different. In most cases, the optimal conditions include Et_3_N and benzoic anhydride, a new reagent combination for these reactions, discovered by the algorithm, demonstrating the power of this concept to widen the chemical space. Further, the developed procedures include ambient conditions and short reaction times.

## Abstract 2

Closed-loop optimizations of regioselective benzoylations of unprotected carbohydrates using automated synthesis and Bayesian optimization. The autonomous search revealed novel procedures for both regioselective mono and dibenzoylations.

## Graphical abstract

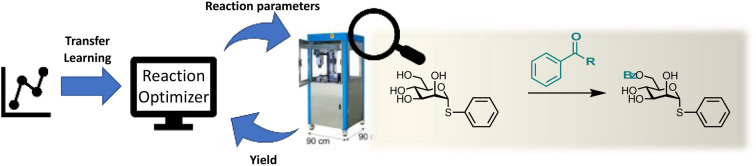

## Introduction

In recent years Bayesian optimization has gained growing interest as a tool for reaction optimization,^1–7^ and in some cases, it has been shown to be capable of outcompeting chemist intuition.^1,8^ Further, Bayesian optimization can be combined with automated setups to obtain closed-loop systems.^2–7^ Most of these closed-loop systems rely on a setup with a flow cell^2–4,9^ and, recently, even closed-loop optimization of a multi-step synthesis using a flow reactor setup and Bayesian optimization has been described.^5^ However, the flow setups are often limited to fast and homogeneous reactions.^10^ The Hein group has, by utilizing a robotic platform, shown that closed-loop optimization of batch synthesis is also possible.^6^ Investigating optimization of batch synthesis is of high interest, as it is a widespread approach throughout academia and industry, and it can easily be used directly in research and process optimization. Our focus is to combine Bayesian optimization with carbohydrate chemistry, since optimizing reactions with carbohydrates is notoriously tedious and labor-intensive. Hence, “optimizing” optimization of reactions involving carbohydrates is of interest. There are two major fields within oligosaccharide synthesis: glycosylation reactions and protective group chemistry. One of the major challenges in glycosylation reactions is controlling the anomeric diastereoselectivity, as the reaction often proceeds with a high degree of cationic character. A specific configuration of the anomeric center is however crucial for structural and hence biological properties and therefore this is a very active field of research.^14–18^ Some guidelines for controlling the diastereoselectivity have been established, for instance, the use of “solvent effects” to favor different anomeric configurations,^19,20^ though, these are ambiguous and might lead to advance assumptions, which could cause the chemist to not investigate certain conditions, that in fact might be the optimal ones. Protective groups can be used to guide the glycosylations toward a certain anomeric selectivity. Protecting carbohydrates is complicated by the presence of numerous hydroxyl groups, and regioselective procedures are necessary. The inherent complexity, exemplified above, makes it difficult to predict both the regioselectivity and stereoselectivity in reactions on carbohydrates. Reactions involving carbohydrates are further complicated by substrate specificity. Many elegant methods for obtaining regioselectivity and stereoselectivity have been established.^15,21,22^ However, the regioselectivity/stereoselectivity is often a consequence of specific interactions with a promoter or catalyst, and the success of the methods is therefore very sensitive to changes in the carbohydrate substrate, which often deteriorates the selectivity or significantly decreases the yield. We want to investigate if Bayesian optimization can help overcome some of these issues. In this paper, we explore how closed-loop optimization and Bayesian optimization can be used to optimize carbohydrate protective group chemistry, specifically regioselective benzoylations of unprotected glycosides.

**Scheme 1:**
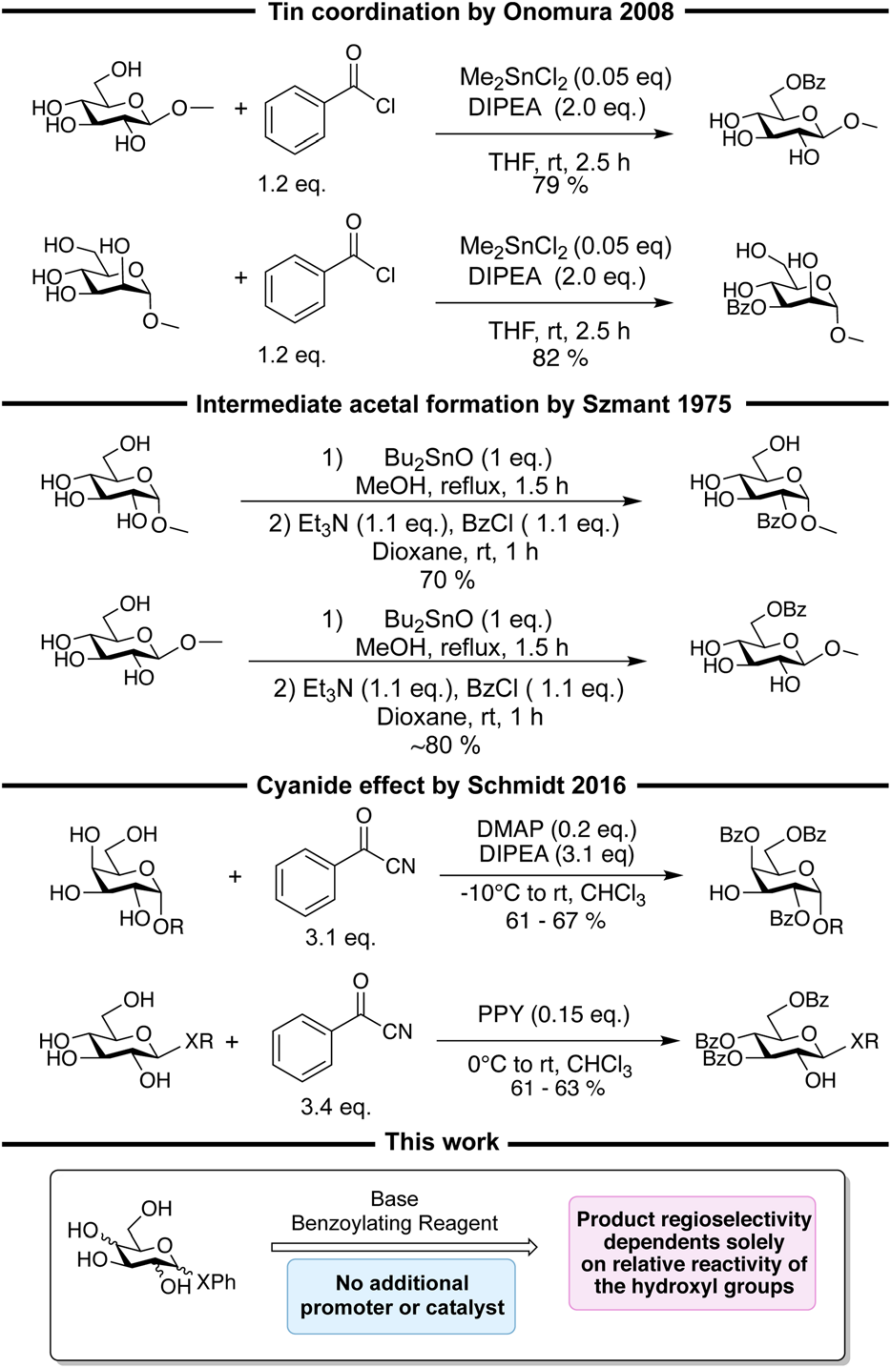
Examples of regioselective benzoylations with regioselectivity arising from coordination,^11^ stannylene acetal formation,^12^ and the cyanide effect,^13^ followed by an illustration of the procedure described in this work, which only utilize base and benzoylating reagent, and no other additive.

**Fig. 1:**
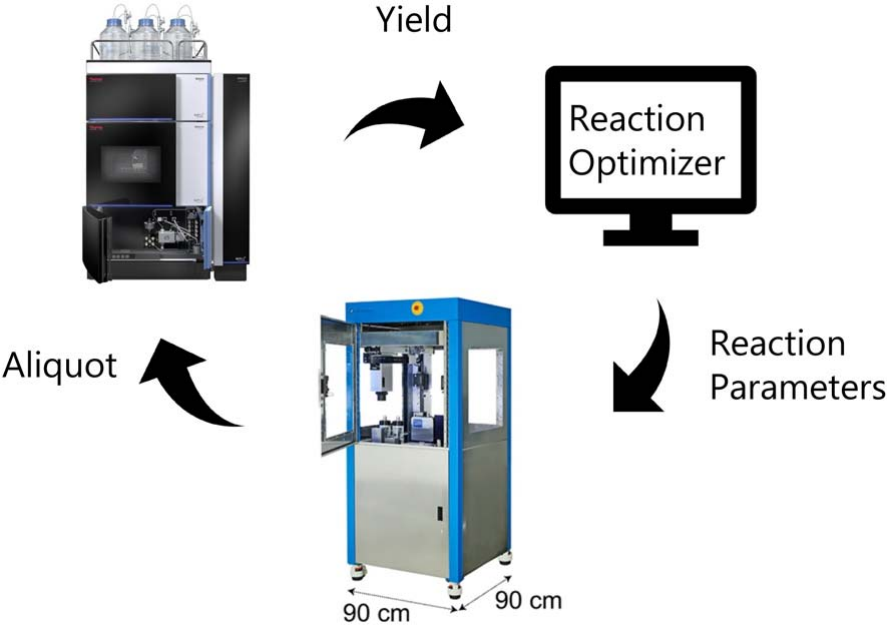
Illustration of closed-loop setup. The reaction optimizer proposes an experiment that is carried out by the robot and then analyzed by UPLC.

Different procedures for regioselective benzoylations have been described, though most rely on either coordination to a catalyst/promoter^11,23–26^ or regioselective formation of an acetal intermediate.^12,27,28^ Non-trivial benzoylating reagents like benzoyl cyanide^13,29,30^ and *N*-benzoylimidazole^31^ have also been utilized to obtain regioselective in these reactions. In general, most of these procedures show high substrate specificity with the regioselectivity determined by the configuration of the hydroxyl groups relative to each other. The regioselectivity can for instance be determined by a preference for *cis*-vicinal diols,^16,32–34^ a preference for diequatorial vicinal diols,^25^ the axial oxy effect^30^ the cyanide effect,^13,29^ or dual hydrogen bonding.^35,36^ Another aspect of the current methods is the necessity for more steps, complexations, and long reaction times at low temperatures in order to obtain acceptable selectivities. Some of the above-mentioned approaches are illustrated in Scheme 1. From the reactions in Scheme 1, it is seen that changing the carbohydrate substrate can change the selectivity, which exemplifies the substrate specificity. In theory, it should be possible to develop regioselective benzoylation procedures that solely rely on the inherent differences in reactivity of the hydroxyl groups and some procedures using this approach for obtaining tribenzoylated glycosides in moderate yields (37–65%) have been described.^37,38^ Though, the order of reactivity of the hydroxyl groups depends on the carbohydrate,^37,39^ and further, different studies of the reactivity of the hydroxyl groups observe different orders of reactivity, making the development of procedures solely relying on reactivity a tedious and non-trivial task (Fig. 1).

The lack of obvious optimal reaction conditions makes regioselective benzoylations an appropriate and challenging test case for investigating if, and how, Bayesian optimization can aid in developing new methods for protecting complex natural products like carbohydrates. Furthermore, for a method relying on reactivity rather than coordination *etc.* changing the carbohydrate substrate should not change the regioselectivity, unless changing the configuration of the carbohydrate leads to a change in relative reactivity. Although, changing the carbohydrate substrate might still lead to a change in the optimal reaction conditions. We, therefore, decided to include substrate specificity in our study, to both investigate how great an impact the substrate has on the optimal conditions, and also to investigate how substrate specificity can be taken into account in the most efficient way.

## Results and discussion

### Design of closed-loop setup

**Fig. 2:**
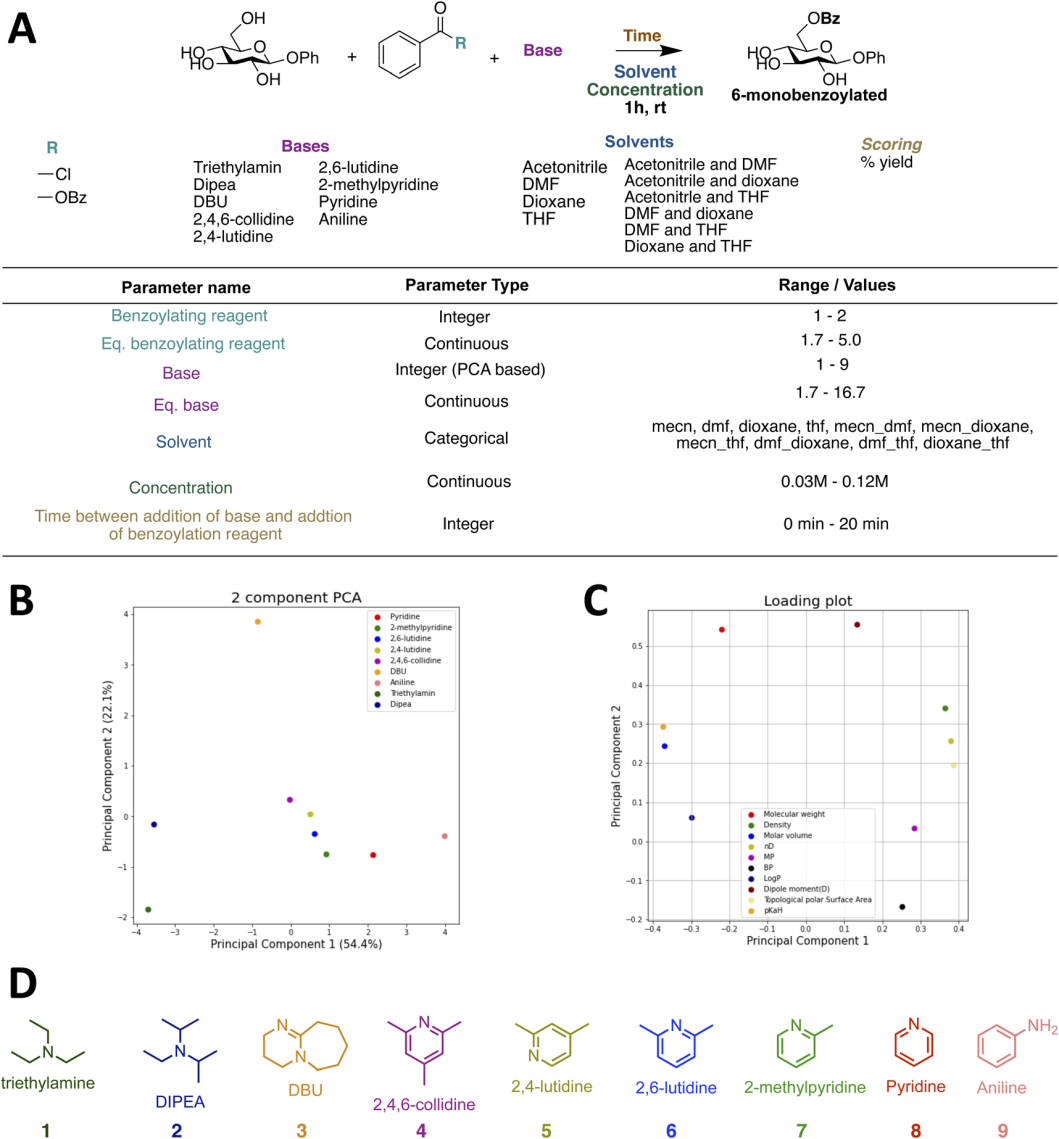
(A) Reaction and reaction space for initial closed-loop optimization (CL1). (B) First two calculated principal components for the 9 bases. (C) Loading plot for the descriptors used to calculate the principal components. (D): Integers assigned to the bases using the first principal component.

To perform the closed-loop optimizations the setup shown in Fig. 2 was utilized. The setup consists of a computer with a Bayesian optimization algorithm (in the following referred to as the ReactionOptimizer), a ChemSpeed Flex robotic synthesis platform, and a UPLC. An introduction to theory behind Bayesian optimization is supplied in the ESI.† The robot is capable of fetching experiments proposed by the ReactionOptimizer through an SQL database. When an experiment has been carried out by the synthesis robot, an aliquot is transferred to the UPLC and the area under the peak from the desired compound is sent to the ReactionOptimizer. Using a predefined scoring function, the ReactionOptimizer calculates a result using the area under the peak. From the result of the performed experiment and the results from all previous experiments, the ReactionOptimizer suggests a new experiment to be carried out by the robot. The Bayesian optimization algorithm used by the ReactionOptimizer is freely available on GitHub.^40^ To get an understanding of the different hyperparameters of the optimization algorithm a benchmark study was carried out. The benchmark study can be found in ESI.† An important hyperparameter is the xi-value, which describes how explorative the optimization algorithm is (see theory section in ESI† for details). To avoid the trade-off between exploration and exploitation we chose a dual approach where the algorithm starts by using a high xi-value, and after a predefined number of experiments switch to a low xi-value. The idea behind this approach is that the algorithm starts by being explorative and traversing many different areas of the high-dimensional surface, while still optimizing. Using the obtained information about the high-dimensional surface the algorithm carefully approaches the optimum when switching to the lower xi-value. The xi-changeover is predefined and determined before initializing the ReactionOptimizer. The changeover value was determined using the benchmark study and previous closed-loop optimization. Instead of a trade-off between exploration and exploitation, the dual approach leads to a trade-off between time and improvement. Ideally, the xi-value should be switched from high to low when we are confident the global maximum is found, and after the switch, this maximum is explored using a lower xi-value. However, time, *i.e.*, budget, also has to be considered, and the xi-changeover has also been determined with an aim to have the total time of the optimization within a reasonable timeframe, and the total time of the optimization is defined by the total number of experiments. Closed-loops, where initial random experiments are included in the optimization, have been proposed using Latin hypercube sampling (LHS).^41^

### Design of model reaction and reaction space

To test the setup, it was desirable to have a model reaction that is a real optimization problem, where the yield is zero in some of the reaction space and non-zero in other parts. The chosen model reaction is the regioselective benzoylation of the 6-OH of a β-glucoside (Fig. 2). The reaction time and temperature are set as constants, with a value of 1 hour and room temperature, respectively. This is done to both speed up the optimization, but also to make the final procedure fast and simple, so that the procedure will be useful for carbohydrate chemists if a high-yielding procedure is found. Following the same reasoning, the reaction space is kept simple and only includes cheap and easily accessible reagents (Fig. 2A). In total 7 parameters are optimized simultaneously during the closed-loop, including equivalent benzoylating reagent, equivalent base, and concentration as continuous parameters. Discrete parameters are described as integers and include benzoylating reagent, base, and time between the addition of base and the addition of benzoylating reagent. Benzoyl chloride and benzoic anhydride were assigned the integers 1 and 2, respectively.

However, for the base parameter, more than two bases were included in the reaction space, and a principal component analysis (PCA) was therefore performed to assign integers in a meaningful way, which we hypothesize would speed up the optimization, compared to assigning random integers. The PCA was performed using the descriptors shown with their origin in Table 1. The first plot in Fig. 2B shows the two first principal components. The loading plot shown in Fig. 2C illustrates how the different descriptors contribute to the principal components. It is seen that all descriptors contribute to the first principal component, as they are all placed on either side of the vertical line that passes through (0,0), whereas for the second principal component the major contributions are molecular weight and dipole moment. Only the first is used for the integer assignment in Fig. 2D, it is seen from the ordering that PCA gives an order which resonates well with chemical intuition. Triethylamine and DIPEA are assigned 1 and 2, respectively, and the pyridine-based bases are assigned (4–8) and according to steric hindrance. However, PCA also allows us to place bases with non-obvious positions, like DBU and aniline, on this spectrum. Since we wanted to include solvent mixtures, it was not possible to use a similar PCA approach to describe the solvents and they were instead described as a categorical variable. Four different solvents and all possible 1 : 1 combinations are included in the reaction space. With the reaction and reaction space established, the synthetic workflow was designed, and the robot was programmed accordingly. A flow diagram of the synthetic workflow and the script for the robot can be found in the ESI (Fig. S8†). As the robot did not have a module for handling solids, solid reagents were introduced as stock solutions. The sugars were dissolved in DMF and the benzoic anhydride in MeCN (4 M). Hence, it should be stressed that the optimal solvent in all cases is a solvent mixture with a minimum of two components, as a fixed amount of DMF and MeCN will be added to the mixture from these stock solutions. For the closed-loops utilizing the transfer learning approach an eighth parameter was included in the reaction space; “sugar”. This parameter was constrained to the sugar used for the specific reaction optimized, but it allowed for the introduction of previous data from optimizations of benzoylation of different sugars into the database prior to running the closed-loop.

**Table 1:** Descriptors used for principal component analysis and their main source^a^

Descriptor(s)	Source
Molecular weight, density, refractive index melting point, boiling point, log *P*, dipole moment	Stenutz^42^
Polar topological surface area	PubChem^43^
p*K*_aH_	p*K*_a_ data compiled by R. Williams^44^

a In some cases the descriptor value was not available at the specified source and was found elsewhere. A detailed overview of descriptors and sources can be found in the ESI (Tabel S2).

### Initial closed-loops

**Fig. 3:**
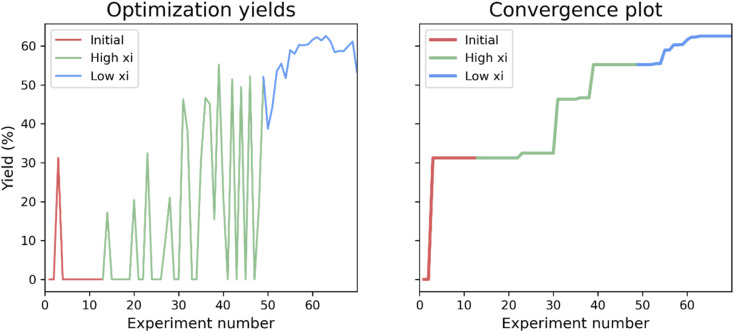
Results from the initial closed-loop optimization (CL1). The reaction optimized can be found in Fig. 2A. High xi = 1.0, low xi = 0.01 and xi changeover = 48. Number of initial points proposed using LHS = 12.

First, a closed-loop optimization of the reaction with the reaction space shown in Fig. 2A was carried out (CL1). The results are shown in Fig. 3. It is seen that out of the first initial 12 experiments proposed using LHS only one experiment gives a non-zero yield. A steady increase in the yield is observed after 30 experiments and convergence seems to be reached after 63 experiments. The larger fluctuations observed in the plot of the yield as a function of experiment number (left plot) when using a higher xi value compared to a low xi-value is in accordance with the algorithm being more explorative. Whilst the smaller fluctuations observed when using a lower xi-value are in accordance with a more exploitive algorithm, where only minor changes are made to the reaction conditions, resulting in only small changes in the yield. The optimal conditions found by the ReactionOptimizer give a yield of 63% and can be found in Table 2, entry 1. The optimal conditions include benzoic anhydride as the benzoylating reagent and triethylamine as the base, both in excess. Interestingly, to the best of our knowledge, the combination of benzoic anhydride and triethylamine, without other additives, has not been used for regioselective benzoylations of carbohydrates before, thereby the ReactionOptimizer successfully located a novel and seemingly superior procedure. Since no precedence is found in the literature, this combination would likely be missed, had the optimization been carried out using chemical intuition.

**Table 2:** Optimized conditions for regioselective benzoylations of 6-OH of a β-glucoside from two different closed-loop optimization. Experimental details for the optimization which yielded the conditions in entry 1 can be found in Fig. 2, and experimental details for the optimization which yielded the conditions in entry 2 can be found in the ESI

Entry	Closed-loop	Exp. no.	Yield (%)	Bz reagent	Eq. Bz	Base	Eq. base	Conc. (M)	*t*_1_ (min)	Solvent
1	CL1	63	63	Bz_2_O	2.13	Et_3_N	16.7	0.113	3	MeCN/THF (1 : 1)
2	CL2	15	68	Bz_2_O^a^	2.88	Et_3_N^a^	30.0	0.120	20	THF^b^

a Fixed during the optimization.

b Solvent fixed to possible ratios of MeCN and THF during the optimization.

The optimal solvent is a 1 : 1 mixture of acetonitrile and THF. How to include solvents and solvent mixtures is an obstacle when designing reaction spaces for Bayesian optimization of chemical reactions, though some solutions to this have been described in the literature.^45^ Here we chose a sequential approach, and since the optimal solvent was found to be a mixture of two solvents a second closed-loop (CL2) was run. In this closed-loop the solvent composition was fixed to acetonitrile and THF, but the ratio of the two solvents was introduced as a new parameter. Further, the benzoylating reagent and base were fixed to benzoic anhydride and triethylamine, respectively. As the optimal conditions included the maximum amount of base in the reaction space, the maximum amount of base was increased from 16.7 to 30 equivalents. Instead of using initial random experiments, the algorithm was given the experimental data from the previous closed-loop. During this second closed-loop the yield was increased from 63% to 68%, the new set of optimal conditions can be found as entry 2 in Table 2. It is seen that the amount of base is increased to the new maximum, and, interestingly, the preferred solvent ratio is pure THF, which would also have been possible as the solvent in the first closed-loop. Whether the reason for THF not being the optimal solvent in the first closed-loop is a consequence of the increased amount of base possible, or if it is simply not located, is unknown. It has previously been noted that the xi-changeover leads to a trade-off between time and improvement. It is possible, that if the optimizer had carried out more experiments before changing the xi-value THF might have been located as the optimal solvent in the first closed-loop.

**Fig. 4:**
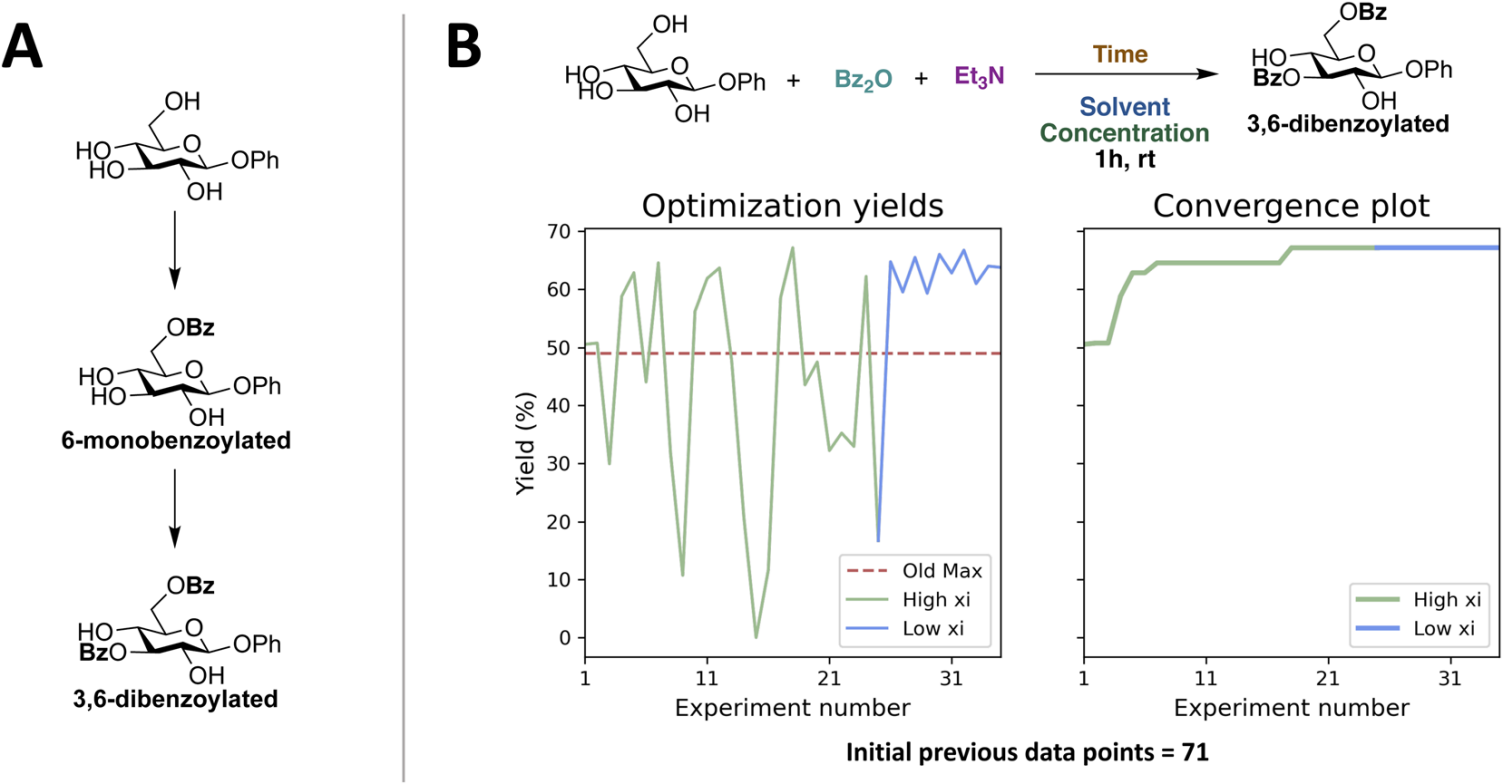
(A) Main species observed in reaction mixtures by UPLC. (B) Results from closed-loop optimization of dibenzoylation (CL3). High xi = 1.0, low xi = 0.02, and xi changeover = 24. Initial previous data points come from the reactions performed during CL1 and CL2. Old max describes the highest observed yield from the initial previous data points.

The optimal conditions include a substantial excess of benzoylating reagent (2.88 equivalents), which is surprising as this could lead to over-benzoylation. Upon analyzing the distribution of compounds present in the reaction mixtures it was evident that the main components were the reactant, the 6-monobenzoylated glucoside, and the 3,6-dibenzoylated glucoside (Fig. 4A). Pie charts showing the distribution of the three species in Fig. 4A for all experiments carried out during CL2 can be found in ESI (Fig. S12†). Hence, the dibenzoylation appears to be highly regioselective under conditions similar to those of CL2, and in the experiment containing most 3,6-dibenzoylated glucoside, the yield is 49%.

A third closed-loop (CL3) was designed in order to optimize for the 3,6-dibenzoylated product. A reaction space similar to that of CL2 was chosen to allow for the experiments previously carried out during CL2 to be included as initial data. The results are shown in Fig. 4B. In general, high yields are observed and convergence is reached after 18 experiments, with a yield of 67% of the 3,6-*O*-benzoylated product. This is an improvement compared to CL1, where initial experiments were low yielding, and it took 63 experiments to reach convergence. This improvement might be ascribed to two important differences. First, the reaction space has been narrowed down since the benzoylating reagent and base have been fixed, which would make the optimization easier by decreasing the reaction space. Further, the use of 71 initial previous data points instead of 12 random initial experiments would give the Bayesian optimization algorithm a much better starting point. To further investigate the importance of initial previous data we decided to design a transfer learning approach.

### Optimization using transfer learning

The effect of including previous data from the exact same reaction is not very intriguing, since it is unlikely a lot of previous data can be found on that exact reaction one is optimizing. Therefore, we developed a transfer learning approach, where data from past tasks is used to accelerate the current, *i.e.* optimization results from one reaction is used for the optimization of different, but related reaction. In recent years, some examples of using transfer learning approaches to improve Bayesian optimization have been described.^46,47^ However, to the best of our knowledge, transfer learning has not been applied to Bayesian optimization of chemical reactions. Though, transfer learning has been shown to successfully accelerate reaction optimization performed by random forest classifiers^48^ and neural processes combined with Bayesian optimization.^49^ We chose a simple approach where data from previous optimizations are included by introducing a new categorical variable, which allowed us to build initial optimization models using knowledge from previous optimizations, but without having to make changes to the algorithm. Data from other optimization will be referred to as “initial previous” data. It seems reasonable that the optimal conditions for one substrate are not necessarily optimal for a different substrate in all cases. However, the substrate specificity is often neglected, since performing reaction optimization on each substrate would be a tedious and time-consuming task, and in most cases, the effect is most likely minor. Though, within carbohydrate chemistry substrate specificity is a common problem due to the complex nature of carbohydrates caused by the many functional groups and stereogenic centers. Hence, substrate specific optimization could be beneficial in some cases, especially if the optimizations can be performed quickly by utilizing previous optimization data.

**Fig. 5:**
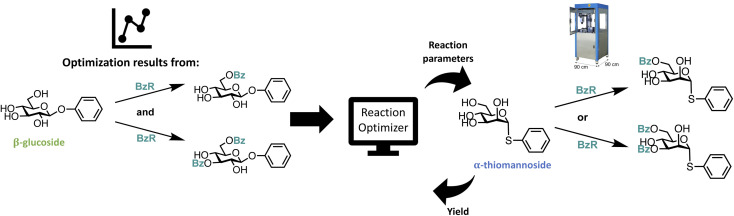
Graphical illustration of the transfer learning approach. The optimization data generated for regioselective benzoylation of a β-glucoside is used for optimizing regioselective benzoylations of an α-thiomannoside.

**Fig. 6:**
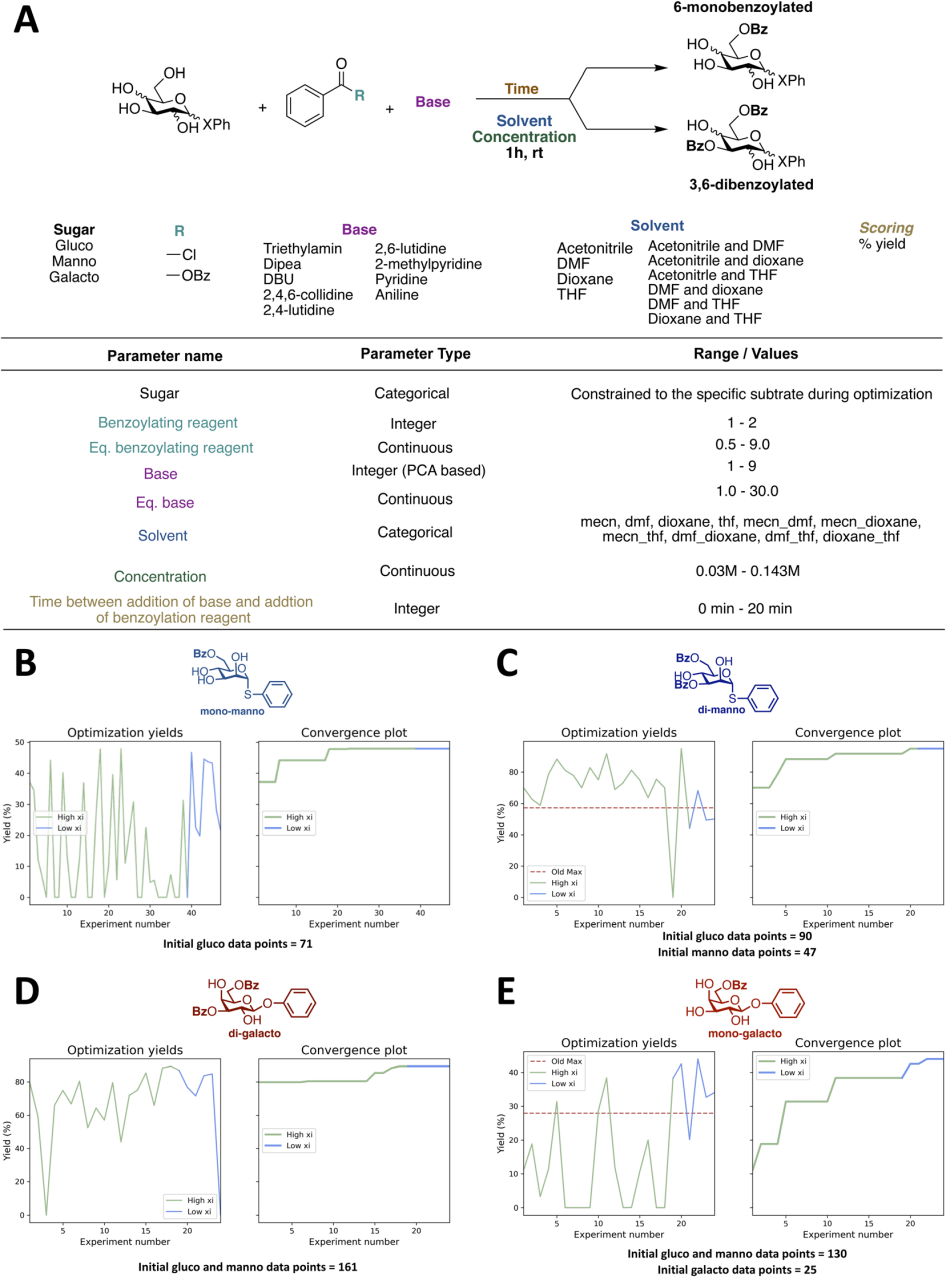
(A) Reaction space for closed-loop optimization using transfer learning. (B) Results from closed-loop optimization of monobenzoylation of an α-thiomannoside. (C) Results from closed-loop optimization of dibenzoylation of an α-thiomannoside. (D) Results from closed-loop optimization of dibenzoylation of a β-galactoside. (E) Results from closed-loop optimization of monobenzoylation of a β-galactoside.

We decided to investigate the effect of including the data obtained from the initial optimizations of the benzoylation of the β-glucoside for a similar reaction, but with a different substrate. This transfer learning approach is illustrated in Fig. 5. The first new substrate is an α-thiomannoside and the key differences from the β-glucoside are the anomeric configuration, the configuration at C-2, and the exchange of oxygen to sulfur. Thioglycosides are an important class of glycosides since they can be utilized as glycosyl donors.^50^ The reaction space can be seen in Fig. 6A, and the result from optimizing the monobenzoylation of the α-thiomannoside is shown in Fig. 6B. In total 71 reaction conditions and yields (data points) from the β-glucoside optimizations were provided by the ReactionOptimizer as “initial previous” data, instead of performing initial random experiments. Convergence is reached after only 23 experiments, compared to 63 experiments for the initial closed loop without transfer learning. Hence, it is a significant improvement, and it should be noted that the reaction space has been increased from the initial closed-loop optimization (Fig. 2) to the closed-loop optimizations (Fig. 6), as the ranges for the continuous parameters have been expanded. Similarly, the dibenzoylation of the α-thiomannoside was optimized and the results are shown in Fig. 6C.

Besides initial previous data from the β-glucoside optimizations, the data obtained for the optimization of the monobenzoylation of the α-thiomannoside was also provided to the ReactionOptimizer. Again, high yields are observed for most proposed conditions, and convergence is observed after 20 experiments (Fig. 6C).

Encouraged by these results we decided to investigate how the transfer learning approach would influence the optimization of benzoylation reactions of a third substrate, where both the optimization data from the β-glucoside optimizations and the α-thiomannoside were included as previous initial data. For this a β-galactoside was chosen as the substrate. This time we started with optimizing the dibenzoylation to make sure the fast convergence for the closed-loop optimization of the dibenzoylation of the α-thiomannoside was not a consequence of using previous data obtained from the specific substrate. Further, the second benzoylation is suspected to be more substrate specific as differences between the secondary alcohols are less pronounced than the difference between the primary alcohol and the secondaries, thereby the second benzoylation is more likely to be influenced by changes in the substrate's configuration. Albeit, as seen from Fig. 6D, the general picture of the optimization is very similar to that of the dibenzoylation optimization of the α-thiomannoside (Fig. 6C). Last, using all the gathered optimization data the monobenzoylation of the β-galactoside was optimized (Fig. 6E). No significant change in convergence time is observed between the closed-loop optimizations of the mono and dibenzoylation of the α-thiomannoside (23 and 20 experiments) and the corresponding closed-loop optimization for the β-galactoside (17 and 21 experiments). This indicates that initial previous data is beneficial, but that the amount of initial previous data can be saturated so that adding more data will no longer improve the convergence time. It is seen that in general, the proposed reaction conditions give yields, which are comparable to most existing literature procedures, for similar reactions.^11,16,23,26,31,34–36,51–53^ Moderately higher yielding procedures can be found in the literature^11,16,23,26,31,34–36,51–53^ for the synthesis of the compound, however, the simplicity and speed of our new procedures makes this benzoylated sugars more easily accessible.

### Optimal reaction conditions

The optimal conditions found in each closed-loop are shown in Table 3. All conditions except entry 4 include benzoic anhydride as the benzoylating reagent. Interestingly, entries 4 and 5 are from the same closed-loop and give the same yield but with very different conditions. The optimal reagents in entry 4 are benzoyl chloride and 2,6-lutidine, whereas for entry 4 it is benzoic anhydride and triethylamine. Hence, the ReactionOptimizer has been able to find two different optima within the same closed-loop. For all the conditions with benzoic anhydride, as the benzoylations reagent, the optimal base is found to be triethylamine, except for entry 7 (dibenzoylation of mannoside), where the preferred base is DIPEA. In general, the optimal conditions include large amounts of base and high concentrations. Surprisingly, an excess of benzoylating reagent is observed for all the conditions in Table 3. The optimal equivalent of benzoylating reagent for the monobenzoylation are 2.9, 3.7, and 4.2 (entries 2, 5, and 8, respectively), and for the dibenzoylations, the optimal equivalents of benzoylating reagent are 5.9, 8.1, and 8.2 (entries 3, 6, and 7). Thus, noteworthy differences in the optimal amount of benzoylating reagent are observed for the different substrates, with exception of entries 6 and 7. The large amounts of benzoylating reagent observed might be due to the relatively short fixed reaction time of 1 hour. Furthermore, the optimal solvent also seems to be substrate dependent, as three different solvents are selected as the optimal solvent: a mixture of THF and acetonitrile, pure THF, or dioxane. In conclusion, most of the optimal conditions have some similarities but also significant differences, consequently, substrate specificity is to be considered within carbohydrate chemistry and organic synthesis. It is seen that the newly discovered combination of triethylamine and benzoic anhydride is optimal in most of both the mono and dibenzoylations.

**Table 3:** Optimized conditions for regioselective benzoylations

Entry	Closed-loop	Product	Exp. no.	Bz Reagent	Eq. Bz	Base	Eq. base	Conc. (M)	*t*_1_ (min)	Solvent	Cl Yield (%)	Yield^b^ (%)
1	CL1	Mono-gluco	63	Bz_2_O	2.1	Et_3_N	16.7	0.113	3	MeCN/THF (1 : 1)	63%	n.d^c^
2	CL2^a^	Mono-gluco	15	Bz_2_O	2.9	Et_3_N^a^	30.0	0.120	20	THF	68%	47% (72%)^d^
3	CL3^a^	di-gluco	18	Bz_2_O	5.9	Et_3_N	24.8	0.140	19	MeCN/THF (88 : 12)	67%	64%
4	CL4	Mono-manno	23	BzCl	6.9	2,6-Lutidine	29.2	0.044	2	MeCN/THF (1 : 1)	48%	n.d.^e^
5	CL4	Mono-manno	18	Bz_2_O	3.7	Et_3_N	25.1	0.117	19	THF	48%	43%
6	CL5	Di-manno	20	Bz_2_O	8.1	Et_3_N	11.5	0.142	15	Dioxane	95%	57%
7	CL6	Di-galacto	17	Bz_2_O	8.2	DIPEA	29.1	0.122	3	THF	89%	59%
8	CL7	Mono-galacto	21	Bz_2_O	4.2	Et_3_N	26.0	0.094	4	Dioxane	44%	32% (54%)^d^^,^^e^

a Reduced reaction space with fixed benzoylating reagent and base. The solvent components were fixed to MeCN and THF, but the ratio varied.

b Manually determined yield after flash column chromatography.

c Synthesis was not attempted as better conditions were found in CL2.

d Crude yield.

e Rest of dioxane observed in crude NMR.

Next, we were interested in investigating the transferability of the procedures to our everyday manual laboratory work. This was done by carrying out the experiments in Table 3 without using the robot and with standard laboratory equipment. A procedure similar to the experimental procedure encoded in the robot was followed. However, one major change to the automated procedure was necessary as the reactions were quenched using methanol by the robot. This was adequate for the UPLC analysis, but when trying to purify the compounds by flash column chromatography the ester biproduct was co-eluting, and as excess benzoylating was required in all cases, methyl benzoate ended up being the major component of the crude reaction mixture, which was not desirable. We, therefore, chose to quench the reactions with dimethylaminopropylamine (DMAPA) instead of methanol, as this gives rise to benzoic acid and an amide as byproducts, which both can be removed by an aqueous workup.^54^ Since the sugars and benzoic anhydride were added as stock solutions during the automated experiments, stock solutions were also utilized during the manual experiments. The detailed manual experimental procedure can be found in ESI.† It was possible to carry out two experiments a day, whereas the automated setup approximately carried out 12 experiments within 24 hours. The results from careering out the manual experiments can be found in the last column in Table 3. In some cases, only the sugar of interest and very minor residues of other carbohydrates were observed from crude NMR, the crude yield for these reactions are therefore included in parenthesis (spectra of the crude products can be found in ESI†). For entries 2, 3, 5, and 8 the manually obtained yields are comparable to the UPLC yields obtained from the closed-loops. Though, for entries 4, 6, and 7 the manually obtained yields are significantly lower. When caring out the manual experiment in entry 4 only a trace amount of the desired product was observed by NMR and only minor conversion of the starting material was observed. Upon manual addition of the benzoyl chloride, vapor formation was observed over the reaction mixture, which is suspected to be caused by HCl gas formation. The discrepancy between the UPLC yield and the manually obtained yield could be a consequence of the differences between the manual and automated setup. The reactions are carried out by the robot in vials, which are open for a significant amount of time after adding the benzoyl chloride. Contrary, the manually performed reactions are carried out in a round-bottomed flask with a septum and a balloon filled with nitrogen, which might cause a difference in reaction conditions, atmosphere, and, consequently, the yield. The drop in yield for entries 6 and 7, which are the optimal conditions for the dibenzoylation of the mannoside and galactoside, respectively, could be due to benzoyl group migration. From the crude spectra, a complex mixture of benzoylated species is observed, unlike what was observed by UPLC analysis in the automated experiments. Therefore, we suspect that the new quenching procedure might facilitate migration of the benzoyl groups. Migration of acyl groups is a known problem within carbohydrate chemistry^55^ and is more likely if the kinetic product is higher in energy than the thermodynamic products. In addition, the reactions performed by the automated setup are run under ambient conditions, even though no effort has been made to dry the solvents or glassware in the case of the manually performed experiments differences in atmosphere composition and water residue are expected, which could impact the reactions. Furthermore, the automated reactions have been performed on a 50 mg scale, whereas the manually performed reactions have been performed on a 250 mg scale. The deviation between the manual experiments and the ones performed by the robot seems to be a consequence of parameters that impact the reaction, but which have not been considered as variables, in the sense, that the developed conditions are not scale and equipment independent.

## Conclusion

In conclusion, we have optimized the selective protection of fully unprotected glycosides in a fully automated closed-loop setup. In addition, we have shown that the methodology can be expanded to alternative substrates with very little additional effort by using a novel transfer learning approach, where previous data from optimizations of similar reactions are included by introducing a new categorical variable. In this way, the optimizations can be accelerated, though at some point the algorithm will be saturated with previous data and adding more data will not have an influence. Using the automated setup, we were able to locate a set of optimal reaction conditions involving triethylamine and benzoic anhydride, which, to the best of our knowledge, has not previously been applied for regioselective benzoylations of unprotected glycosides without further additives. The final procedures are fast, simple, and carried out under ambient conditions without specialized equipment. Further, the procedures only include reagents present in most chemistry laboratories. We also found that it is important to consider the transferability from the automated setup to the laboratory early in the project design, as the automated setup only considers the reaction, and optimizing the process, which will be the next step, can be complicated by limitations arising from the initial design of the automated setup. The study also gave an insight into the importance of considering substrate specificity. Both similarities and notable differences between the optimal reaction conditions were observed for each carbohydrate substrate, which suggests that substrate specificity is important to consider when performing reaction optimization.

## Data availability

The experimental methods and datasets that support this article are available in the ESI.†

## Author contributions

NVF: project design, data acquisition, data analysis, synthesis, writing of scripts, and writing of the original draft. CMP: supervision and funding acquisition. RHT: supervision.

## Conflicts of interest

There are no conflicts to declare.

## Supplementary Material

SC-014-D3SC01261A-s001

SC-014-D3SC01261A-s002
