# Supplementary material for: Substrate specific closed-loop optimization of carbohydrate protective group chemistry using Bayesian optimization and transfer learning
Source: Chem Sci. 2023 May 18;14(23):6319–29. doi: 10.1039/d3sc01261a (PMC10266441; doi:10.1039/d3sc01261a)
Supplement: SC-014-D3SC01261A-s002 [file SC-014-D3SC01261A-s002.pdf]

Electronic Supplementary Material (ESI) for Chemical Science.
This journal is © The Royal Society of Chemistry 2023

Substrate Specific Closed-loop Optimization of Carbohydrate Protective Group Chemistry Using Bayesian Optimization and Transfer Learning

Natasha Videcrantz Faurschou, [a] Rolf Hejle Taaning [b] and Christian Marcus Pedersen[a]*

Abstract: A new way of performing reaction optimization within carbohydrate chemistry is presented. This is done by performing closed-loop optimization of regioselective benzoylation of unprotected glycosides using Bayesian optimization. Both 6-*O*- monobenzoylations and 3,6-*O*-dibenzoylations of three different monosaccharides are optimized. A novel transfer learning approach, where data from previous optimizations of different substrates is used to speed up the optimizations, has also been developed. The optimal conditions found by the Bayesian Optimization algorithm provide new insight into substrate specificity, as the conditions found are significantly different. In most cases, the optimal conditions include Et_3N and benzoic anhydride, a new reagent combination for these reactions, discovered by the algorithm, demonstrating the power of this concept to widen the chemical space. Further, the developed procedures include ambient conditions and short reaction times.

Table of Contents

Experimental Procedures **3**

Compound Characterization List *3*

General experimental methods and equipment *3*

General experimental procedure manual synthesis of suggested experiments *3*

Characterization *4*
- **Phenyl 6-O-benzoyl-β-D-glucopyranoside** 4
- **Phenyl 3,6-di-O-benzoyl-β-D-glucopyranoside** 5
- **Phenyl 6-O-benzoyl-α-D-thiomannopyranoside** 5
- **Phenyl 3,6-di-O-benzoyl-α-D-thiomannopyranoside** 6
- **Phenyl 6-O-benzoyl-β-D-galactopyranoside** 6
- **Phenyl 3,6-di-O-benzoyl-β-D-galactopyranoside** 7

NMR spectra *8*

Results and Discussion **22**

Benchmark study *22*
- Setup 22
- Parameter tuning 24

Robot setup *26*
- Photo from inside the robot 26
- Flow diagram of script 26
- Detailed description of quenching and HPLC 27
- Chemspeed Script 27

Standard curves *31*

Example of HPLC chromatogram *34*

Base descriptor sources *34*

Partial Dependency Plots *35*
- Cl1 – Mono-gluco 35
- CL2 – Mono-gluco 36
- CL3 - Digluco 37
- Cl4 – Mono-manno 38
- Cl5 – Di-manno 39
- CL6 – Di-galacto 40
- CL7 – Mono-galacto 41

Closed-Loop 2 .... *41*
Reaction space 41
Results 42
Pie charts of compound distribution 42

Theory **44**

References **46**

Experimental Procedures

Compound Characterization List

No.	Structure	New	^{1}H NMR	^{13}C NMR	COSY	HSQC	HRMS	R$_f$
**1**	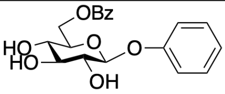	+*	+	+	+	+	+	+
**2**	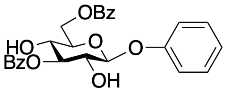	−	+	+	+	+	+	+
**3**	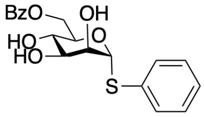	+	+	+	+	+	+	+
**4**	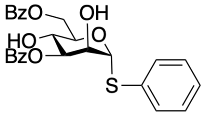	+	+	+	+	+	+	+
**5**	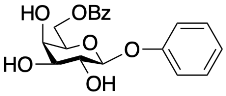	+	+	+	+	+	+	+
**6**	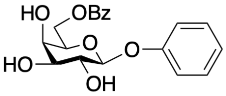	+	+	+	+	+	+	+

Note: + equals true or included and − equals false or not included.
* Compound has not been characterized using NMR, but melting point and elemental analysis have been reported. [2

General experimental methods and equipment

All chemicals have been used as purchased from the supplier without further purification. Solvents were HPLC grade and were used directly from the flask without any attempt of further drying them. Reactions were monitored using TLC using aluminum sheets coated with silica (Merck F_{254}-plates). The spots were visualized using UV and staining with 10% H_2SO_4 in ethanol. Purification using flash chromatography or by a Buchi Pure C-815 chromatography instrument.

HRMS values were obtained using MALDI, the spectra were recorded using a Bruker SolariX XR 7T ESI/MALDI-FT-ICR-MS instrument. ^{1}H-NMR and ^{13}C-NMR spectra were recorded at 500 MHz and 125 MHz respectively, using a Bruker 500 MHz Ultra Shield Plus instrument with a cryoprobe.

General experimental procedure manual synthesis of suggested experiments

250 mg of the specific carbohydrate was dissolved in 255 μL DMF in a pear-shaped flask. While stirring, the solvent was added, followed by the base. After waiting for t1, the benzoylating reagent was added, either neat (benzoyl chloride) or as a 4M solution in MeCN (benzoic anhydride). The reaction was stirred for 1 hour, and then quenched with DMAPA. After stirring the quenched solution for 10 minutes, 10 mL of EtOAc was added to the solution, and the solution was washed thrice with 1M HCl (3x10ml), once with 1M NaOH(10 ml) and once with brine(10 ml). The organic phase was concentrated *in vacou* and purified by column chromatography ($CDCl_3$ and MeOH).

Table S1. Specific experimental for manual synthesis.

Sugar	Bz Reagent	Base	Solvent	t1 (min)	V DMAPA (mL)	Yield
mono-gluco (243 mg, 948 µmol)	Bz_2O[b] (683 µL, 2.73 mmol)	Et_3N (3.97 mL, 28.45 mmol)	THF (3.1 mL)	20	0.40	47% (72%)[a]
di-gluco (245 mg, 956 µmol)	Bz_2O[b] (1.44 mL, 5.76 mmol)	Et_3N (3.02 mL, 24.19 mmol)	88:12 MeCN/THF (1.9 mL)	19	0.55	64 %
mono-manno (250 mg, 918 µmol)	BzCl (732 µL, 6.30 mmol)	2,6-lutidine (3.96 mL, 26.81 mmol)	1:1 MeCN/THF (15 mL)	2	0.80	Trace amount
mono-manno (246 mg, 903.37µmol)	Bz_2O[b] (845 µL, 3.38mmol)	Et_3N (3.21 mL, 23.03 mmol)	THF (3.6 mL)	19	0.46	43%
di-manno (247 mg, 907 µmol)	Bz_2O[b] (1.85mL, 7.39 µl)	Et_3N (1.47 mL, 10.53 mmol)	Dioxane (2.9 mL)	15	1.0	57%
di-galacto (247 mg, 964 µmol)	Bz_2O[b] (2.00 mL, 8.00 mol)	DIPEA (5.00 mL, 28.68 mmol)	THF (0.75 mL)	3	1.0	59%
mono-galacto	Bz_2O[b] (1.02 mL, 4.10 mol)	Et_3N (3.54 mL, 25.37mmol)	Dioxane (5.6 mL)	4	0.55	32% (54%)[a]

[a] Crude yield. [b] 4M in MeCN

Characterization

Phenyl 6-*O*-benzoyl-β-D-glucopyranoside

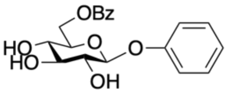

1

^{1}**H NMR** (500 MHz, DMSO) δ: 8.09 - 7.93 (m, 2H, Ar(Bz)), 7.69 (td, *J* = 7.2, 1.1 Hz, 1H, Ar(Bz)), 7.57 (t, *J* = 7.8 Hz, 2H, Ar(Bz)), 7.25 - 7.14 (m, 2H, Ar(Ph)), 7.05 - 6.99 (m, 2H, Ar(Ph)), 6.99 - 6.92 (m, 1H, Ar(Ph)), 5.41 (d, *J* = 4.9 Hz, 1H, OH), 5.39 (d, *J* = 5.3 Hz, 1H, OH), 5.24 (d, *J* = 4.6 Hz, 1H, OH), 4.95 (d, *J* = 7.4 Hz, 1H, H-1), 4.61 (dd, *J* = 11.7, 2.1 Hz, 1H, H-6a), 4.28 (dd, *J* = 11.7, 7.6 Hz, 1H, H-6b), 3.80 (ddd, *J* = 9.6, 7.5, 2.1 Hz, 1H, H-5), 3.40-3.24 (m, 3H, H-2, H-3, H-4).

^{13}**C NMR** (126 MHz, DMSO) δ: 165.5, 157.1, 133.4, 129.6, 129.2, 129.1, 128.7, 121.8, 116.1, 99.9, 76.4, 73.7, 73.2, 70.1, 64.3.

R$_f$: 1:9 (MeOH/$CDCl_3$) = 0.19.

HRMS (MALDI) m/z calcd for $C_{19}H_{21}O_7Na^+$ 383.11012; Found 383.10947

Phenyl 3,6-di-O-benzoyl-β-D-glucopyranoside

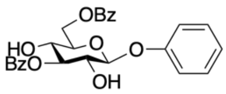

2

[1]**H NMR** (500 MHz, $CDCl_3$) δ: 8.10 (m, 2H, Ar(Bz)), 8.07 (m, 2H, Ar(Bz)), 7.69 – 7.55 (m, 2H, Ar(Bz)), 7.45 (m, 4H, Ar(Bz)), 7.27 – 7.17 (m, 2H, Ar(Ph)), 7.13 – 6.94 (m, 3H, Ar(Ph)), 5.38 (t, *J* = 9.2 Hz, 1H, H-3), 5.10 (d, *J* = 7.8 Hz, 1H, H-1), 4.76 (dd, *J* = 12.0, 2.3 Hz, 1H, $H\text{-}6_a$), 4.61 (dd, *J* = 12.0, 6.4 Hz, 1H, $H\text{-}6_b$), 4.01 (dd, *J* = 9.4, 7.7 Hz, 1H, H-2), 3.94 (ddd, *J* = 9.9, 6.4, 2.3 Hz, 1H, H-5), 3.85 (dd, *J* = 9.4, 9.4 Hz, 1H, H-4).

[13]**C NMR** (126 MHz, $CDCl_3$) δ: 167.8, 166.8, 157.0, 133.6, 133.3, 130.1, 129.9, 129.7, 129.5, 129.3, 128.5, 128.4, 123.1, 117.0, 100.9, 78.5, 74.5, 72.2, 69.6, 63.9.

$\mathbf{R_f}$: 1:9 (MeOH/$CDCl_3$) = 0.30

HRMS (MALDI) m/z calcd for $C_{26}H_{24}O_8Na^+$ 487.13634; Found : 487.13593

Phenyl 6-O-benzoyl-α-D-thiomannopyranoside

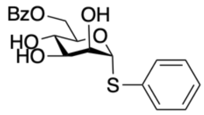

3

[1]**H NMR** (500 MHz, DMSO) δ: 7.91 - 7.83 (m, 1H), 7.72 - 7.64 (m, 1H), 7.55 - 7.49 (m, 1H), 7.48 - 7.43 (m, 1H), 7.26 - 7.14 (m, 2H), 5.42 (s, 1H, H-1), 5.31 - 5.23 (m, 2H, OH(*HO-4, HO-2*)), 4.99 (d, *J* = 5.7, 1H, OH(*HO-3*)), 4.60 (dd, *J* = 11.7, 1.9 Hz, 1H, H-6a), 4.34 (dd, *J* = 11.4, 7.5 Hz, 1H, H-6b), 4.18 (ddd, *J* = 9.6, 7.8, 2.5 Hz, 1H, H-5), 3.93 (m, 1H, H-2), 3.62 (t, *J* = 9.4 Hz, 1H, H-4), 3.54 (dd, *J* = 8.9, 5.7 Hz, 1H, H-3).

[13]**C NMR** (126 MHz, DMSO) δ: 165.5, 134.0, 133.3, 131.1, 129.6, 129.1, 128.9, 128.7, 127.1, 88.3, 72.0, 71.6, 71.3, 67.1, 64.4.

$\mathbf{R_f}$: 1:9 (MeOH/$CDCl_3$) = 0.19

HRMS (MALDI) m/z calcd for $C_{19}H_{20}O_6Na^+$ 399.08728; Found 399.08744

Phenyl 3,6-di-O-benzoyl-α-D-thiomannopyranoside

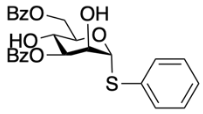

4

[1]**H NMR** (500 MHz, DMSO) δ: 8.14 - 8.03 (m, 2H, Ar(Bz)), 7.93 - 7.84 (m, 2H, Ar(Bz)), 7.76 - 7.64 (m, 2H, Ar(Bz)), 7.62 - 7.44 (m, 6H, Ar(Bz, Ph)), 7.35 - 7.19 (m, 3H, Ar(Ph)), 5.77 (d, *J* = 5.2, 1H, OH(*HO-2*)), 5.73 - 5.66 (m, 1H, OH(*HO-4*)), 5.54 (d, *J* = 1.8 Hz, 1H, H-1), 5.09 (dd, *J* = 9.7, 3.2 Hz, 1H, H-3), 4.74 - 4.61 (m, 1H, H-6a), 4.49 - 4.36 (m, 2H, H-6a, H-5), 4.30 - 4.21 (m, 1H, H-2), 4.14 - 4.01 (m, 1H, H-4).

[13]**C NMR** (126 MHz, DMSO) δ: 165.5, 133.4, 133.3, 131.3, 129.8, 129.5, 129.2, 129.0, 128.8, 128.7, 128.6, 128.5, 127.4, 87.8, 75.0, 72.1, 68.9, 64.4, 64.0. *One carbonyl signal is missing, possibly due to overlapping signals.*

R_f : 1:9 (MeOH/$CDCl_3$) = 0.33

HRMS (MALDI) m/z calcd for $C_{26}H_{24}O_7SNa^+$ 503.11349; Found 503.11366

Phenyl 6-*O*-benzoyl-β-D-galactopyranoside

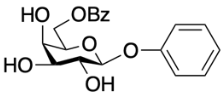

5

[1]**H NMR** (500 MHz, DMSO) δ: 7.99 (dd, *J* = 8.3, 1.4 Hz, 2H, Ar(Bz)), 7.74 – 7.67 (m, 1H, Ar(Bz)), 7.58 (t, *J* = 7.8 Hz, 2H, Ar(Bz)), 7.22 – 7.14 (m, 2H, Ar(Ph)), 7.06 – 7.00 (m, 2H, Ar(Ph)), 6.95 (tt, *J* = 7.4, 1.1 Hz, 1H, Ar(Ph)), 5.24 (d, *J* = 4.9 Hz, 1H, OH(C-2)), 4.98 (d, *J* = 5.6 Hz, 1H, OH(C3)), 4.94 – 4.79 (m, 2H, H-1, OH(C4)), 4.44 (dd, *J* = 11.4, 8.6 Hz, 1H, H-6_a), 4.38 (dd, *J* = 11.4, 3.8 Hz, 1H, H-6_b), 4.09 – 4.03 (m, 1H, H-5), 3.83 – 3.74 (m, 1H, H-4), 3.62 (ddd, *J* = 9.3, 7.4, 4.5 Hz, 1H, H-2), 3.49 (ddd, *J* = 9.3, 5.5, 3.3 Hz, 1H, H-3).

[13]**C NMR** (126 MHz, DMSO) δ: 165.5, 157.3, 133.4, 129.6, 129.2, 129.1, 128.7, 121.7, 116.1, 100.5 (C-1), 73.0, 72.5, 70.1, 68.4, 64.4.

R_f : 1:9 (MeOH/$CDCl_3$) = 0.21

HRMS (MALDI) m/z calcd for $C_{19}H_{21}O_7Na^+$ 383.11012; Found 383.10990

Phenyl 3,6-di-*O*-benzoyl-β-D-galactopyranoside

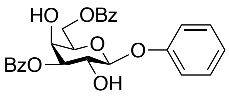

6

[1]**H NMR** (500 MHz, DMSO) δ: 8.08 (dt, *J* = 7.0, 1.4 Hz, 2H, Ar(Bz)), 8.00 (dt, *J* = 7.0, 1.3 Hz, 2H, Ar(Bz)), 7.79 – 7.66 (m, 2H, Ar(Bz)), 7.63 – 7.51 (m, 4H, Ar(Bz)), 7.25 – 7.16 (m, 2H, Ar(Ph)), 7.10 – 7.04 (m, 2H, Ar(Ph)), 7.02 – 6.94 (m, 1H, Ar(Ph)), 5.69 (d, *J* = 5.3 Hz, 1H, OH(C-2)), 5.44 (d, *J* = 5.8 Hz, 1H, OH(C-4)), 5.17 (d, *J* = 7.7 Hz, 1H, H-1), 5.04 (dd, *J* = 10.0, 3.5 Hz, 1H, H-3), 4.52 – 4.38 (m, 2H, H-6_a, H-6_b), 4.34 – 4.26 (m, 1H, H-5), 4.15 (m, 1H, H-4), 4.04 (m, 1H, H-2).

[13]**C NMR** (126 MHz, DMSO) δ: 165.5, 165.5, 157.0, 133.4, 133.3, 130.0, 129.6, 129.5, 129.2, 129.2, 128.8, 128.6, 121.9, 116.1, 100.0 (C-1), 76.4, 72.2, 67.5, 65.9, 64.0.

$\mathbf{R_f}$: 1:9 (MeOH/$CDCl_3$) = 0.43

HRMS (MALDI) m/z calcd for $C_{26}H_{24}O_8Na^+$ 487.13634; Found 487.13618

NMR spectra

Phenyl 6-*O*-benzoyl-β-D-glucopyranoside (1)

^{1}H NMR (500MHz, DMSO)

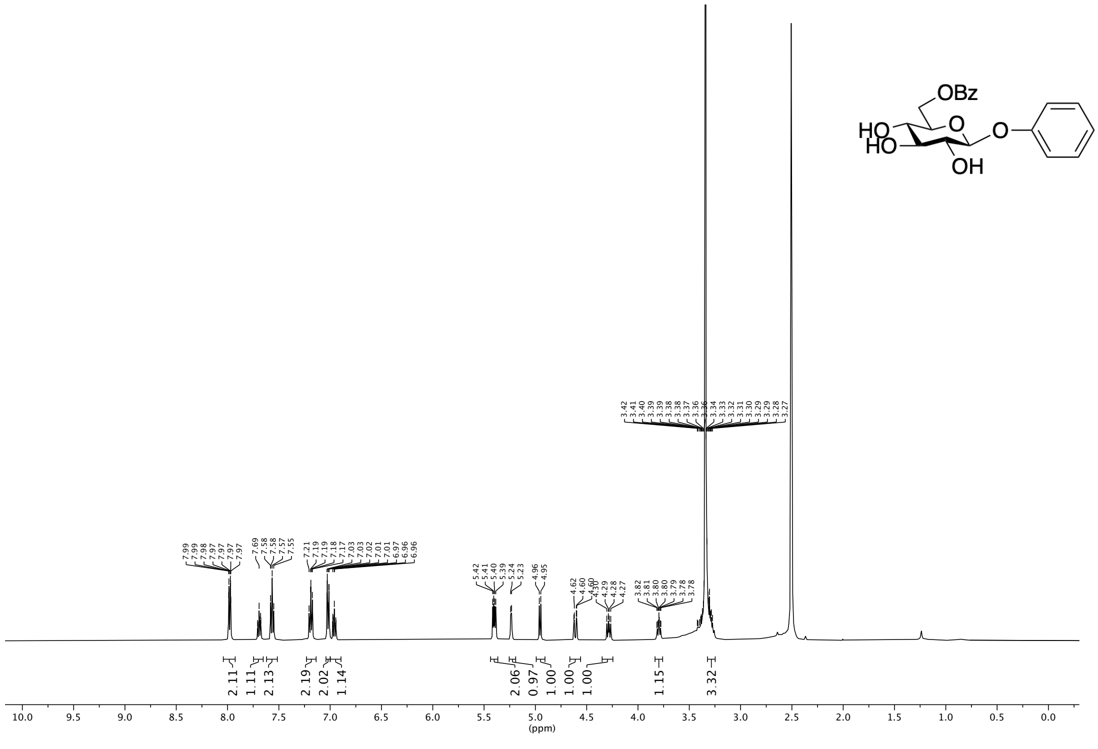

^{13}C NMR (126MHz, DMSO)

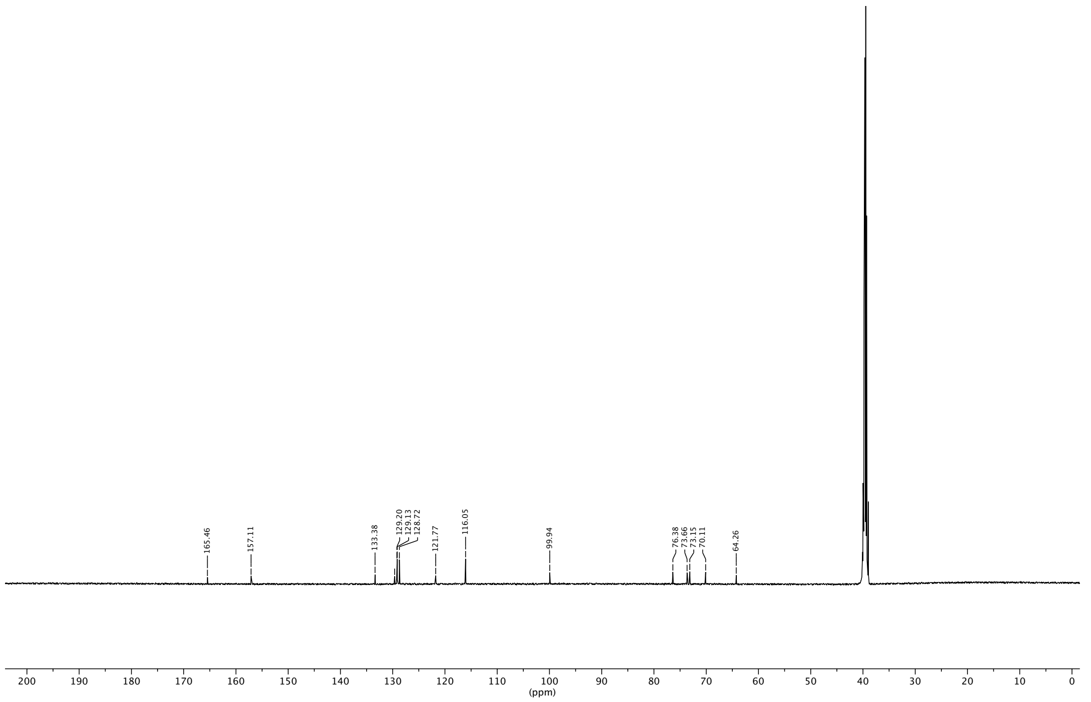

COSY ^{1}H NMR (500MHz, DMSO)

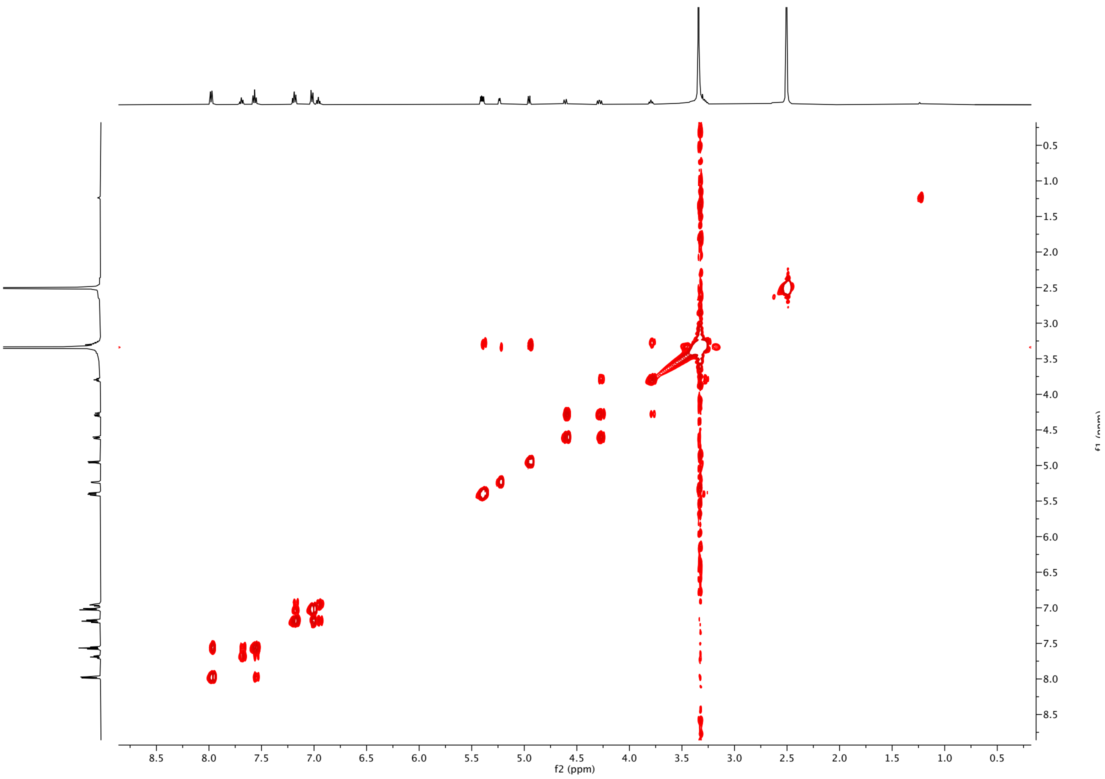

HSQC ^{1}H NMR (500MHz, DMSO), ^{13}C NMR (126MHz, DMSO)

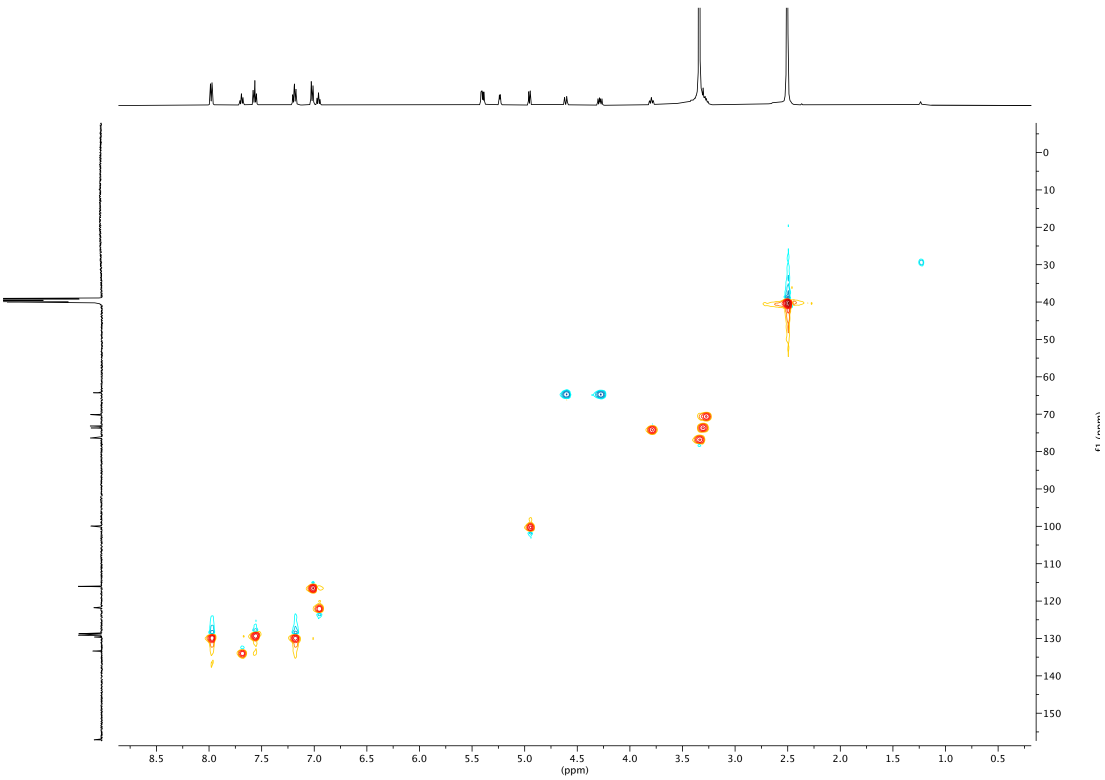

Phenyl 3,6-di-*O*-benzoyl-β-D-glucopyranoside (2)

^{1}H NMR (500MHz, $CDCl_3$)

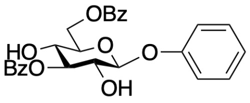

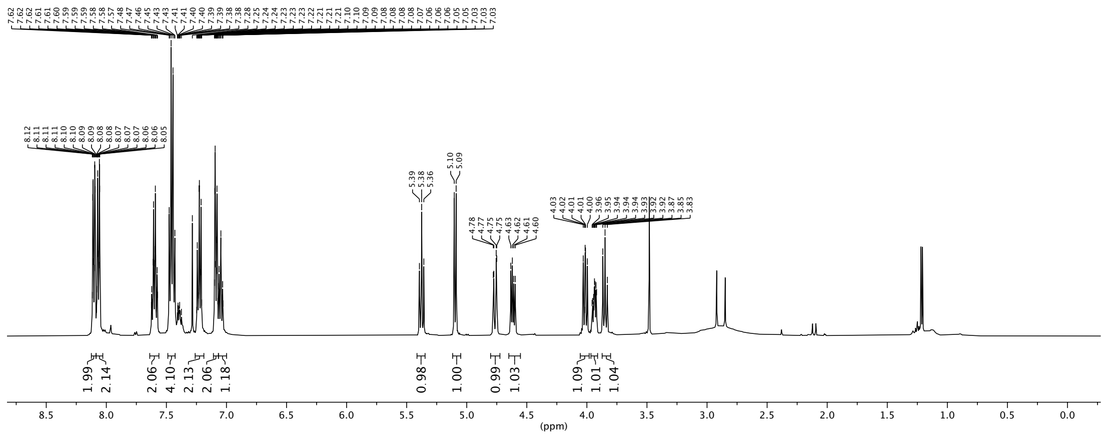

^{13}C NMR (126MHz, $CDCl_3$)

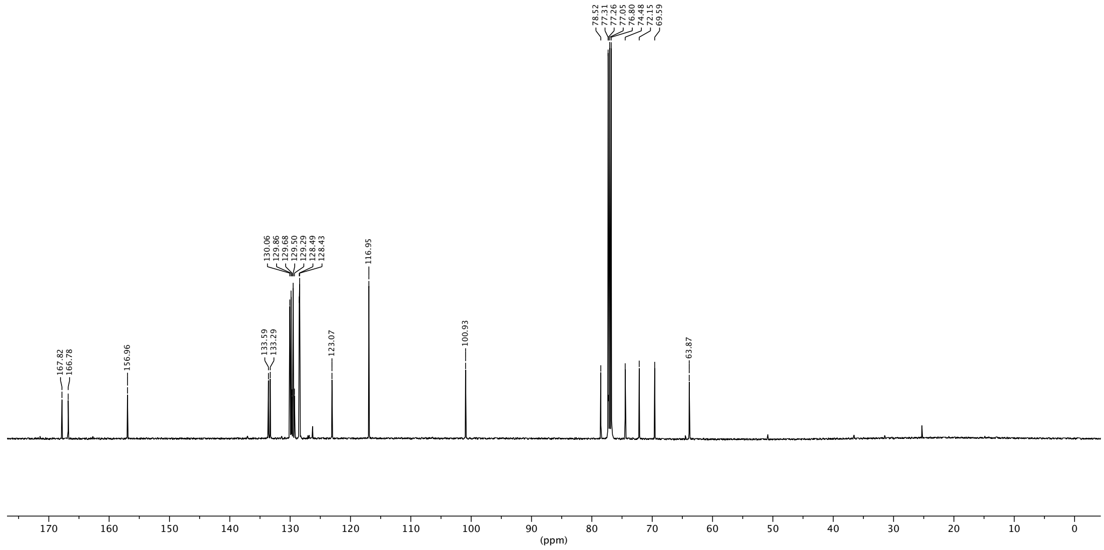

COSY ^{1}H NMR (500MHz, $CDCl_3$)

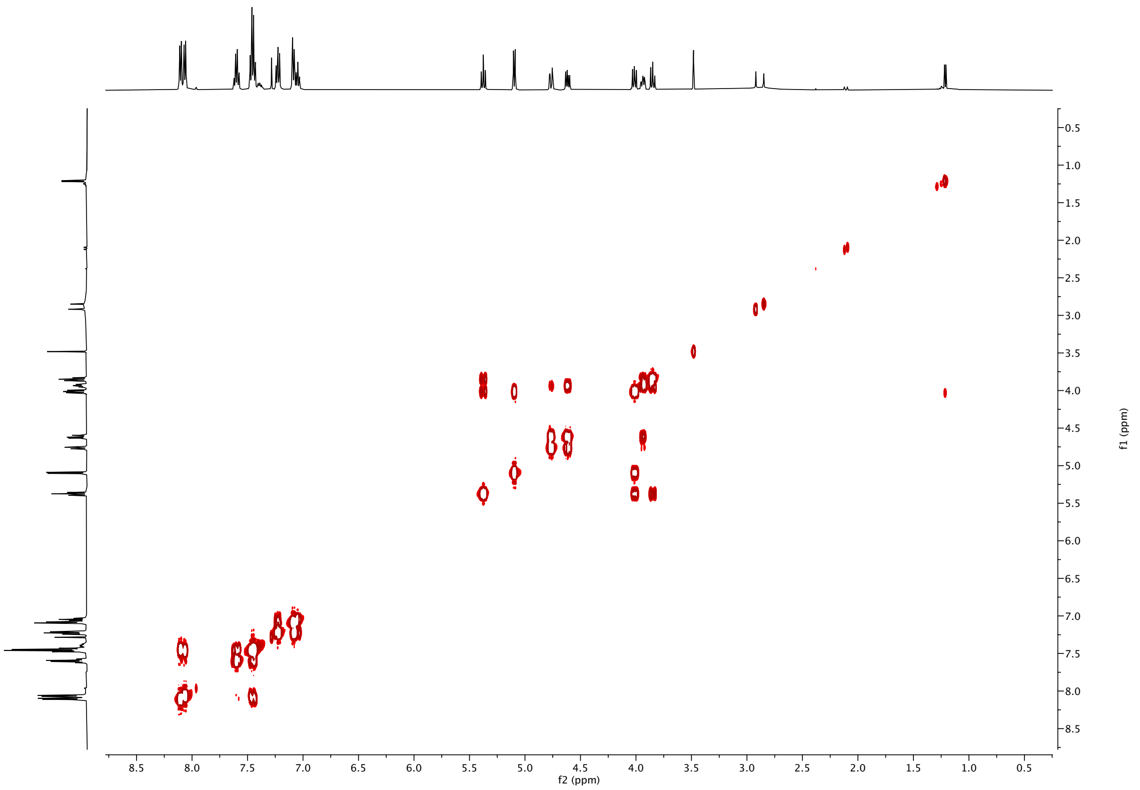

HSQC ^{1}H NMR (500MHz, $CDCl_3$), ^{13}C NMR (126MHz, $CDCl_3$)

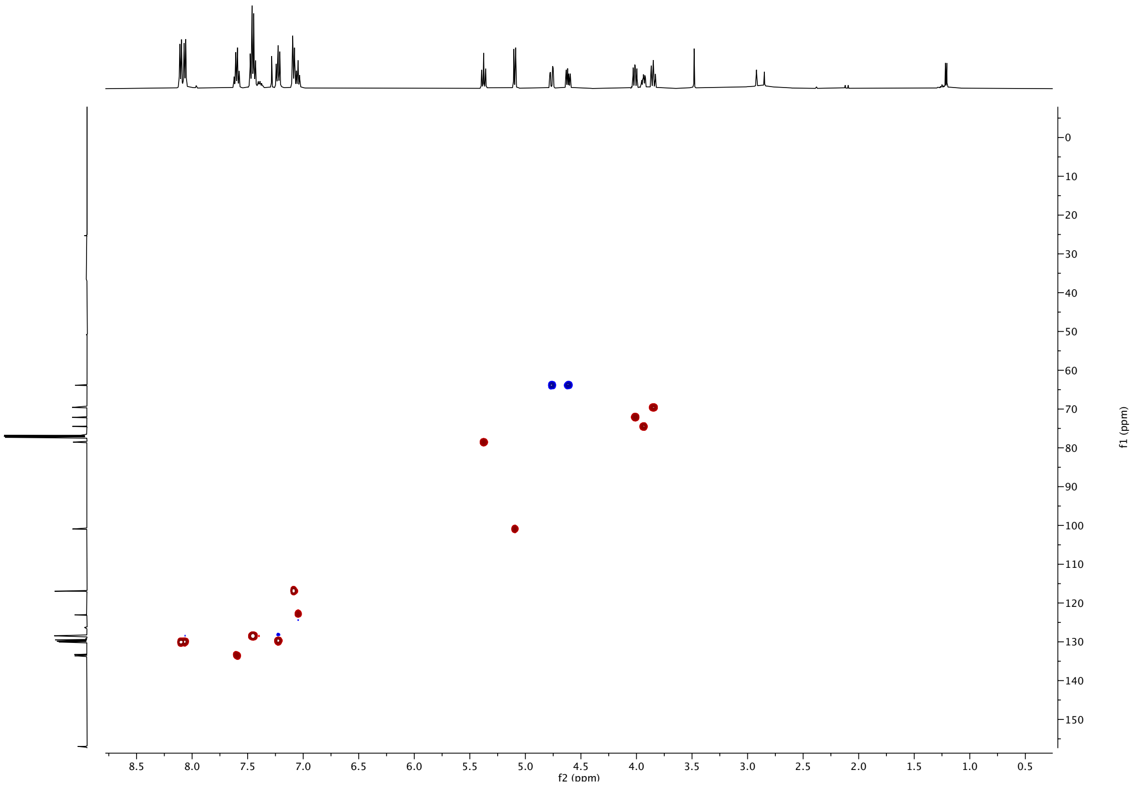

Phenyl 6-*O*-benzoyl-α-D-thiomannopyranoside (3)

^{1}H NMR (500MHz, DMSO)

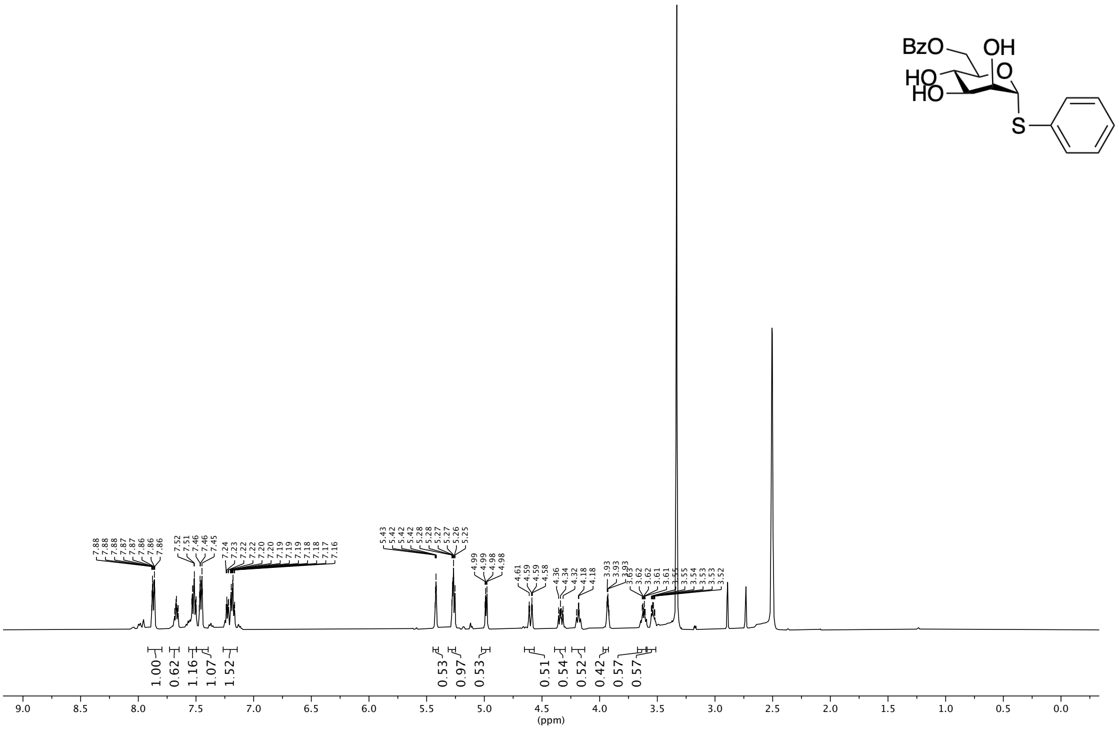

^{13}C NMR (126MHz, DMSO)

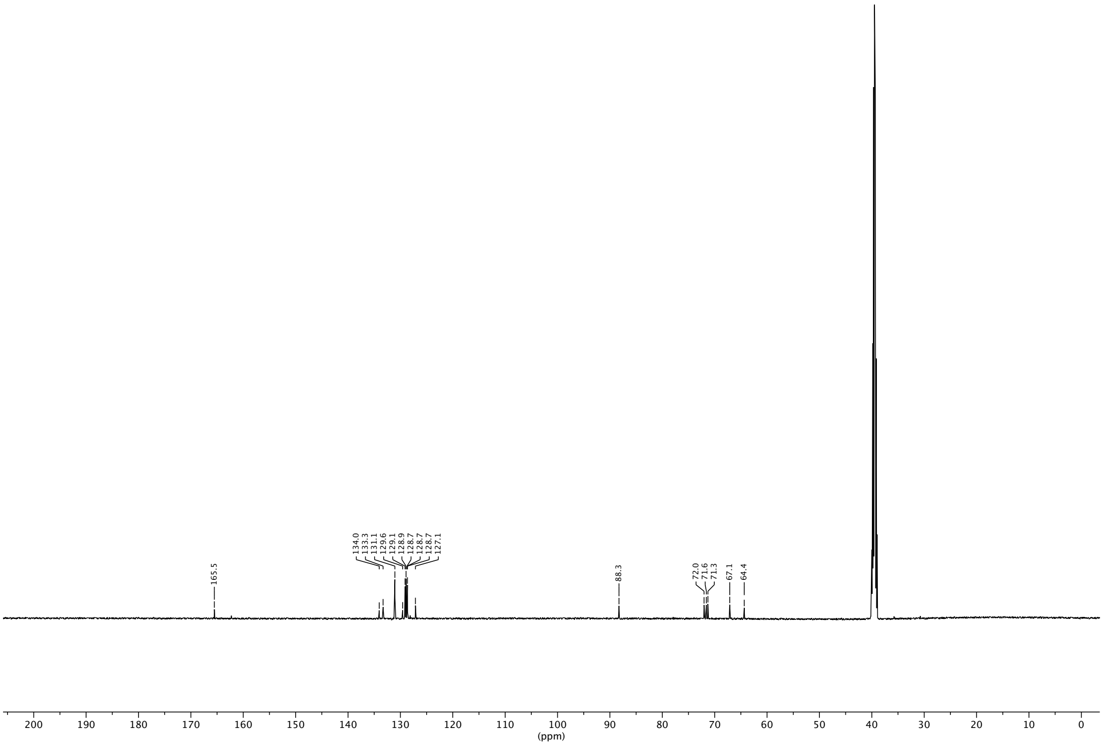

COSY ^{1}H NMR (500MHz, DMSO)

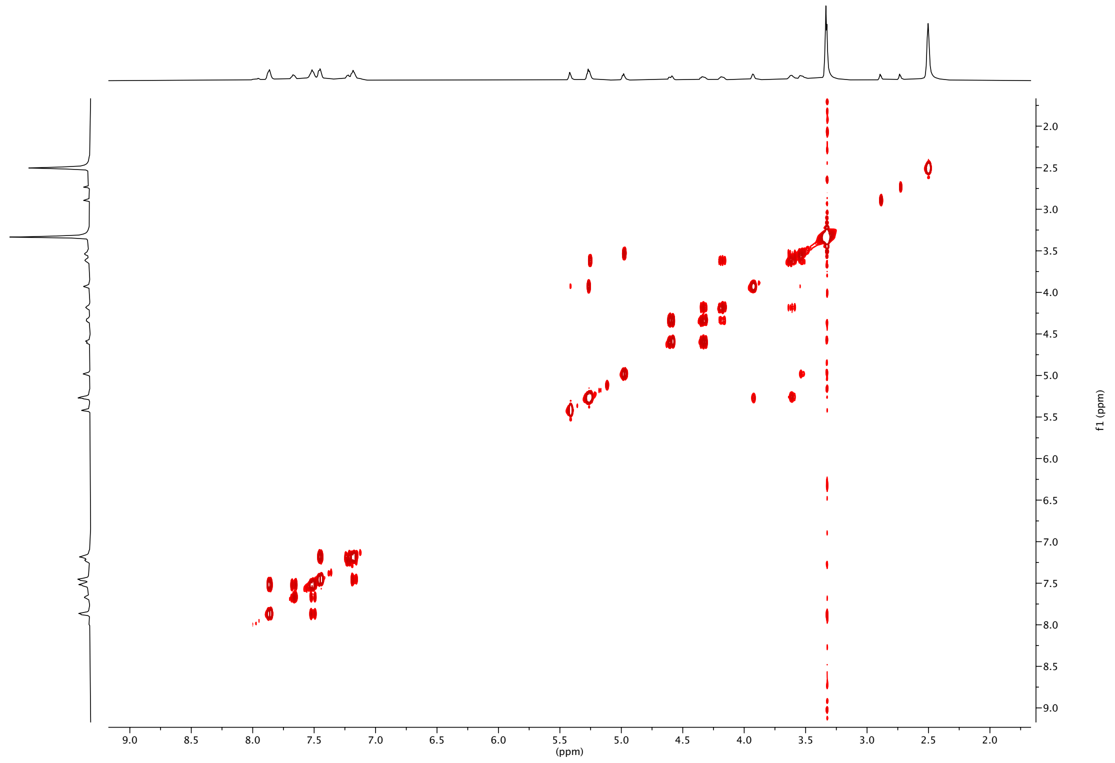

HSQC ^{1}H NMR (500MHz, DMSO), ^{13}C NMR (126MHz, DMSO)

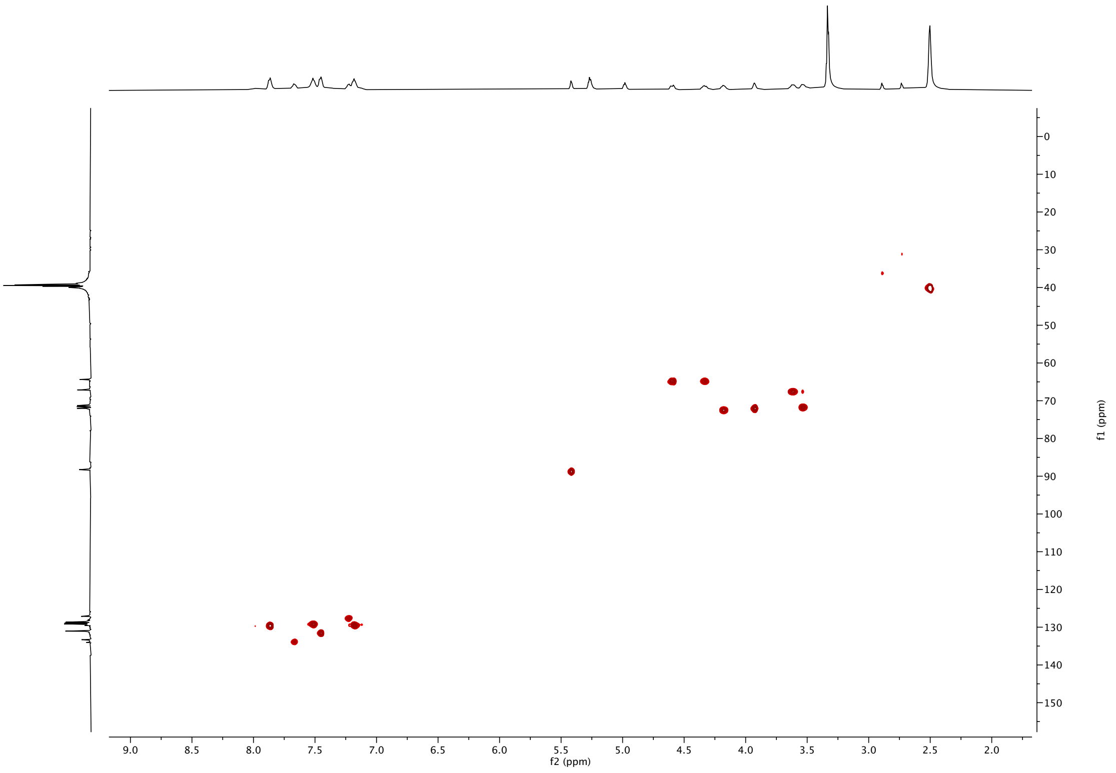

Phenyl 3,6-di-*O*-benzoyl-α-D-thiomannopyranoside (4)

^{1}H NMR (500MHz, DMSO)

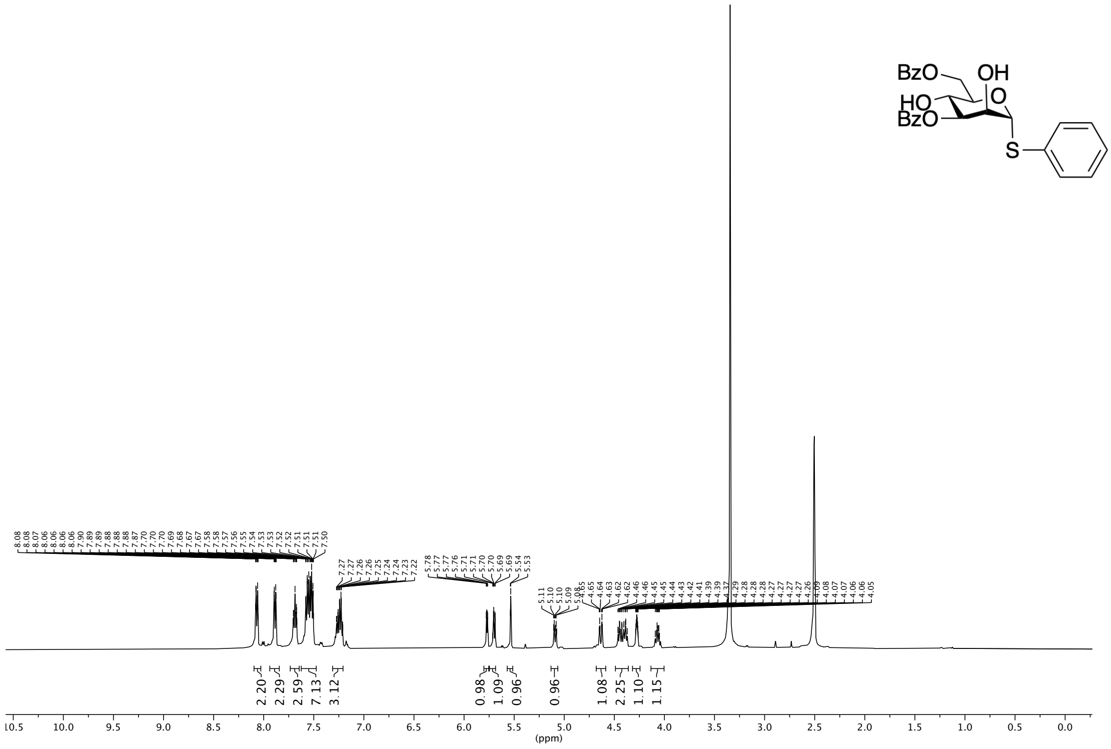

^{13}C NMR (126MHz, DMSO)

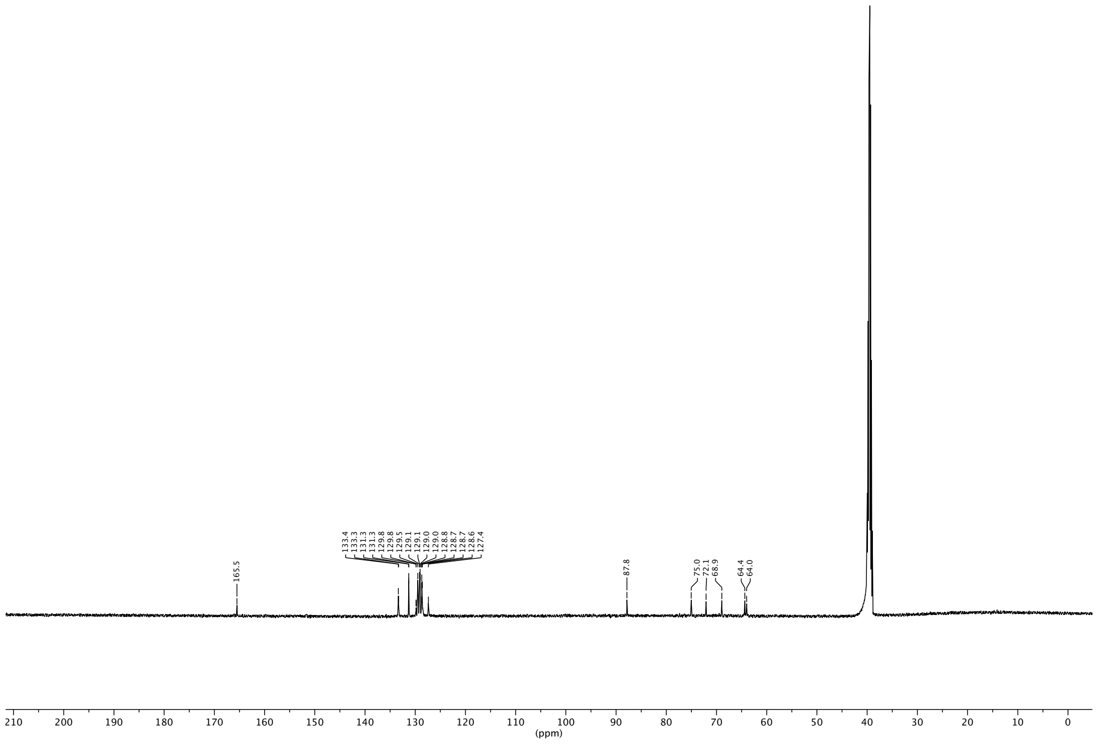

COSY ^{1}H NMR (500MHz, DMSO)

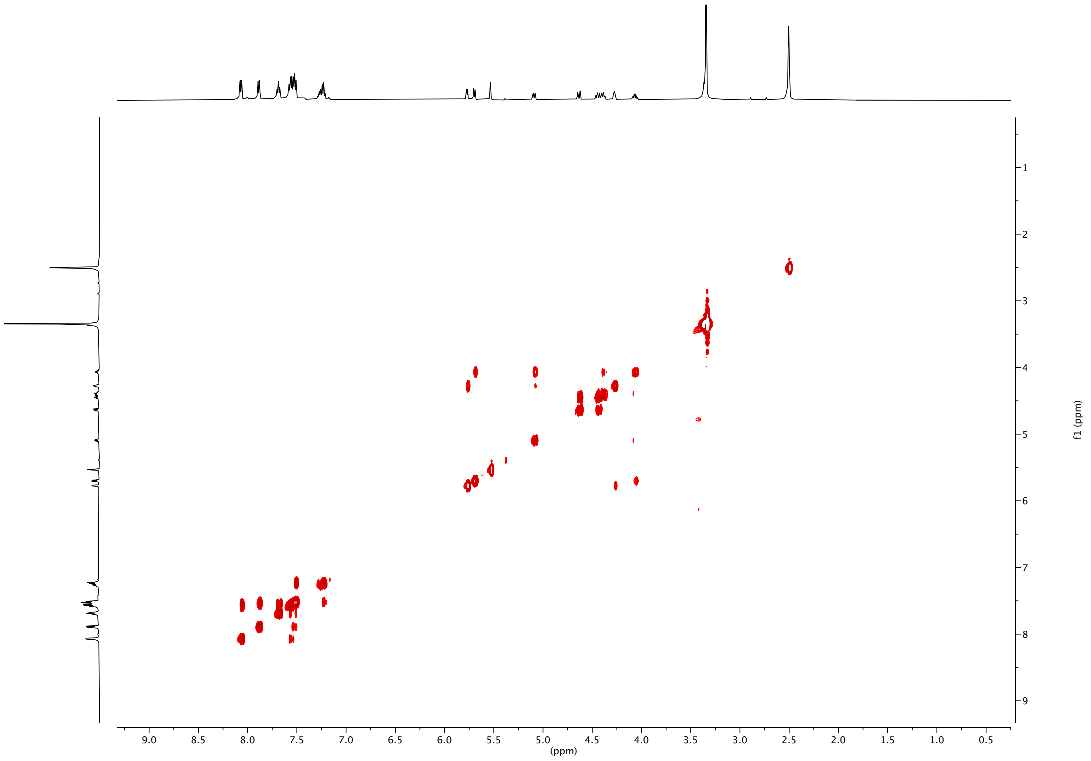

HSQC ^{1}H NMR (500MHz, DMSO), ^{13}C NMR (126MHz, DMSO)

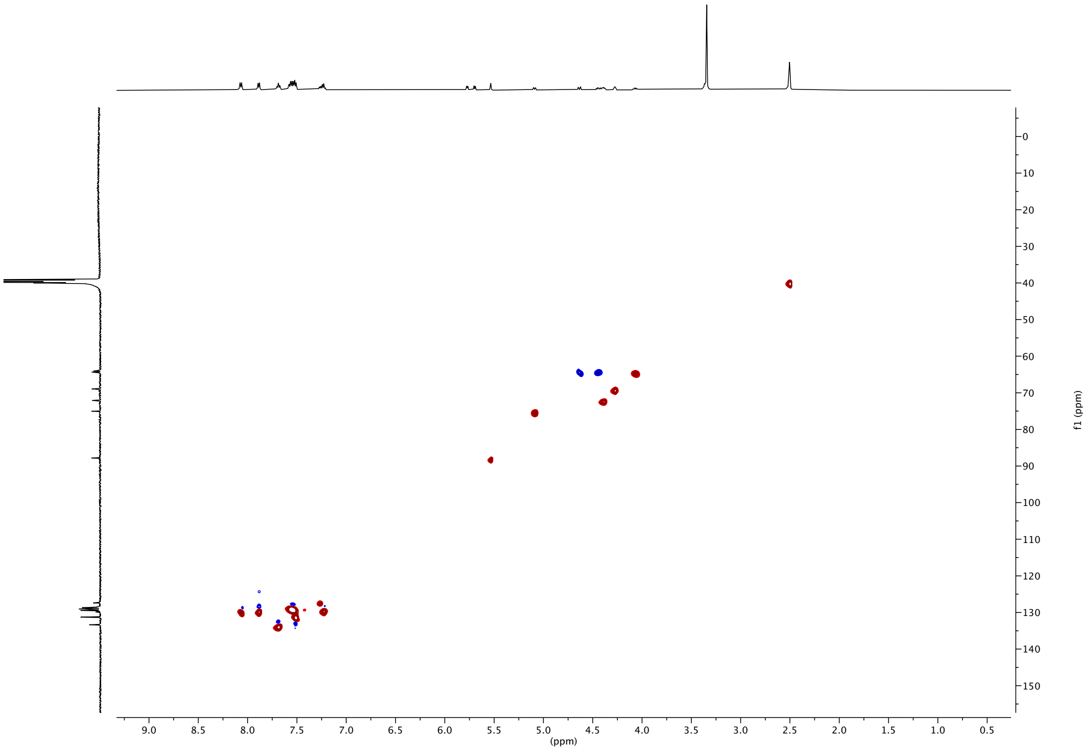

Phenyl 6-*O*-benzoyl-β-D-galactopyranoside (5)
^{1}H NMR (500MHz, DMSO)

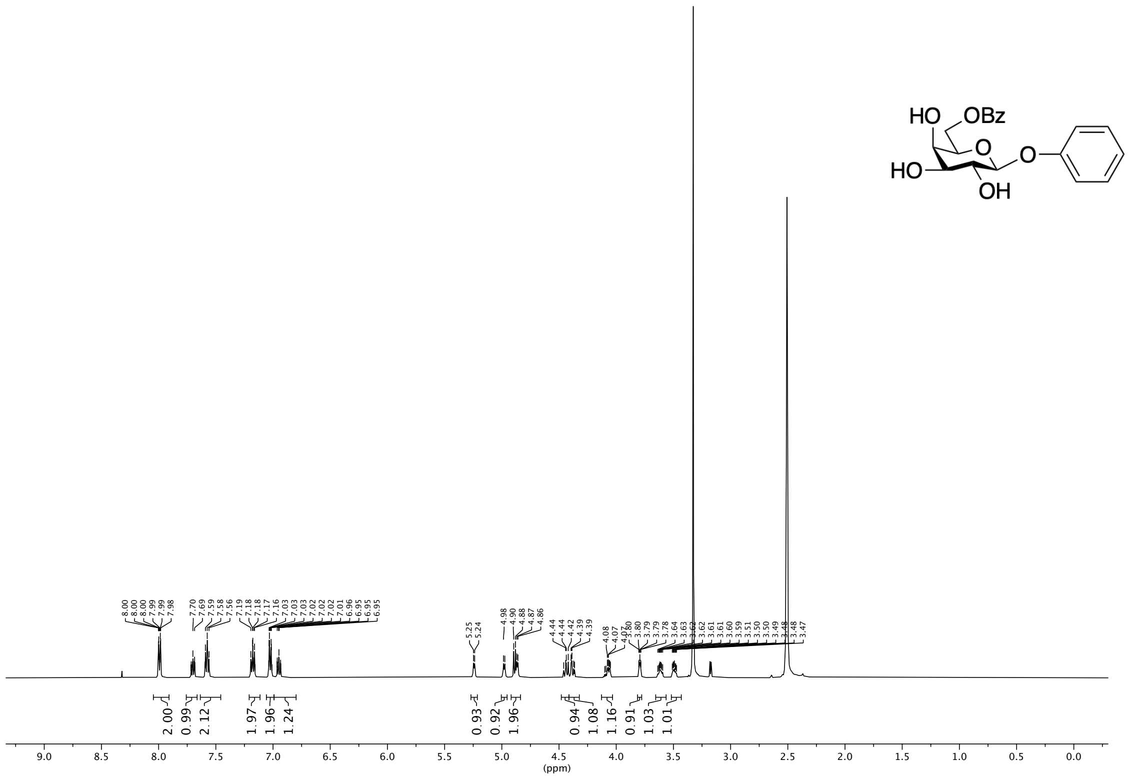

^{13}C NMR (126MHz, DMSO)

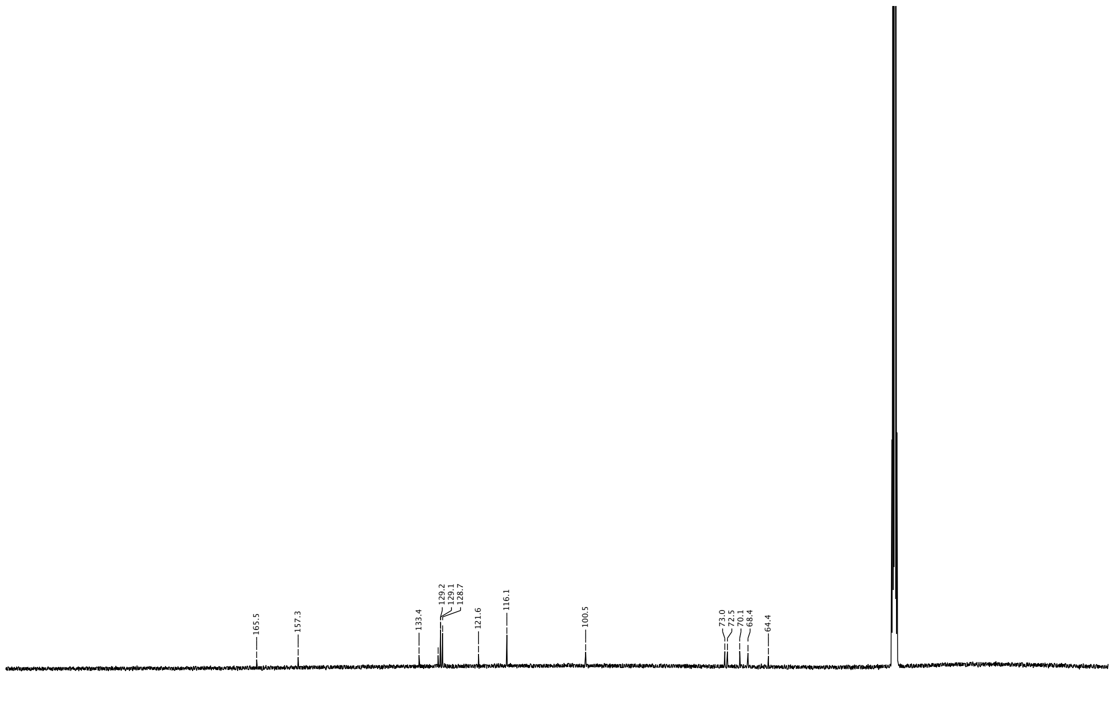

COSY ^{1}H NMR (500MHz, DMSO)

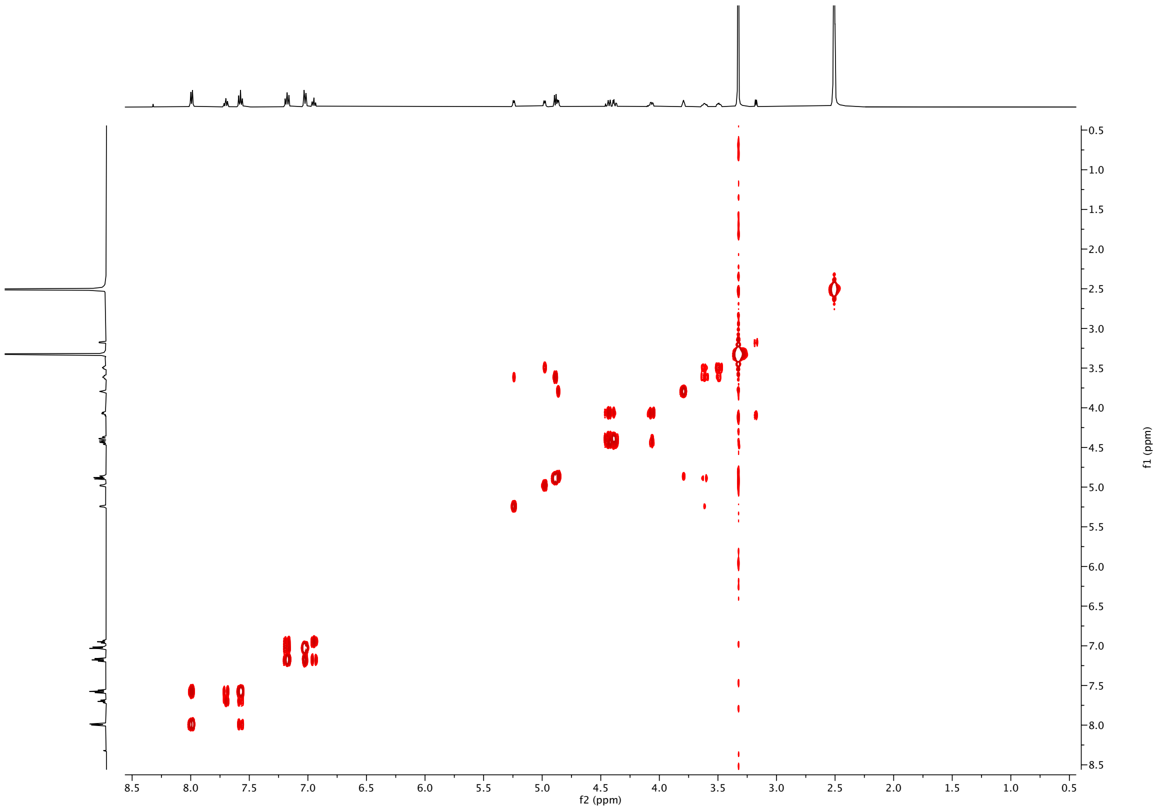

HSQC ^{1}H NMR (500MHz, DMSO), ^{13}C NMR (126MHz, DMSO)

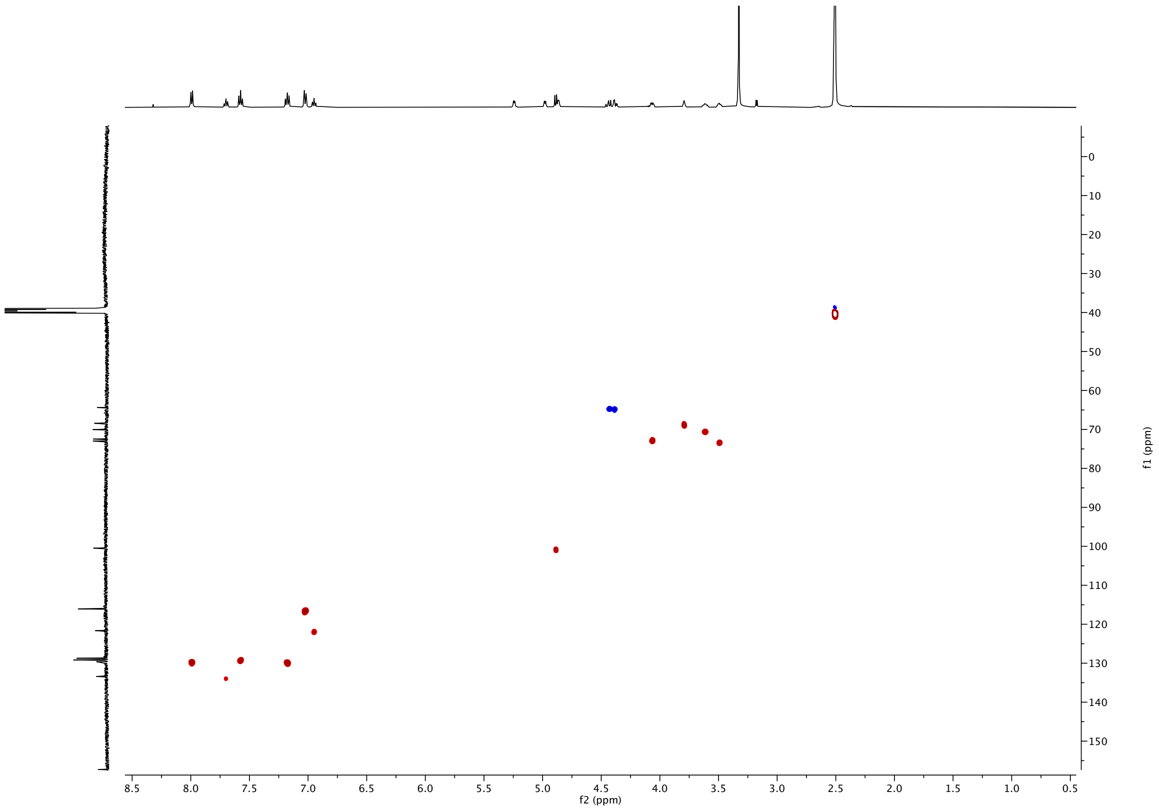

Phenyl 3,6-di-*O*-benzoyl-β-D-galactopyranoside (6)
^{1}H NMR (500MHz, DMSO)

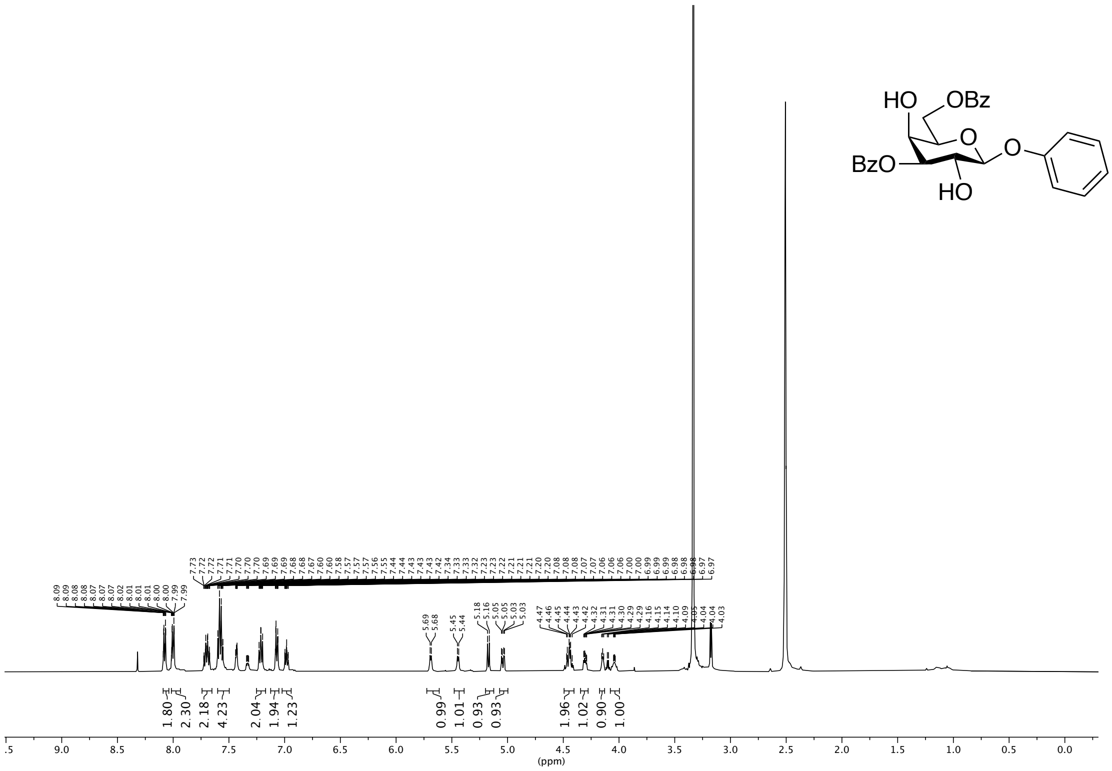

^{13}C NMR (126MHz, DMSO)

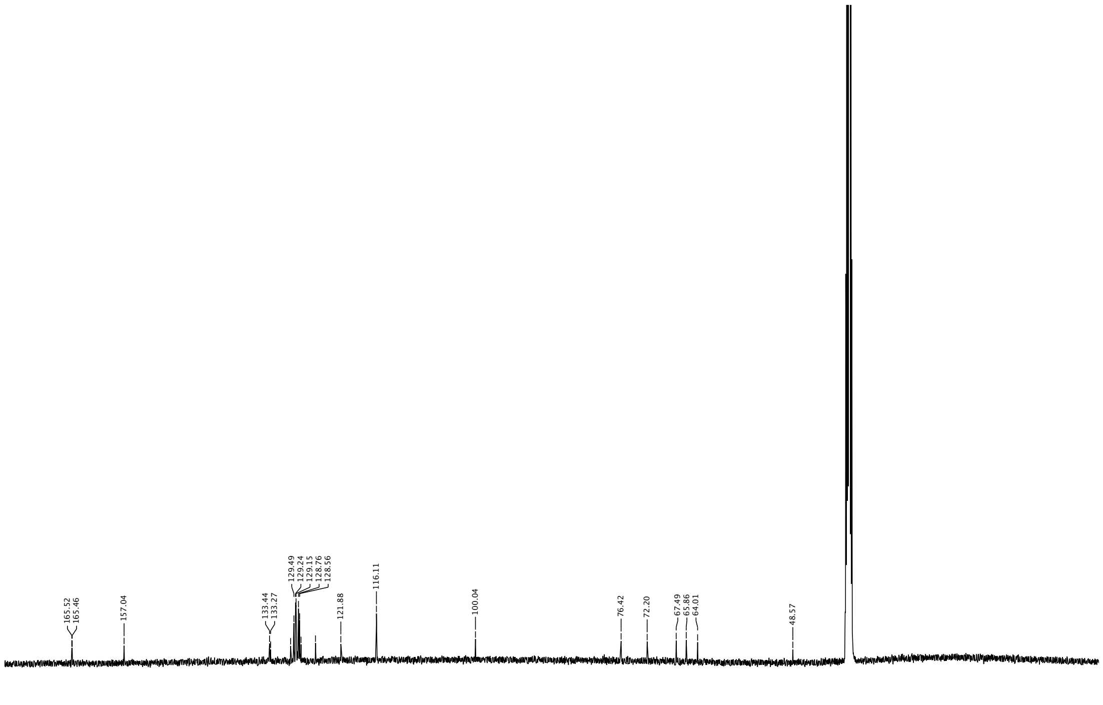

COSY ^{1}H NMR (500MHz, DMSO)

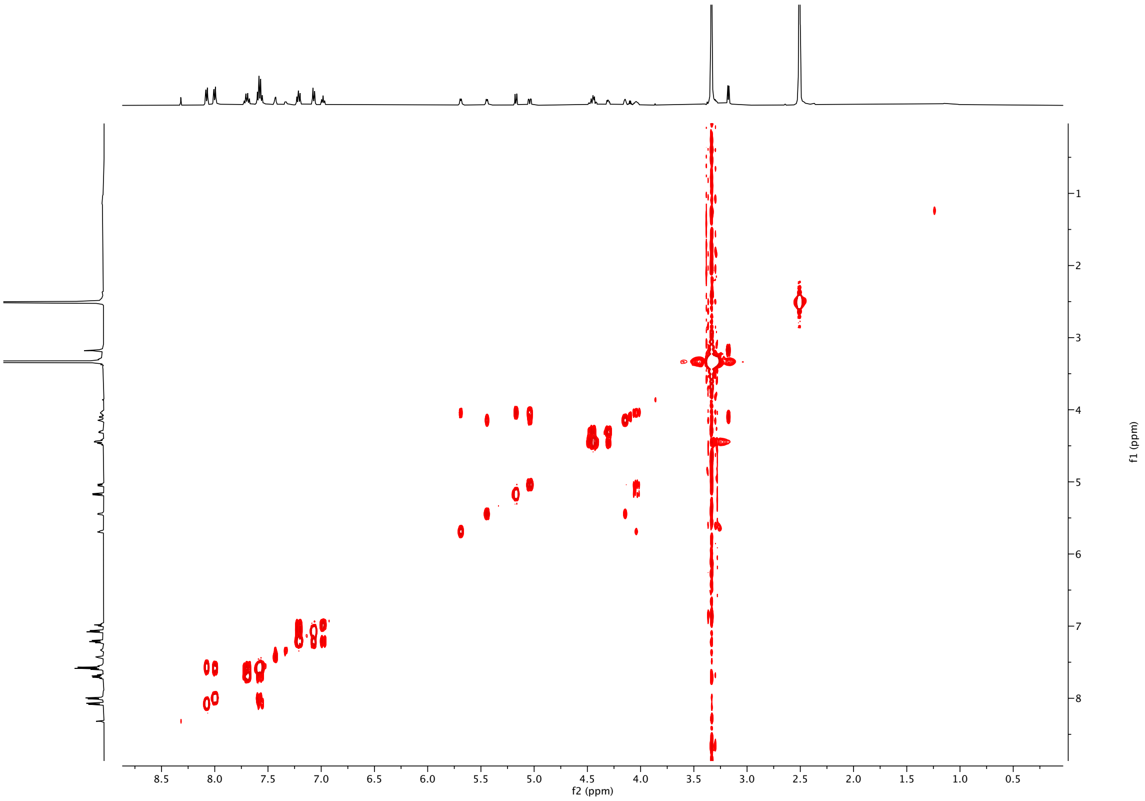

HSQC ^{1}H NMR (500MHz, DMSO), ^{13}C NMR (126MHz, DMSO)

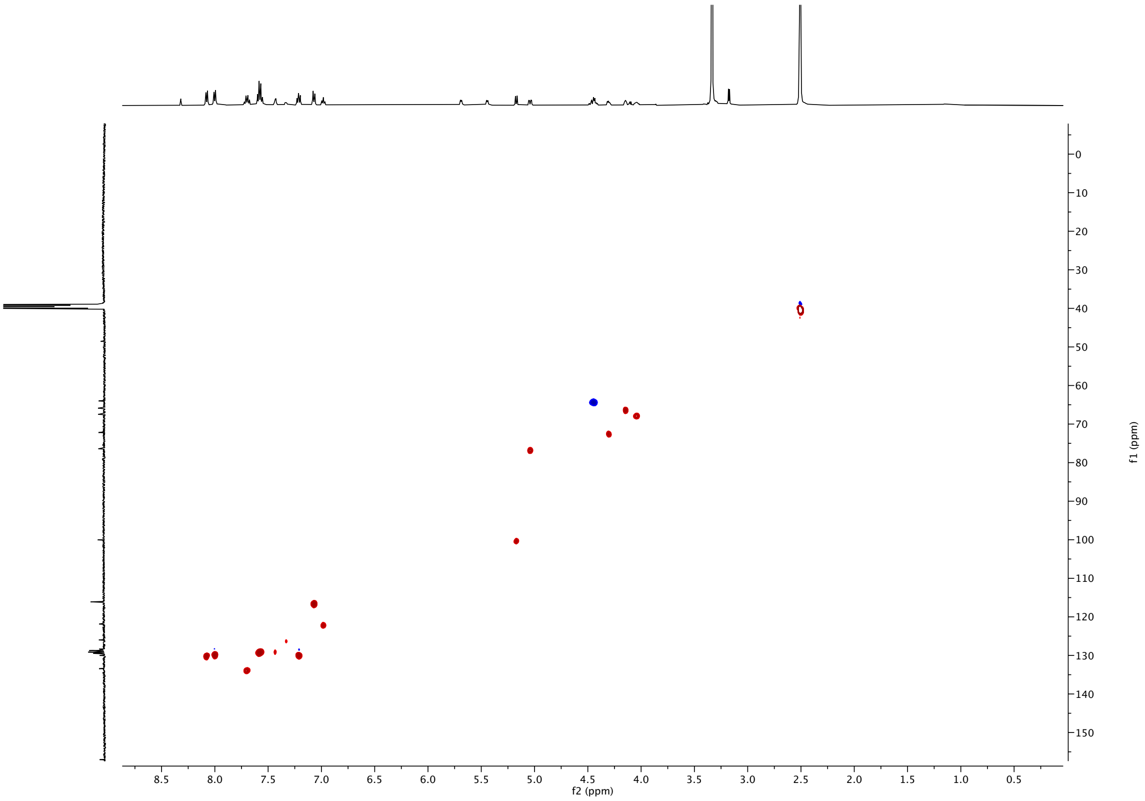

Phenyl 6-*O*-benzoyl-β-D-glucopyranoside CRUDE

^{1}H NMR (500MHz, DMSO)

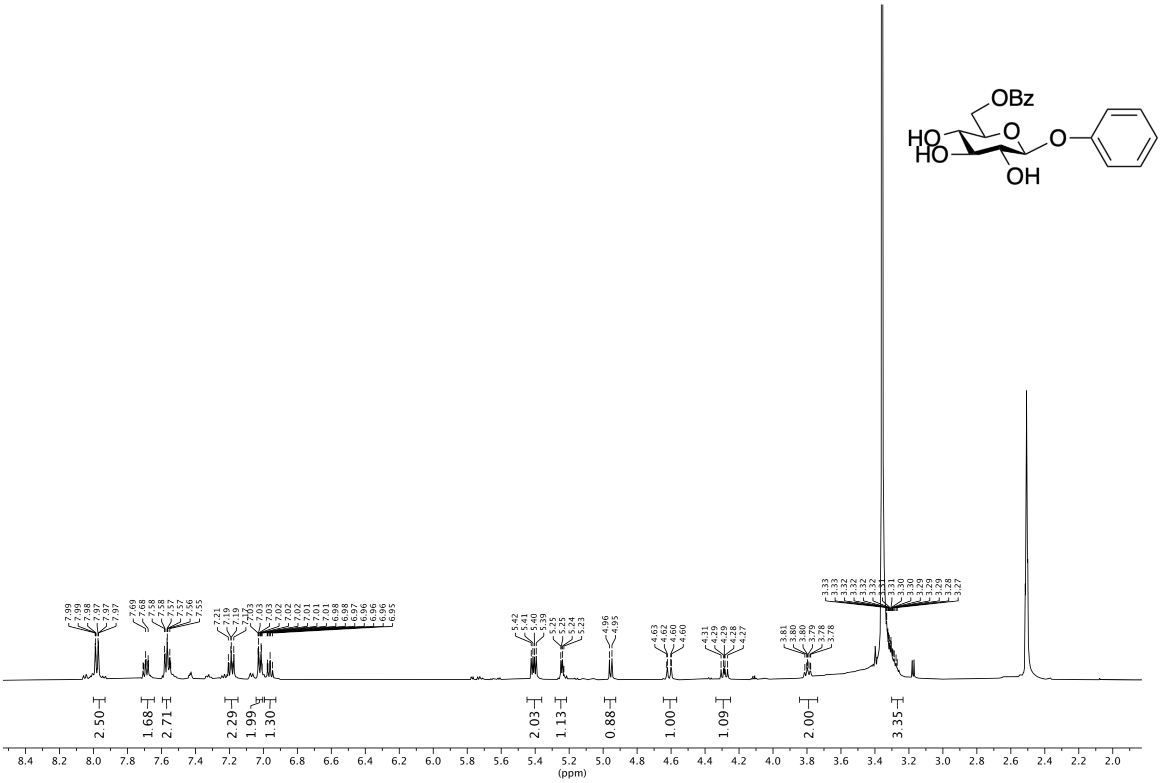

^{13}C NMR (126MHz, DMSO)

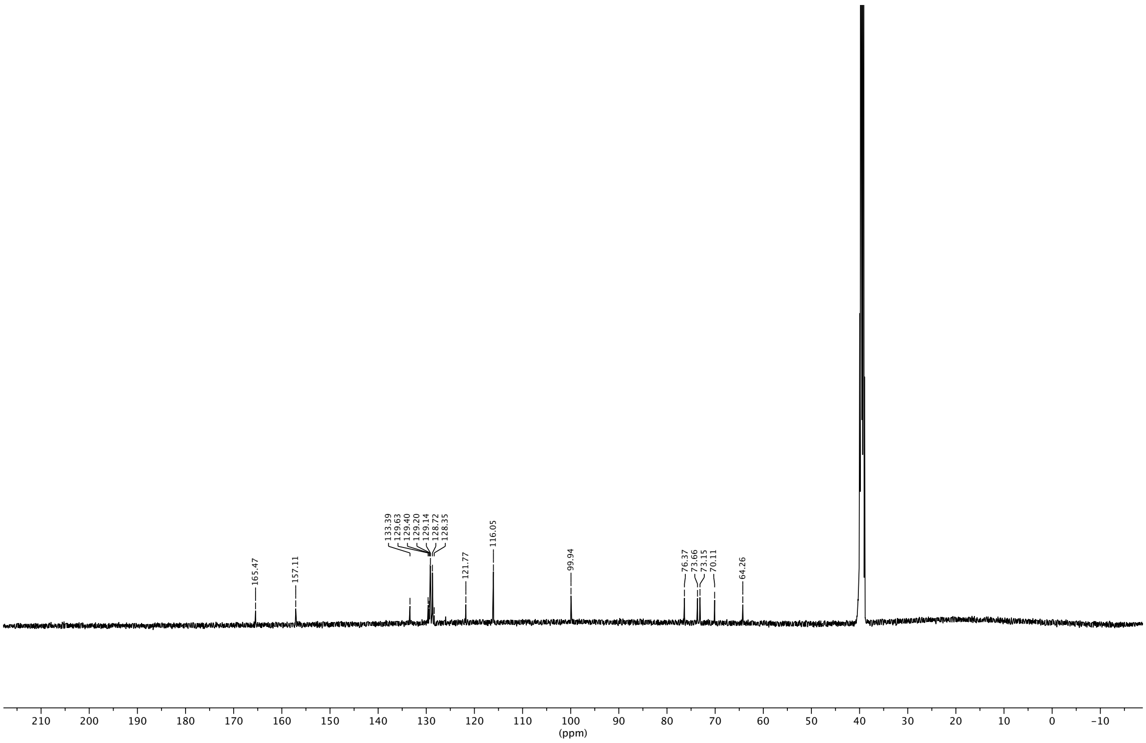

Phenyl 6-*O*-benzoyl-β-D-galactopyranoside CRUDE

^{1}H NMR (500MHz, DMSO)

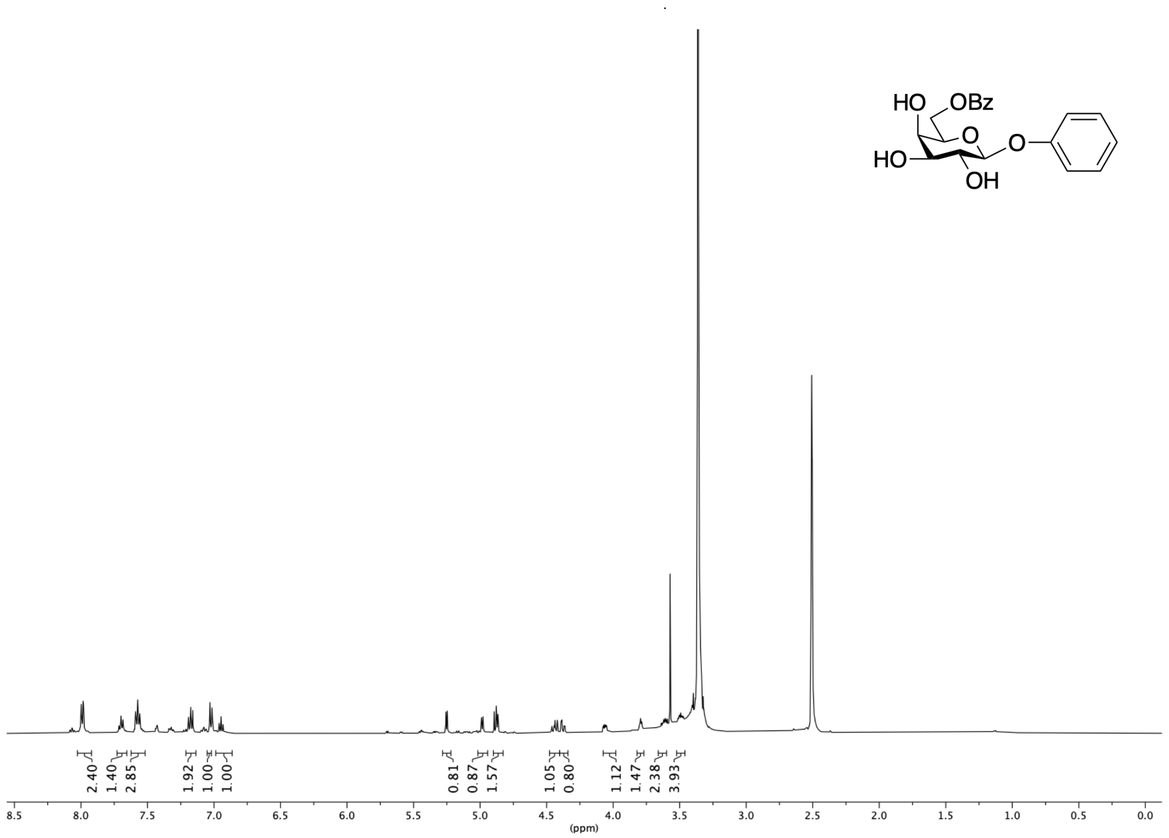

^{13}C NMR (126MHz, DMSO)

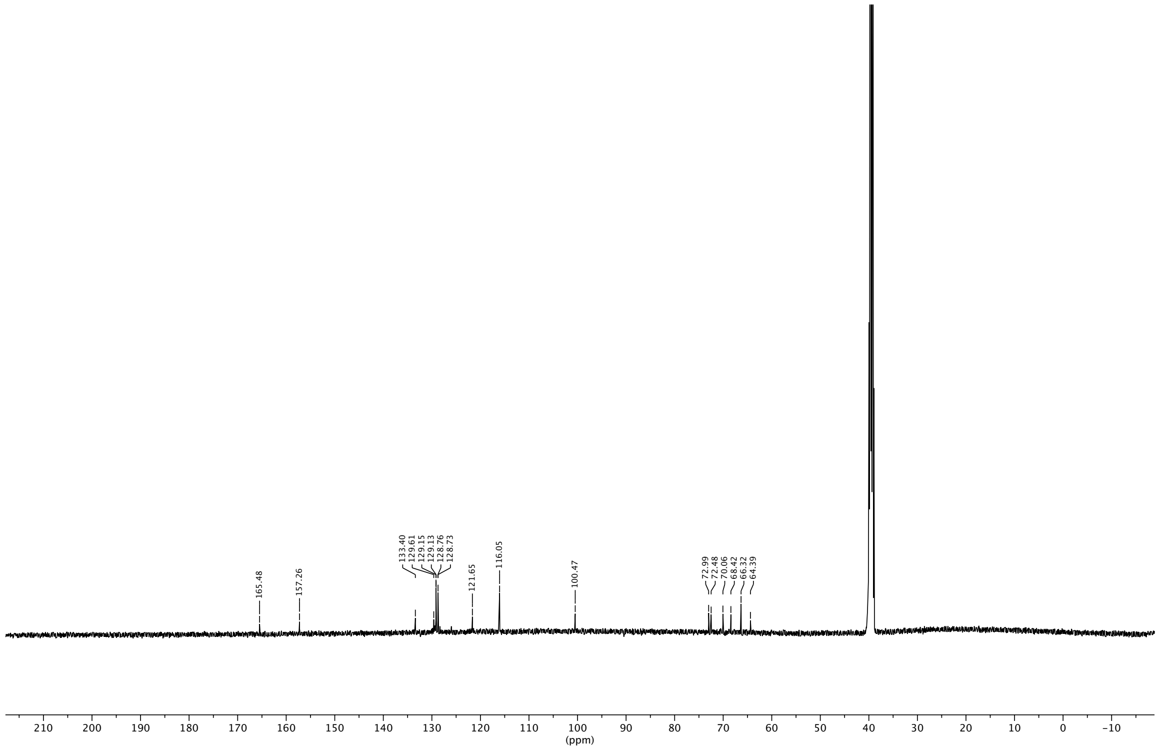

Results and Discussion

Benchmark study

The study has been performed utilizing reaction data for a direct arylation, which has been obtained by Doyle and coworkers. [1] The reaction and the reaction space are depicted in Scheme S1.

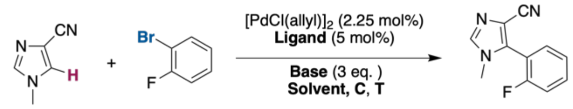

Variable:	Values:
Ligand	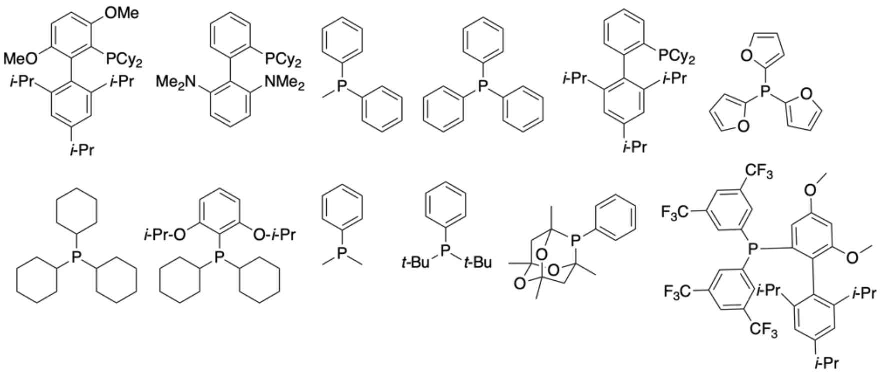
Base	KOAc, CsOAc, KOPiv, CsOPiv
Solvent	BuOAc, *p*-Xylene, BuCN, DMA
T (°C)	90, 105, 120
C (M)	0.057, 0.100, 0.153

Scheme S1. Reaction and reaction space used for the benchmark study. Dataset obtained by Doyle and co-workers.[1]

Setup

To carry out the benchmark study the script illustrated below was constructed:

Algorithm 1 Benchmark Script

```
for optimization in range(30) do
  for experiment in range(100) do
    Query ProcessOptimizer for proposed reaction conditions
    Get yield for queried reaction conditions from Doyle dataset
    Give yield from queried reaction conditions to ProcessOptimizer
  end for
end for
```

The script can query the optimizer for reaction conditions, get the yield obtained from these reaction conditions from the Doyle dataset, and tell the yield to the optimizer, which based on the yield propose a new experiment and so forth. This inner loop is run 100 times, corresponding to 100 experiments. The inner loop is looped over 30 times and the average is calculated. Each optimization run is initiated by 10 random sets of reaction conditions. This is done to average out statistical

uncertainties and make sure the values compared have some statistical significance. If only one optimization was run for each method, one would not be able to say with certainty if a method performs better because it is in fact superior or because it had a better starting point. Unless otherwise noted the default values or conditions of the ProcessOptimizer have been used, which, at the time the benchmark study was conducted, were:

Default ProcessOptimizer settings

Optimizer *(dimensions, base_estimator='gp', n_random_starts=None, n_initial_points=10, initial_point_generator='random', n_jobs=1, acq_func='gp_hedge', acq_optimizer='auto', random_state=None, model_queue_size=None, acq_func_kwargs=None, acq_optimizer_kwargs=None)*

As seen in Scheme S1**,** all the parameters in the reaction space are either categorical or have discrete values. Currently the ProcessOptimizer can handle categorical, continuous, and integer parameters. Three different approaches to describe the discrete parameters were therefore proposed:

1. Treat them as categorical parameters.
2. Treat them as continuous parameters and return the discrete value closest to the value proposed by the optimizer.
3. Keep them as discrete parameters using integers, with each integer corresponding to a discrete value.

All three methods were tested; the results are shown in Figure Sb1. The optimizations were performed using an extra trees base estimator and using the sampling setting to minimize the acquisition function.

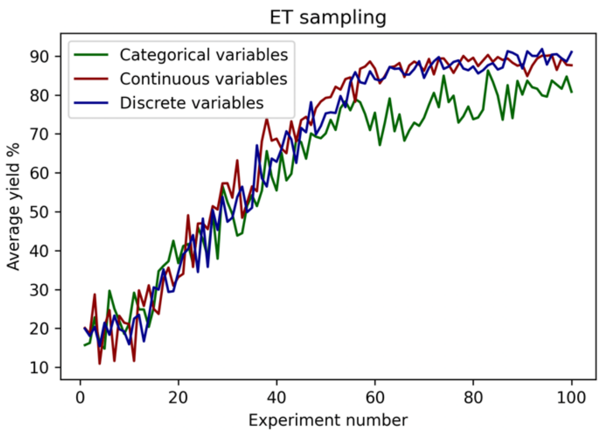

Figure Sb1. Average yield over 30 optimizations as a function of suggested experiment number, when using different approaches to describe the non-categorical parameters. An extra trees base estimator, sampling to minimize the acquisition function, and 10 initial points have been used.

It is seen that treating all parameters as categorical parameters gives the worst performance, whereas using continuous or discrete parameters give similar performances. The approach using integers as discrete parameters was chosen for the following studies.

Parameter tuning

Base estimator*:* The performance of different base estimators was also investigated. The acquisition function was set to "gp_hedge", which probabilistically choose between the LCB, EI and PI acquisition functions at each interaction. The result are displayed in Figure b2. Four different base estimators were tested: gradient boosted regression tree (GBRT), random forest (RF), extra trees (ET), and two variants of Gaussian process, one with sampling minimization and one using L-BFGS minimization. It is seen that all methods initially have a similar performance. After around 10 experiments the GBRT base estimator seems to perform best but afterwards the performance plateaus.

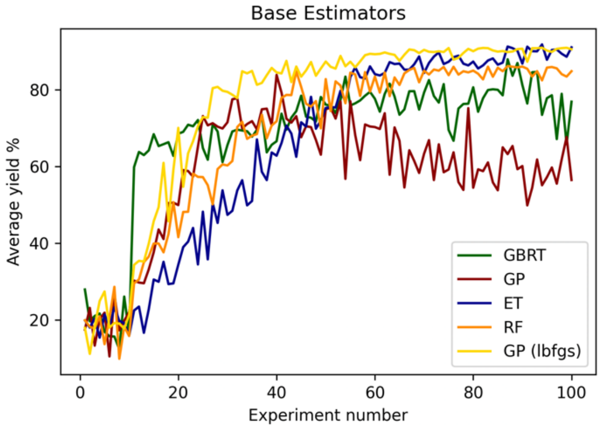

Figure S2: Average yield over 30 optimizations as a function of suggested experiment number, when using different base estimators and 10 initial points.

The methods using a GP base estimator and the RF base estimator seem to perform best overall. Though, the GP base estimator with L-BFGS minimization performs better than the GP base estimator with sampling minimization. L-BFGS minimization is not possible for the tree based base estimators.

Acquisition function: The effect of changing the acquisition function for different base estimators has also been investigated; Figure S3 to Figure Figure S5. In general, there is not a big difference in the performance of the acquisition functions. Though, when using a GP base estimator and L-BFGS minimization the gp hedge method (\ref{fig:acq_gp_L-BFGS}) seems to converge at a higher value. In general, it seems that the PI acquisition function gives smaller fluctuations, which might suggest that it is less explorative under the default conditions.

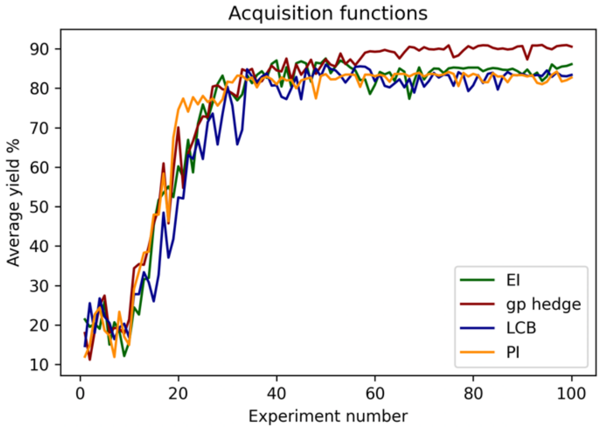

Figure S3. Average yield over 30 optimizations as a function of suggested experiment number when using a GP base estimator, L-BFGS minimization, and 10 initial points..

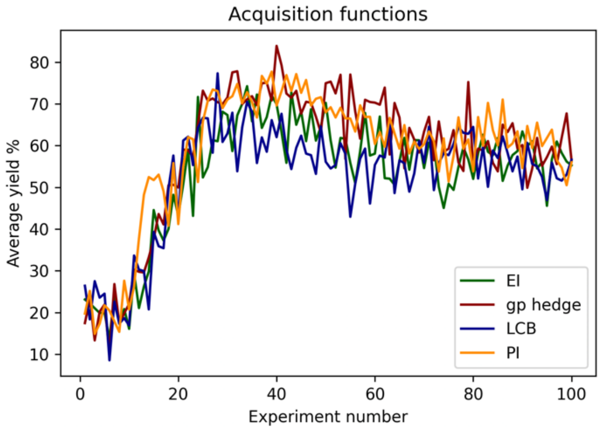

Figure Sb4: Average yield over 30 optimizations as a function of suggested experiment number when using a GP base estimator, sampling minimization, and 10 initial points

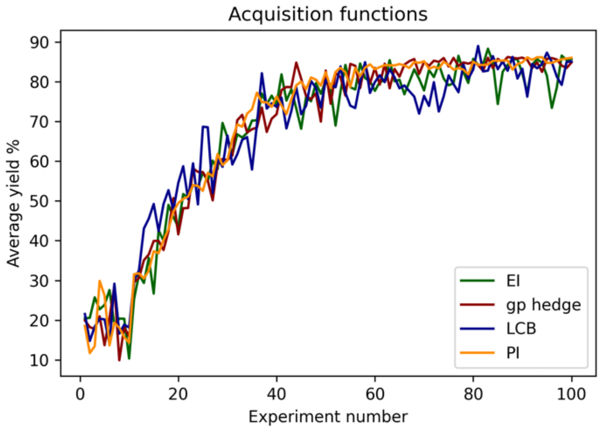

Figure S5: Average yield over 30 optimizations as a function of suggested experiment number when using a RF base estimator, sampling minimization, and 10 initial points.

Kernel: The default kernel, when using a GP base estimator, is a Matérn kernel with length scale bounds (lsb) of 0.00001 to 100000 and ν= 1.5. The effect of changing the hyperparameters of the Matérn kernel is shown in Figure S6. No great differences are observed, however, length scale bounds of 0.1-10 seem to give the highest observed maximum yields.

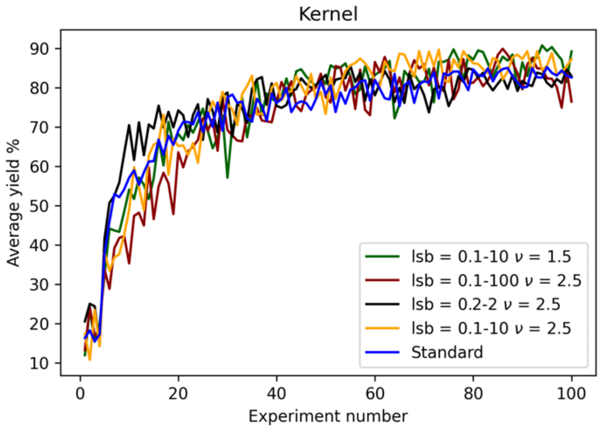

Figure S6: Average yield over 30 optimizations as a function of experiment number, a GP base estimator, a GP Hedge acquisition function, 4 initial points, and L-BFGS minimization have been used.

Robot setup

Photo from inside the robot

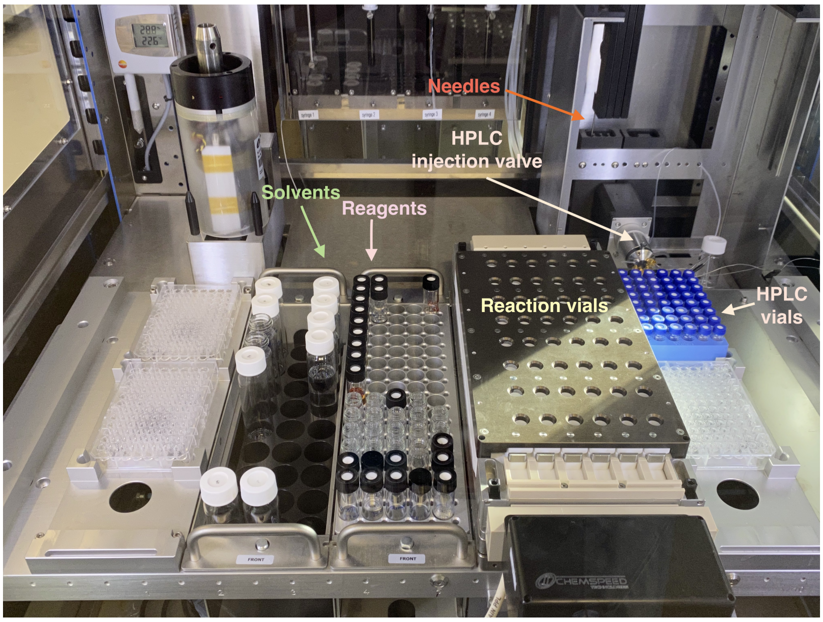

Figure S7: Picture showing the inside of the robot, with the placement of reagents and vials shown.

Flow diagram of script

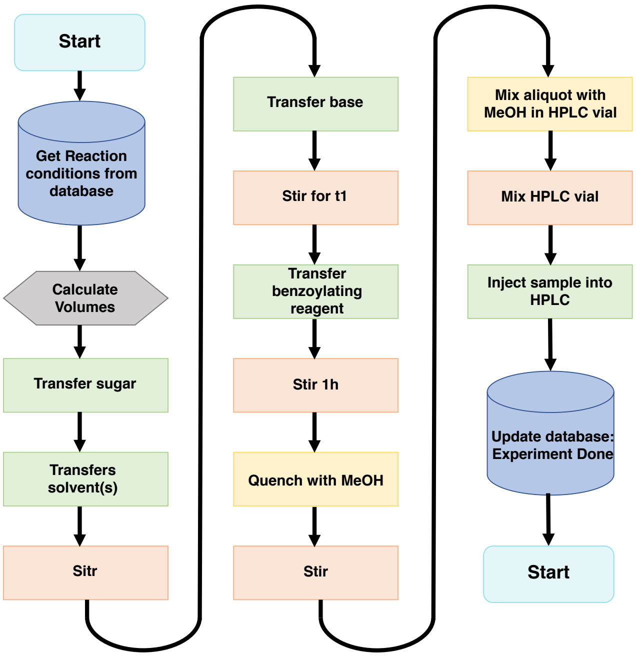

Figure S8: Flow diagram showing the experimental procedure performed by the robot.

Detailed description of quenching and HPLC

The reactions were quenched with methanol until a total volume of 6 mL was reached. Initially, insufficient mixing after quenching was observed using only the built-in stirring plate, when the 7 mL vials were filled to 6 mL. To obtain sufficient mixing of the quenched reaction mixture the vial was stirred for 2 minutes at 400 rpm, hereafter the mixture was “manually” mixed by the robot. The mixing was carried out by, first transferring 1 mL of the mixture from the top to the bottom of the vial two times, hereafter 1 mL from the bottom was transferred from the bottom to the top twice. After the mixing, the mixtures were stirred for an additional 5 minutes at 300 rpm. The approach was validated by recording a chromatogram after the mixing was performed by the robot. Hereafter, the quenched vial was removed and shaken vigorously by hand, and a second chromatogram was recorded. No change in peak integrals occurred, indicating full mixing was obtained during the automated mixing.

After quenching, the reaction mixture 0.15 mL of the quenched solution was further diluted with methanol (1.3 mL) in a Waters HPLC vial. As it is not possible to stir the HPLC vial rack, an approach like the one described above was used to obtain a homogenous mixture in the HPLC vials. It was found that mixing by transferring 0.5 mL of the mixture from the bottom of the vial to the top twice, was sufficient mixing i.e., no change in integrals after shaking the vials by hand.

Chemspeed Script

Robot script macros

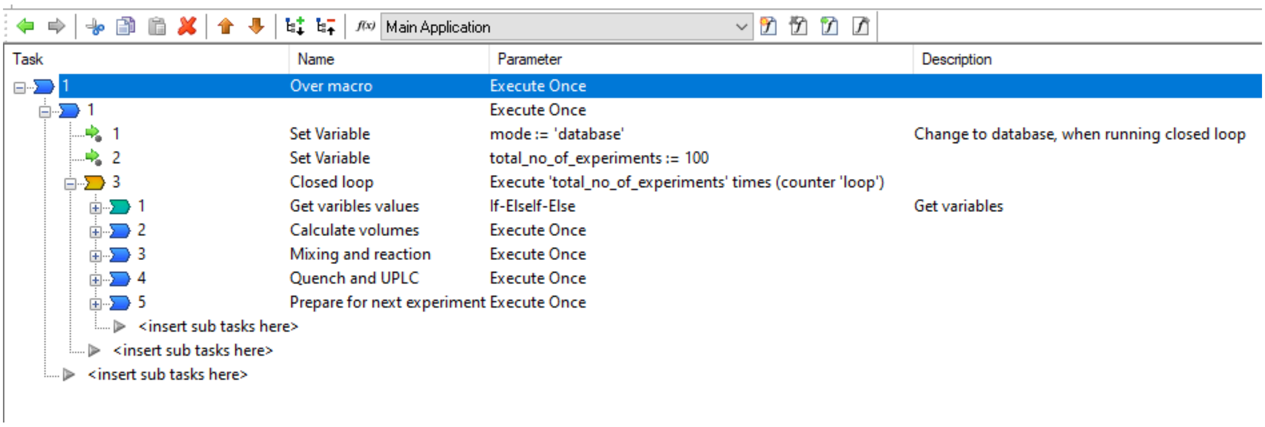

Robot script with unfolded macros

Task	Name	Parameter	Description
1	Over macro	Execute Once	
1		Execute Once	
1	Set Variable	mode := 'database'	Change to database, when running closed loop
2	Set Variable	total_no_of_experiments := 27	
3	Closed loop	Execute 'total_no_of_experiments' times (counter 'loop')	
1	Get varibles values	If-Elself-Else	Get variables
1	If	Execute If 'mode = 'simulation''	Values for simulation
1	Set Variable	base := 8	
2	Set Variable	eq_base := 4	
3	Set Variable	benzoylating_reagent := 1	
4	Set Variable	eq_benzoylating_reagent := 2	
5	Set Variable	solvent := 'dmf_thf'	
6	Set Variable	t1 := 10	
7	Set Variable	concentration := 0.1	
<insert sub tasks here>			
2	Else If	Execute Else If 'mode = 'database''	Get values from database
1	Database Task	Database Operation 'Read' on device Database #1	
<insert sub tasks here>			
3	Else	Execute Else	
2	Calculate volumes	Execute Once	
1	determine base and volume	Execute Once	
1	Create arrays	Execute Once	
1	Base density array	Execute Once	
2	Base MW array	Execute Once	
<insert sub tasks here>			
2	Set Variable	active_base := all_bases[base - 1]	
3	Set Variable	vol_base := ((eq_base * n_hemi * mw_array[base-1]) / density_arra	
<insert sub tasks here>			
2	determine benzoylating reag	Execute Once	
1	Macro Task	Execute If 'benzoylating_reagent = 1'	
1	Set Variable	bz_reagent := BzCl	
2	Set Variable	vol_bzreagent := ((n_hemi*140.57* eq_benzoylating_reagent)/1.21),	
<insert sub tasks here>			
2	Macro Task	Execute If 'benzoylating_reagent = 2'	
1	Set Variable	bz_reagent := BzAnhydride	
2	Set Variable	vol_bzreagent := (n_hemi/4) /1000 * eq_benzoylating_reagent	
<insert sub tasks here>			
<insert sub tasks here>			
3	Determine solvent volume	Execute Once	
1	Set Variable	total_volume := (n_hemi / concentration) / 1000	
2	Set Variable	vol_sol := total_volume - vol_base - vol_bzreagent - vol_sugar_2M	
<insert sub tasks here>			
4	Volume check	Execute If 'vol_bzreagent + vol_base + vol_sol + vol_sugar_2M > 5'	
1	Show Dialog	OK/Stop-Dialog: 'Volumes exceed max volume'	
<insert sub tasks here>			
<insert sub tasks here>			

3	Mixing and reaction	Execute Once
1	Set Variable	active_vial := all_vials[vial_number]
2	Set Drawer Valve	Set Reactionblock state to Open Under Inert Gas on zone variable '
3	Transfer Volumetrically	Transfer liquid from sugar to variable 'active_vial' with Needle Heac
4	Transfer Volumetrically	Transfer liquid from VP E1 methanol to Waste 1 with Needle Head ;Flush with methanol before washing needle
5	Choose and add solvent	If-Elself-Else
6	Set Drawer Valve	Set Reactionblock state to Closed Under Inert Gas on zone variable
7	Stir	Agitation ON on zone reaction_vial
8	Wait	Waiting for 2:00 minutes
9	Stir	Agitation OFF on zone reaction_vial
10	Set Drawer Valve	Set Reactionblock state to Open Under Inert Gas on zone variable '
11	Transfer Volumetrically	Transfer liquid from variable 'active_base' to variable 'active_vial' w
12	Set Timer	Set the timer 't1'
13	Set Drawer Valve	Set Reactionblock state to Closed Under Inert Gas on zone variable
14	Stir	Agitation ON on zone reaction_vial
15	Wait	Waiting for 't1' after timer 't1'
16	Stir	Agitation OFF on zone reaction_vial
17	Set Drawer Valve	Set Reactionblock state to Open Under Inert Gas on zone variable '
18	Transfer Volumetrically	Transfer liquid from variable 'bz_reagent' to variable 'active_vial' wi
19	Set Drawer Valve	Set Reactionblock state to Closed Under Inert Gas on zone variable
20	Stir	Agitation ON on zone reaction_vial
21	Wait	Waiting for 't2'
22	Stir	Agitation OFF on zone variable 'active_vial'
<insert sub tasks here>		
4	Quench and UPLC	Execute Once
1	Set Variable	vol_meoh := end_volume - total_volume
2	Set Variable	active_uplc_vial := all_uplc_vials[vial_number]
3	Set Drawer Valve	Set Reactionblock state to Open Under Inert Gas on zone variable '
4	Transfer Volumetrically	Transfer liquid from VP E1 methanol to variable 'active_vial' with N
5	Set Drawer Valve	Set Reactionblock state to Closed Under Inert Gas on zone variable
6	Stirring and mixing	Execute Once
1	Stir	Agitation ON on zone variable 'active_vial'
2	Wait	Waiting for 2:00 minutes
3	Stir	Agitation OFF on zone variable 'active_vial'
4	Set Drawer Valve	Set Reactionblock state to Open Under Inert Gas on zone variable '
5	Transfer Volumetrically	Transfer liquid from variable 'active_vial' to variable 'active_vial' wit
6	Transfer Volumetrically	Transfer liquid from variable 'active_vial' to variable 'active_vial' wit
7	Transfer Volumetrically	Transfer liquid from variable 'active_vial' to variable 'active_vial' wit
8	Transfer Volumetrically	Transfer liquid from variable 'active_vial' to variable 'active_vial' wit
9	Set Drawer Valve	Set Reactionblock state to Closed Under Inert Gas on zone variable
10	Stir	Agitation ON on zone variable 'active_vial'
11	Wait	Waiting for 5:00 minutes
12	Stir	Agitation OFF on zone variable 'active_vial'
13	Set Drawer Valve	Set Reactionblock state to Open Under Inert Gas on zone variable '
<insert sub tasks here>		
7	Set Drawer Valve	Set Reactionblock state to Open Under Inert Gas on zone variable '
8	Transfer Volumetrically	Transfer liquid from variable 'active_vial' to variable 'active_uplc_vi
9	Transfer Volumetrically	Transfer liquid from meoh to variable 'active_uplc_vial' with Needle

Task	Name	Parameter	Description
3	Stir	Agitation OFF on zone variable 'active_vial'	
4	Set Drawer Valve	Set Reactionblock state to Open Under Inert Gas on zone variable 'a	
5	Transfer Volumetrically	Transfer liquid from variable 'active_vial' to variable 'active_vial' wit	
6	Transfer Volumetrically	Transfer liquid from variable 'active_vial' to variable 'active_vial' wit	
7	Transfer Volumetrically	Transfer liquid from variable 'active_vial' to variable 'active_vial' wit	
8	Transfer Volumetrically	Transfer liquid from variable 'active_vial' to variable 'active_vial' wit	
9	Set Drawer Valve	Set Reactionblock state to Closed Under Inert Gas on zone variable	
10	Stir	Agitation ON on zone variable 'active_vial'	
11	Wait	Waiting for 5:00 minutes	
12	Stir	Agitation OFF on zone variable 'active_vial'	
13	Set Drawer Valve	Set Reactionblock state to Open Under Inert Gas on zone variable 'a	
<insert sub tasks here>			
7	Set Drawer Valve	Set Reactionblock state to Open Under Inert Gas on zone variable 'a	
8	Transfer Volumetrically	Transfer liquid from variable 'active_vial' to variable 'active_uplc_via	
9	Transfer Volumetrically	Transfer liquid from meoh to variable 'active_uplc_vial' with Needle	
10	mix hplc vial	Execute Once	
1	Transfer Volumetrically	Transfer liquid from variable 'active_uplc_vial' to variable 'active_up	
2	Transfer Volumetrically	Transfer liquid from variable 'active_uplc_vial' to variable 'active_up	
<insert sub tasks here>			
11	Set Drawer Valve	Set Reactionblock state to Closed Under Inert Gas on zone variable	
12	Run UPLC	Execute Once	
1	HPLCInject	Execute Once	
1	Set Variable	ExperimentName := experiment_id	Change to variable describing SampleID
2	Set Variable	SampleZone := active_uplc_vial	
3	Macro Task	Execute While 'QueueStatus <> 'Idle''	Checks queue status until Idle
1	Import CSV	import row '1' from file 'status.csv''.	Check Status
2	Wait	Waiting for 5 seconds	
<insert sub tasks here>			
4	Macro Task	Execute If 'QueueStatus = 'Idle''	Inject to UPLC if idle
1	Execute Function	UPLC report	
2	Set Variable	QueueStatus := 'Busy'	Makes sure Idle queue loop is not skipped
<insert sub tasks here>			
<insert sub tasks here>			
<insert sub tasks here>			
<insert sub tasks here>			
5	Prepare for next experiment	Execute Once	
1	Set Variable	experiment_no := experiment_no +1	
2	Database Task	Database Operation 'Update' on device Database #1	
3	Database Task	Database Operation 'Create' on device Database #1	
4	Set Variable	vial_number := vial_number + 1	
5	Macro Task	Execute If 'vial_number > 47'	stops loop when reaction vial has to be changed
1	Show Dialog	OK/Stop-Dialog: ' Refill reaction vials and uplc vials and press OK '	
2	Set Variable	vial_number := 0	
<insert sub tasks here>			
<insert sub tasks here>			
<insert sub tasks here>			
<insert sub tasks here>			
<insert sub tasks here>			

Standard curves

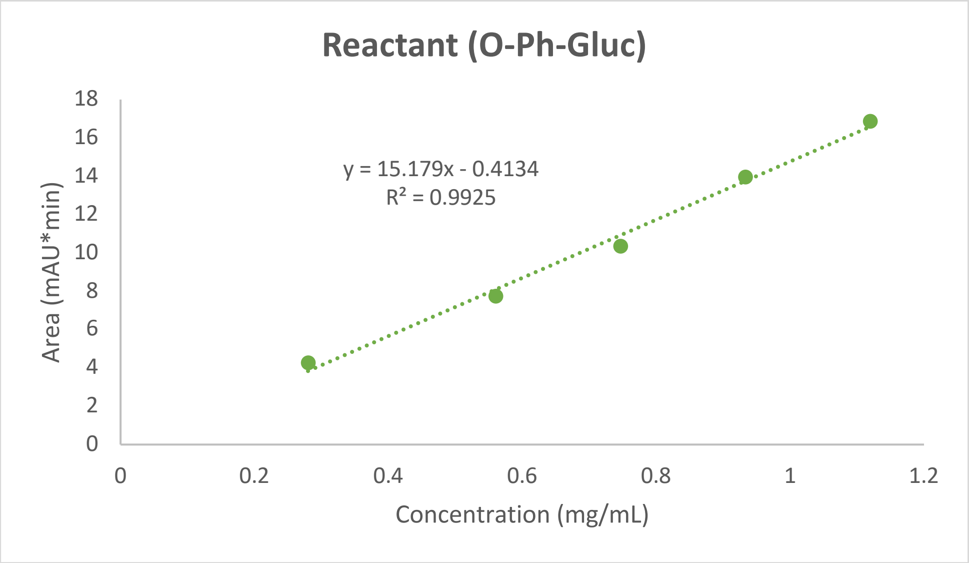

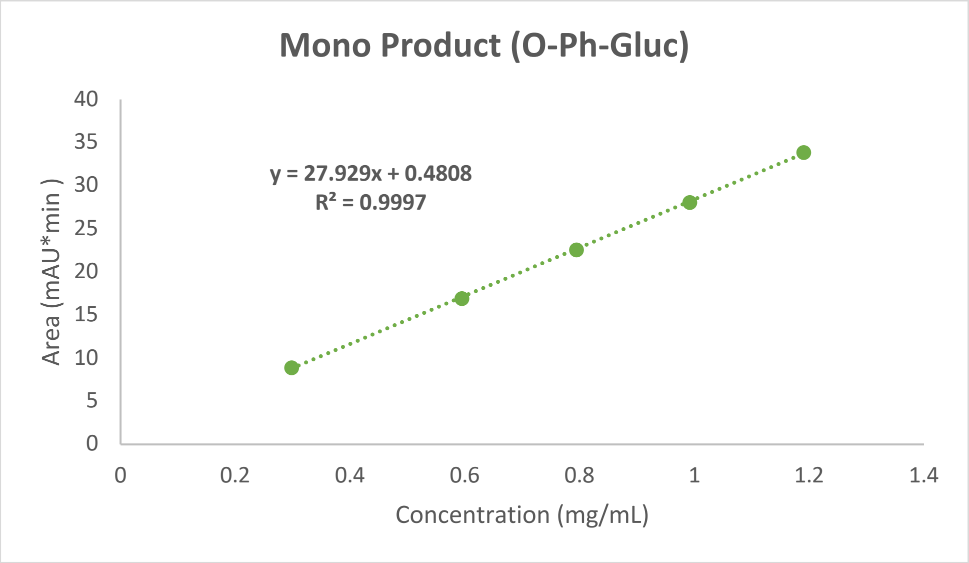

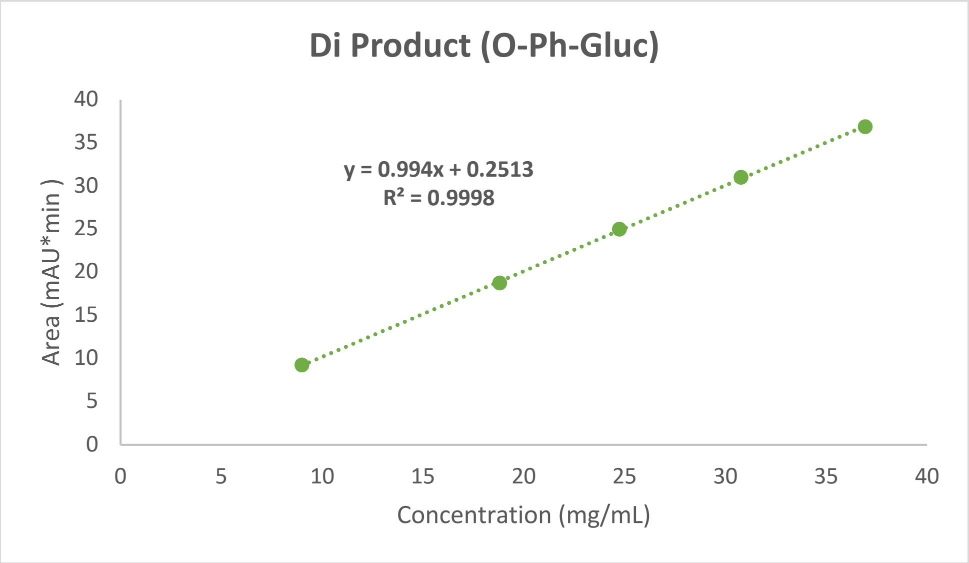

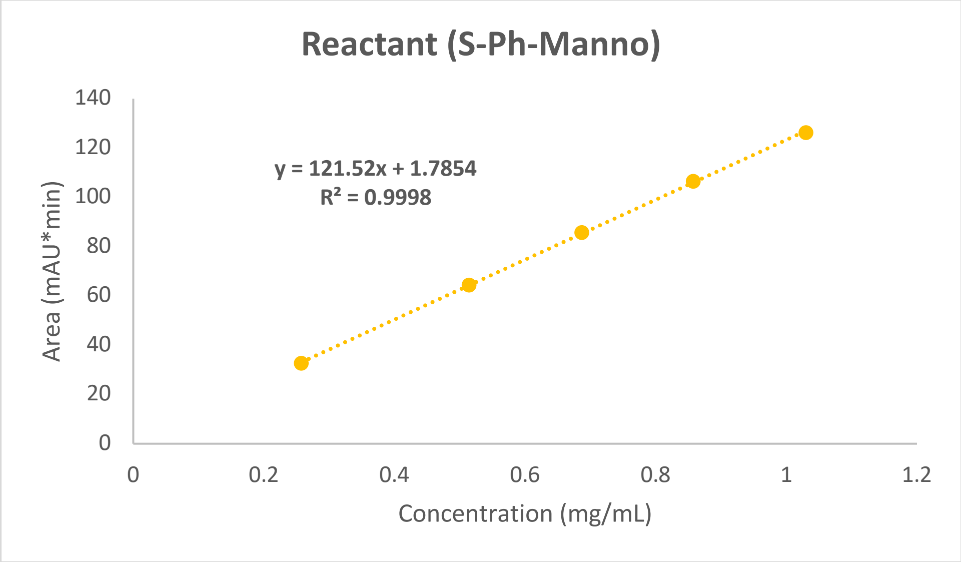
Reactant (S-Ph-Manno)
y = 121.52x + 1.7854
R² = 0.9998
Area (mAU*min)
140
120
100
80
60
40
20
0
0
0.2
0.4
0.6
0.8
1
1.2
Concentration (mg/mL)

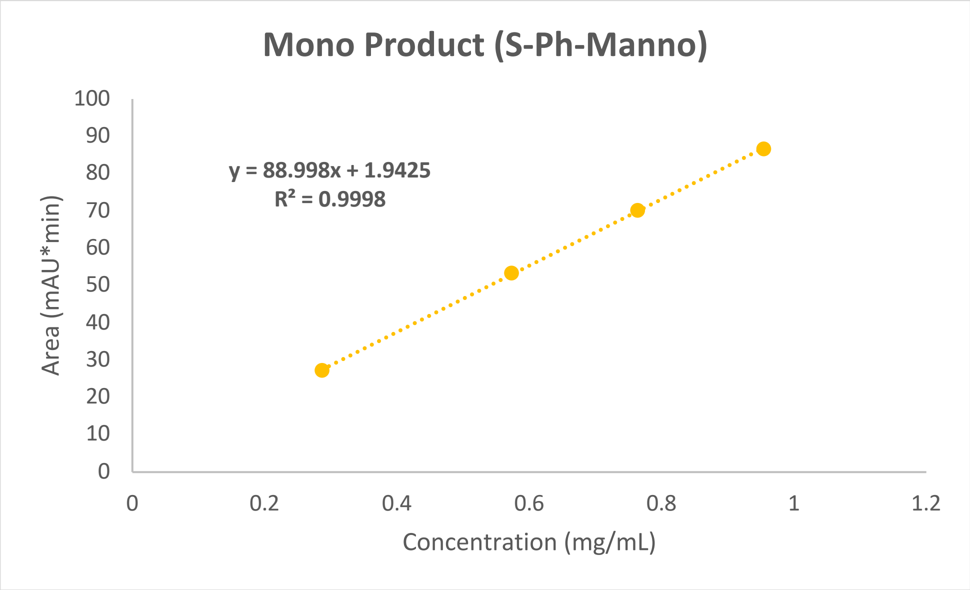
Mono Product (S-Ph-Manno)
y = 88.998x + 1.9425
R² = 0.9998
Area (mAU*min)
100
90
80
70
60
50
40
30
20
10
0
0
0.2
0.4
0.6
0.8
1
1.2
Concentration (mg/mL)

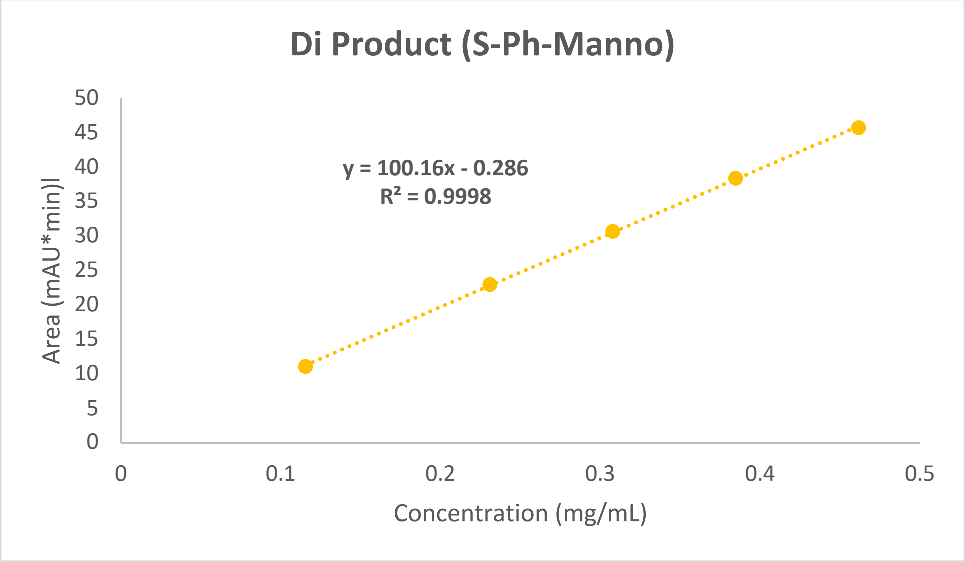
Di Product (S-Ph-Manno)
y = 100.16x - 0.286
R² = 0.9998
Area (mAU*min)l
50
45
40
35
30
25
20
15
10
5
0
0
0.1
0.2
0.3
0.4
0.5
Concentration (mg/mL)

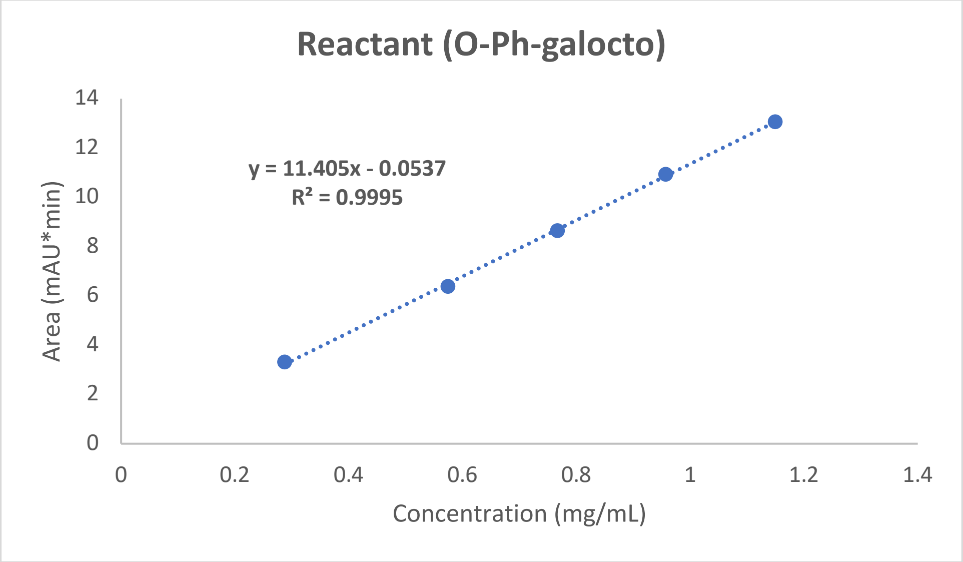

Reactant (O-Ph-galocto)
y = 11.405x - 0.0537
R² = 0.9995
Area (mAU*min)
Concentration (mg/mL)
0
2
4
6
8
10
12
14
0
0.2
0.4
0.6
0.8
1
1.2
1.4

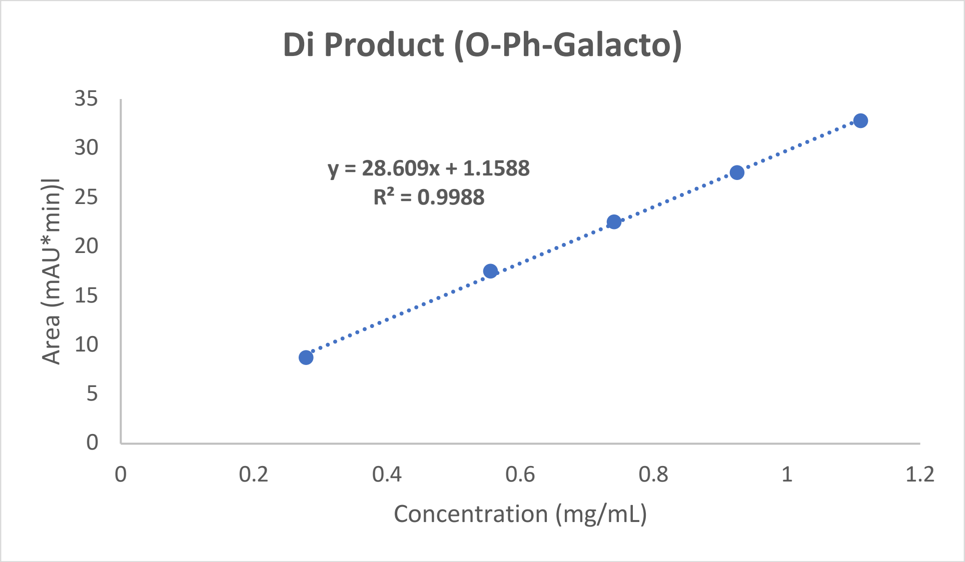

Di Product (O-Ph-Galacto)
y = 28.609x + 1.1588
R² = 0.9988
Area (mAU*min)l
Concentration (mg/mL)
0
5
10
15
20
25
30
35
0
0.2
0.4
0.6
0.8
1
1.2

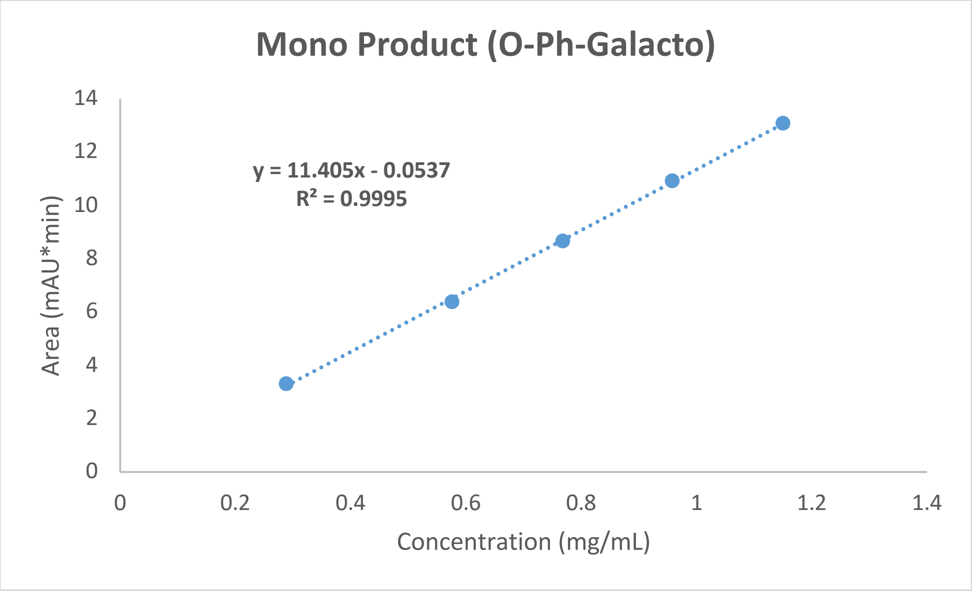

Mono Product (O-Ph-Galacto)
y = 11.405x - 0.0537
R² = 0.9995
Area (mAU*min)
Concentration (mg/mL)
0
2
4
6
8
10
12
14
0
0.2
0.4
0.6
0.8
1
1.2
1.4

Example of HPLC chromatogram

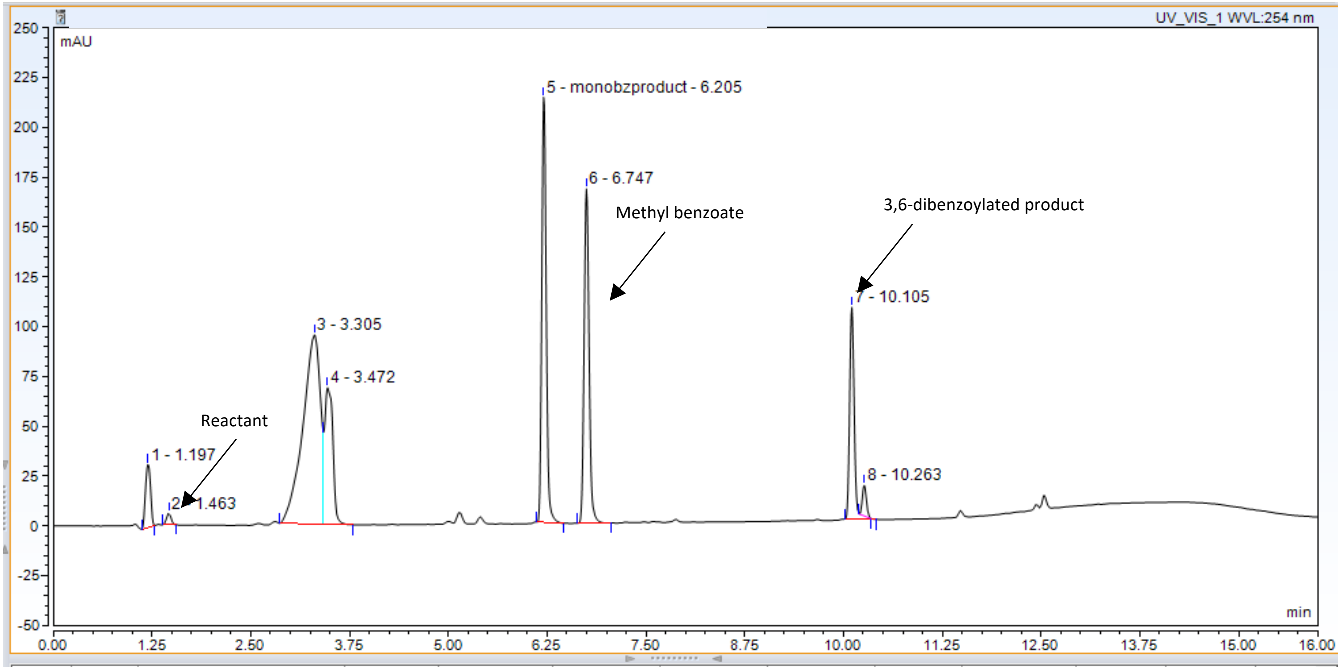

	A	B	C	D	E	F	G	H	I	J	K	L
1	Peak	Peak Name	Ret.Time	Amount	Rel.Area	Area	Height	Type	Width (50%)	Asym.	Resol.	Plate
2	No.		min		%	mAU*min	mAU		min	EP	EP	EP
4	1		1.197	n.a.	3.41	2.3872	31.71	BMB	0.074	1.04	2.28	14
5	2		1.463	n.a.	0.56	0.3898	5.86	BMB*	0.063	1.00	6.80	29
6	3		3.305	n.a.	35.16	24.5841	95.03	BM	0.256	n.a.	n.a.	9
7	4		3.472	n.a.	11.72	8.1935	68.60	MB	n.a.	n.a.	n.a.	i
8	5	monobzproduct	6.205	n.a.	20.02	13.9973	213.50	BMB	0.060	1.22	5.04	592
9	6		6.747	n.a.	17.51	12.2427	167.96	BMB	0.067	1.14	30.91	564
10	7		10.105	n.a.	10.35	7.2403	106.42	BMB	0.061	1.11	n.a.	1499
11	8		10.263	n.a.	1.27	0.8869	15.37	Rd	n.a.	n.a.	n.a.	i
12	Maximum			0.0000	35.16	24.5841	213.50		0.256	1.22	30.91	1499
13	Minimum			0.0000	0.56	0.3898	5.86		0.060	1.00	2.28	9
14	Sum			0.0000	100.00	69.9217	704.45					

Base descriptor sources

Table S2. Detailed overview of origin of base descriptors.

If not otherwise noted is data collected from: R. Stenutz, Tables for Organic Chemistry, http://www.stenutz.eu/chem/, accessed July 2022.

Compound	Cas	Molecular weight (g/mol)	Density (g/mL)	Molar volume (mL/mol)	Refractive index/nD	Melting point (°C)	Boiling point (°C)	LogP	Dipole moment(D)	Topological polar Surface Area (Å)	pKaH
Pyridine	110-86-1	79.1	0.983	80.5	1.51	-42	115	0.64	2.37	12.9	5.17
2-methylpyridine	109-06-8	93.13	0.94	99.1	1.501	-70	129	1.11	1.91	12.9	5.94
2,6-lutidine	108-48-5	107.16	0.921	116.3	1.497	-7	142	1.68	1.68	12.9	6.77
2,4-lutidine	108-47-4	107.16	0.931	115.2	1.501	-60	157	1.6	2.3	12.9	6.72
2,4,6-collidine	108-75-8	121.18	0.91	133.2	1.498	-43	172	1.88	2.04	12.9	7.48
DBU	6674-22-2	152.24	1.018	149.5	1.522	-70	81.5	1.38	3.41	15.6	11.9
Aniline	62-53-3	93.13	1.018	91.5	1.583	-6	184	0.94	1.51	26	4.58
Triethylamin	121-44-8	101.19	0.728	139.1	1.401	-115	90	1.45	0.87	3.2	10.78
Dipea	7087-68-5	129.25	0.755	171.2	1.457	-46	127	2.68	1.18	3.2	10.75

Pubchem, https://pubchem.ncbi.nlm.nih.gov/, accessed july 2022.

pKa Data Compiled by R. Williams.

Chemical book, https://www.chemicalbook.com/ChemicalProductProperty_EN_CB6368038.htm, accessed July 2022

Pitt Quantum Repository, http://pqr.pitt.edu/mol/JGFZNNIVVJXRND-UHFFFAOYSA-N, accessed July 2022

van der Plas, Henk C., and Alan R. Katritzky. *Advances in Heterocyclic Chemistry*. Elsevier, 1999.

Srivastava, R., *Journal of Molecular Catalysis A: Chemical* 264.1-2 (2007): 146-152.

Gimenez, D., et al., *Organic & Biomolecular Chemistry* 15.19 (2017): 4081-4085.

Dependency Plots

CI1 – Mono-gluco

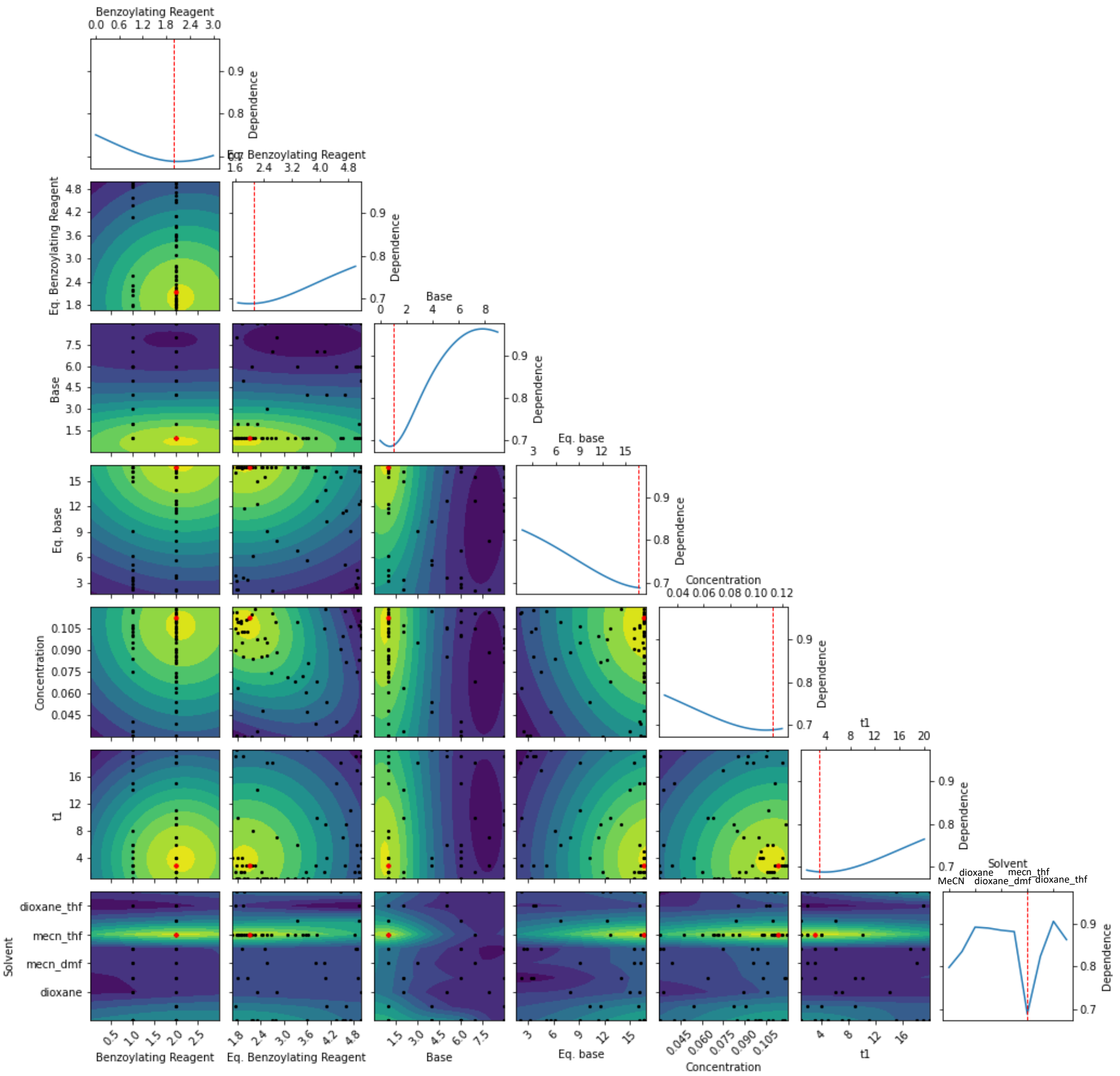

Figure S9a. Dependency plot of the estimated objective function. Black dots correspond to experiments carried out. Red dots and red dotted lines indicate the optimal reaction conditions found.

Figure S9a shows a dependency plot, which shows how the different parameters influence the yield when all other parameters take the value they have in the best set of conditions. In this way, it is possible to get insight into what the optimizer thinks the objective (the true function) looks like. In the contour plots, the yellow corresponds to minima (high yield), and the dark blue to low yields. The red dot in the contour plots and the dotted red line in the 2D plots corresponds to the observed optimal reaction conditions (experiment 63). It is seen that the influence of the concentration, equivalents benzoylating reagent and t1 are very similar. Equivalents base seems to be more important than the other continuous parameters. For the solvents, MeCN/THF seems to be the best, with pure MeCN the second best. Many of the optimal conditions' reaction parameters are either at or near the ends of their defined ranges.

CL2 – Mono-gluco

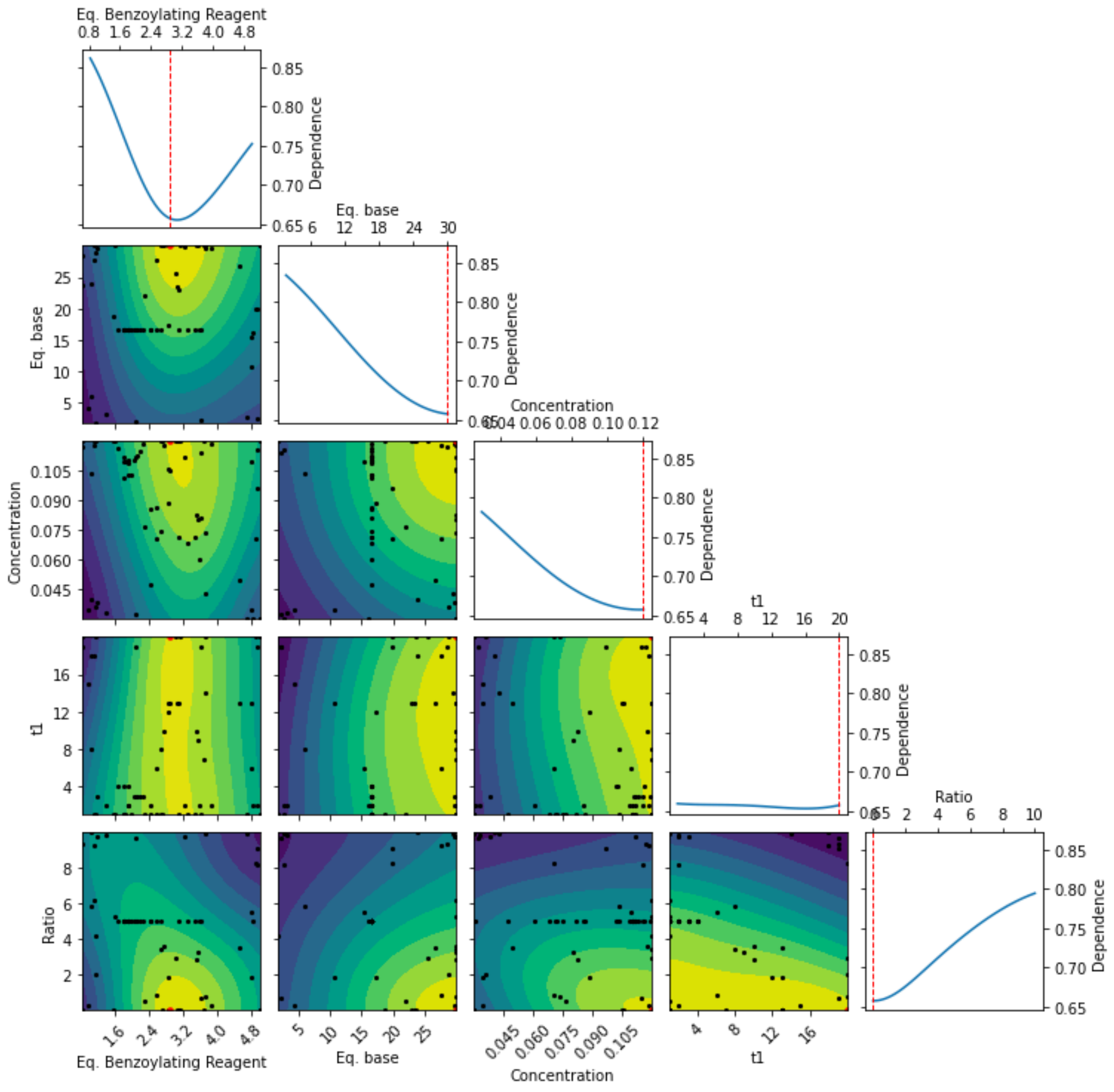

Figure S9b. Dependency plot of the estimated objective function. Black dots correspond to experiments carried out. Red dots and red dotted lines indicate the optimal reaction conditions found.

CL3 - Digluco

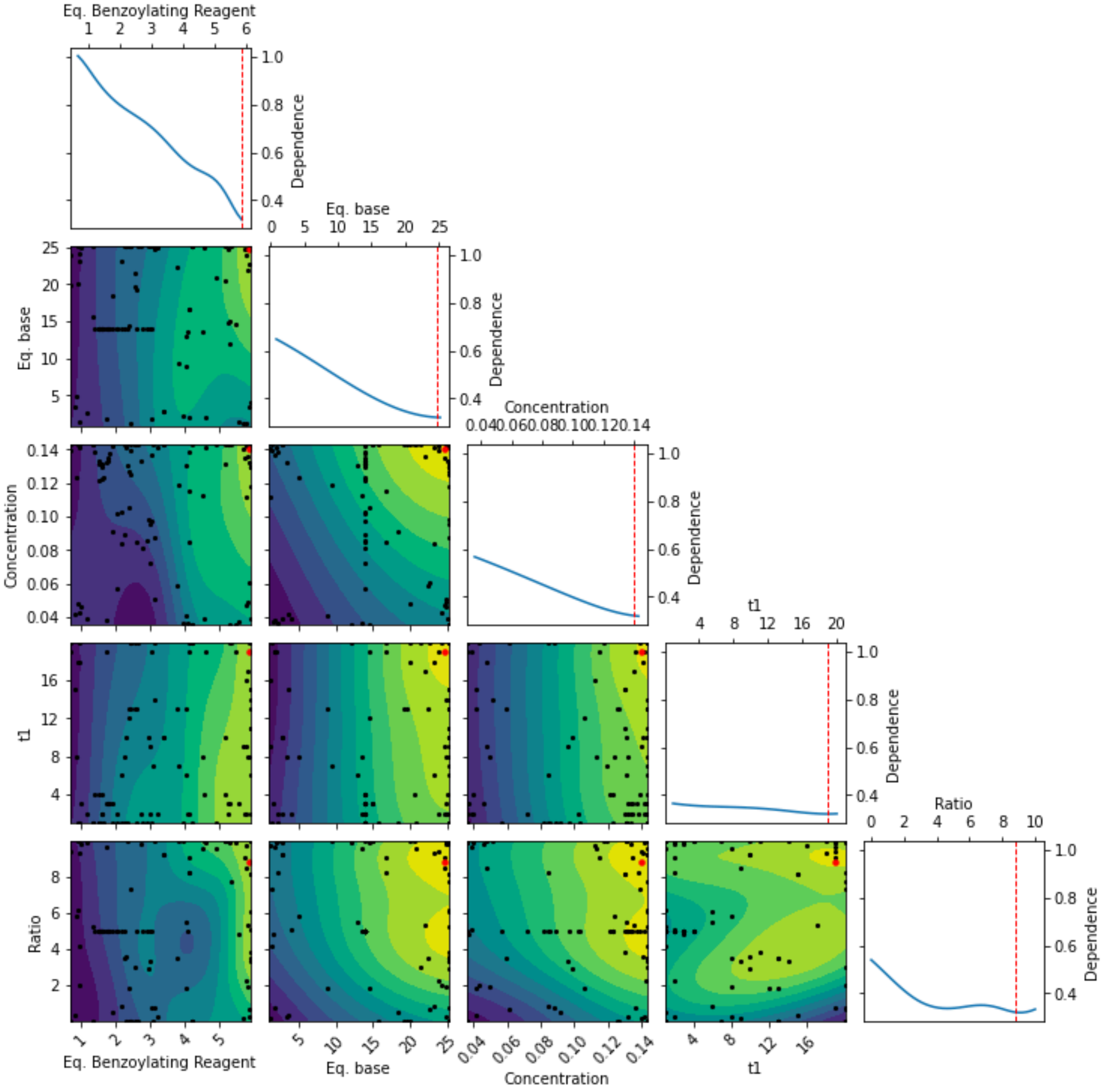

Figure S9c. Dependency plot of the estimated objective function. Black dots correspond to experiments carried out. Red dots and red dotted lines indicate the optimal reaction conditions found.

Cl4 – Mono-manno

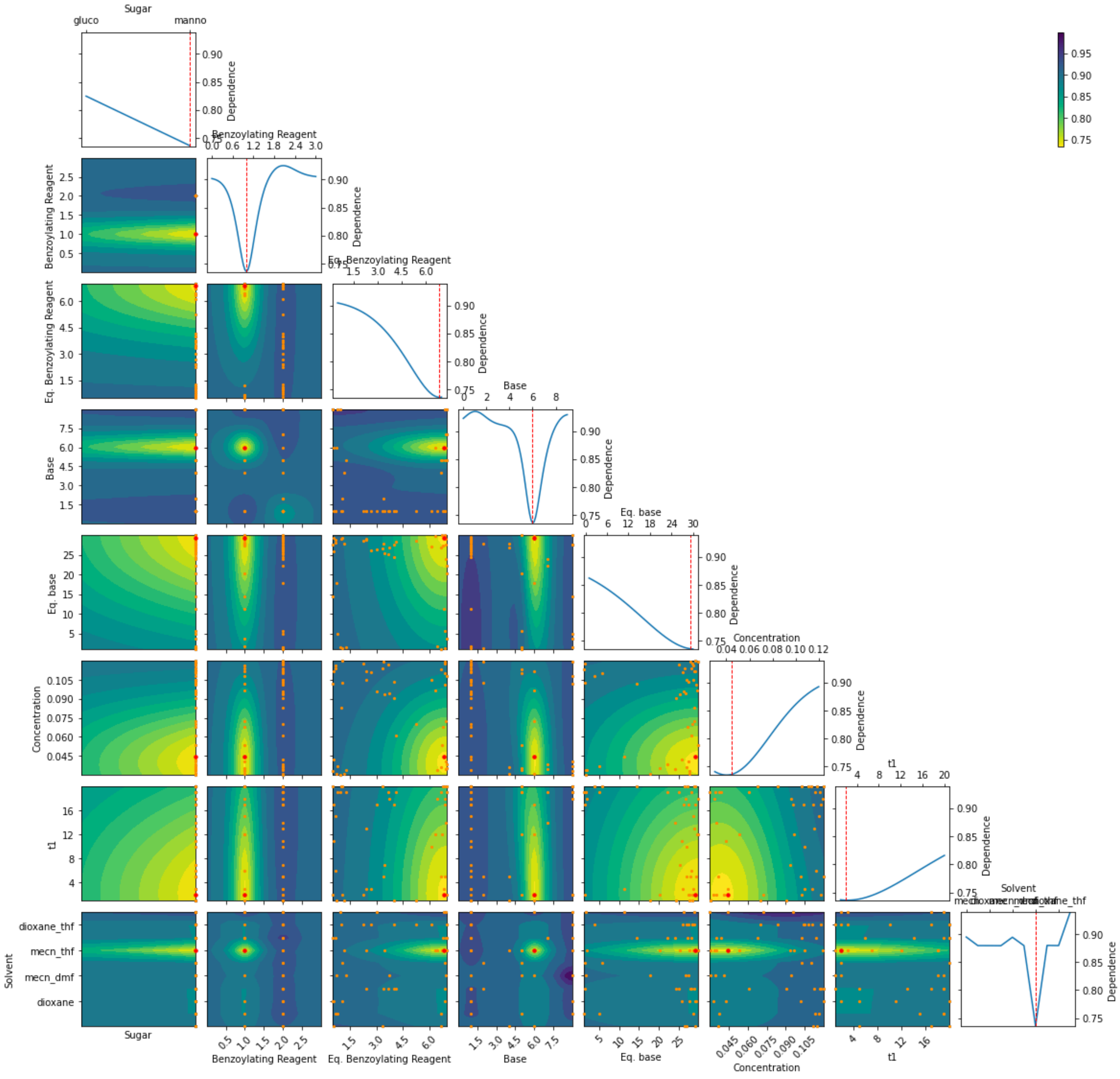

Figure S9d. Dependency plot of the estimated objective function. Black dots correspond to experiments carried out. Red dots and red dotted lines indicate the optimal reaction conditions found.

CI5 – Di-manno

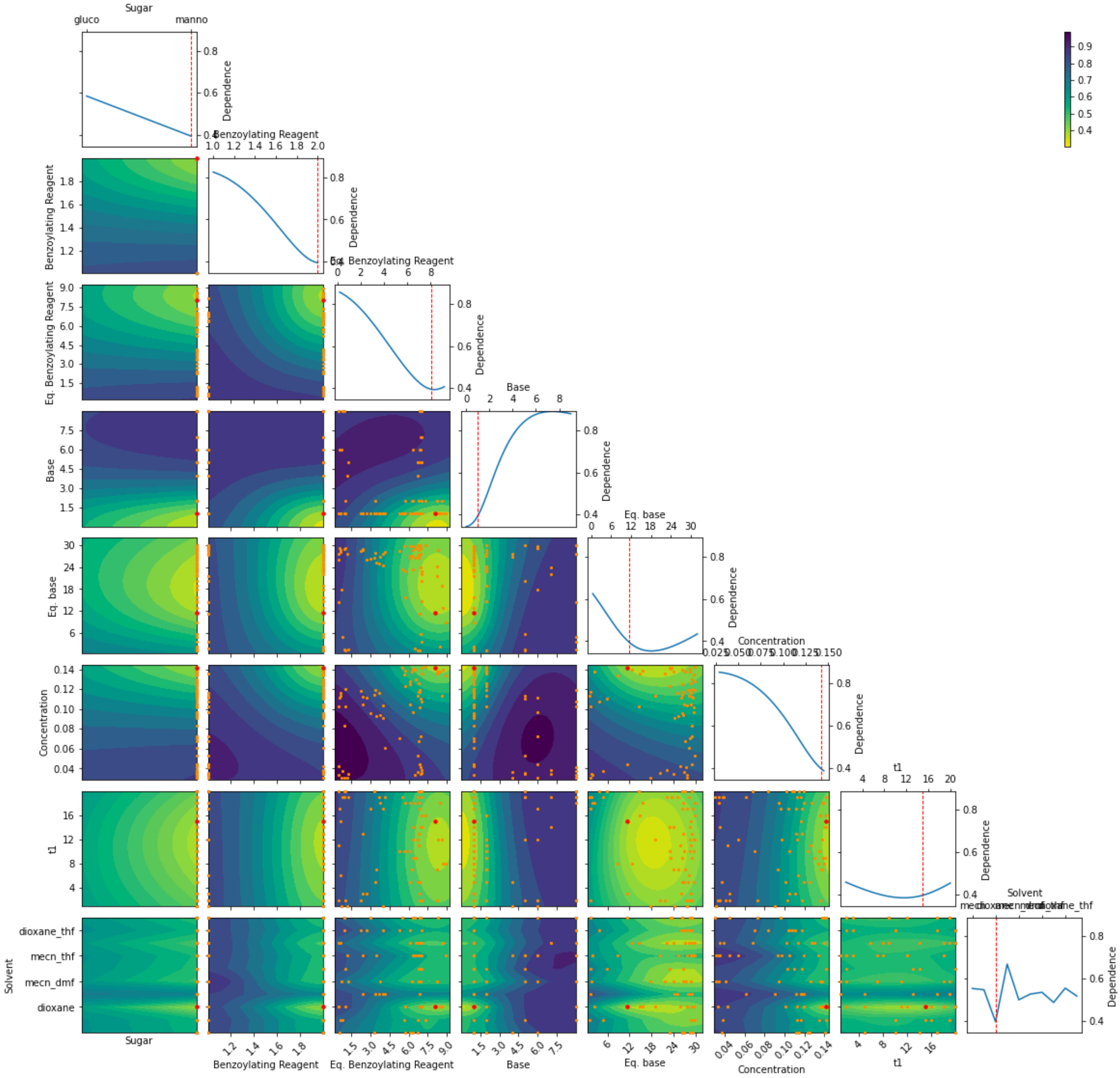

Figure S9e. Dependency plot of the estimated objective function. Black dots correspond to experiments carried out. Red dots and red dotted lines indicate the optimal reaction conditions found.

CL6 – Di-galacto

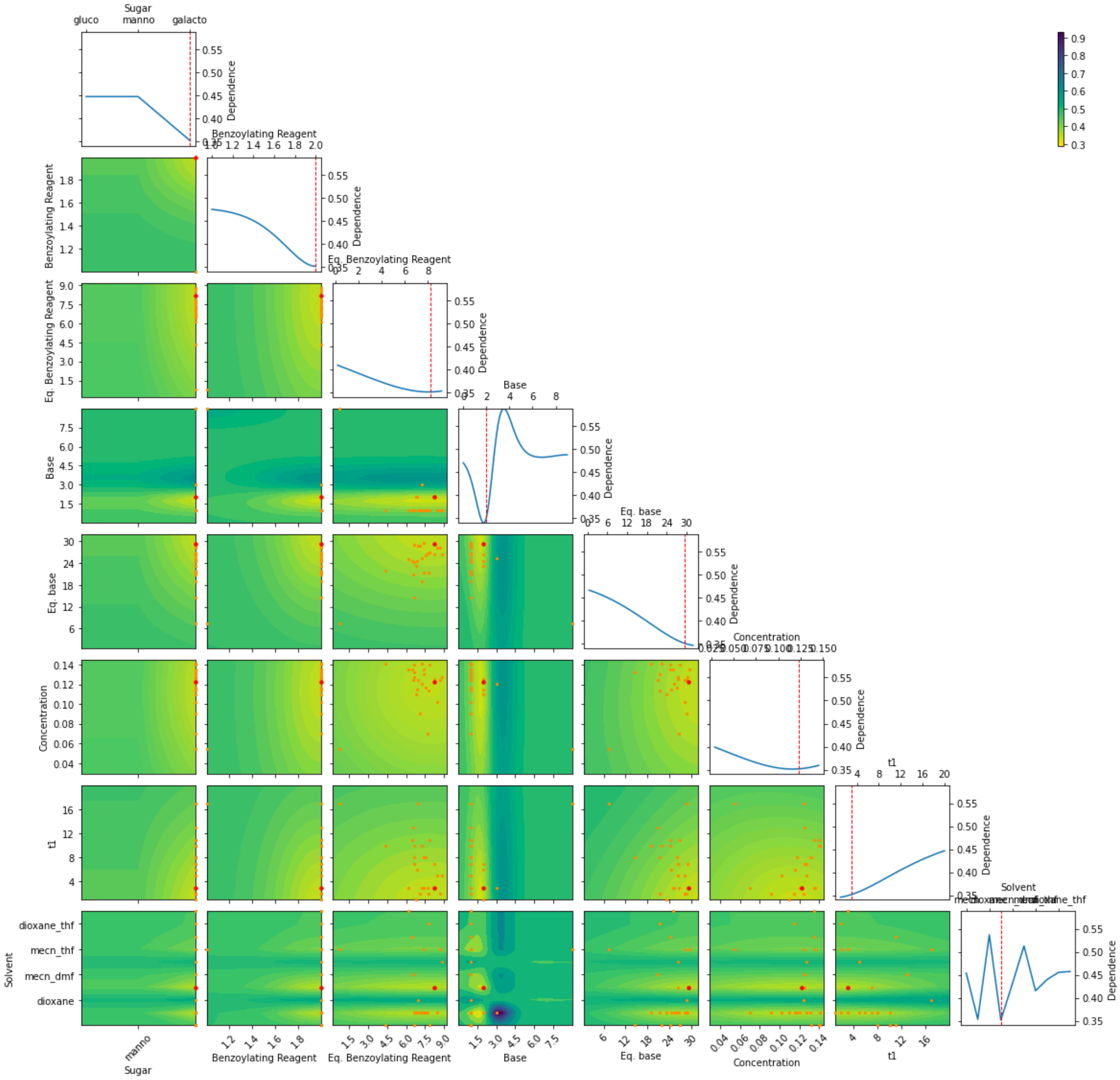

Figure S9f. Dependency plot of the estimated objective function. Black dots correspond to experiments carried out. Red dots and red dotted lines indicate the optimal reaction conditions found.

CL7 – Mono-galacto

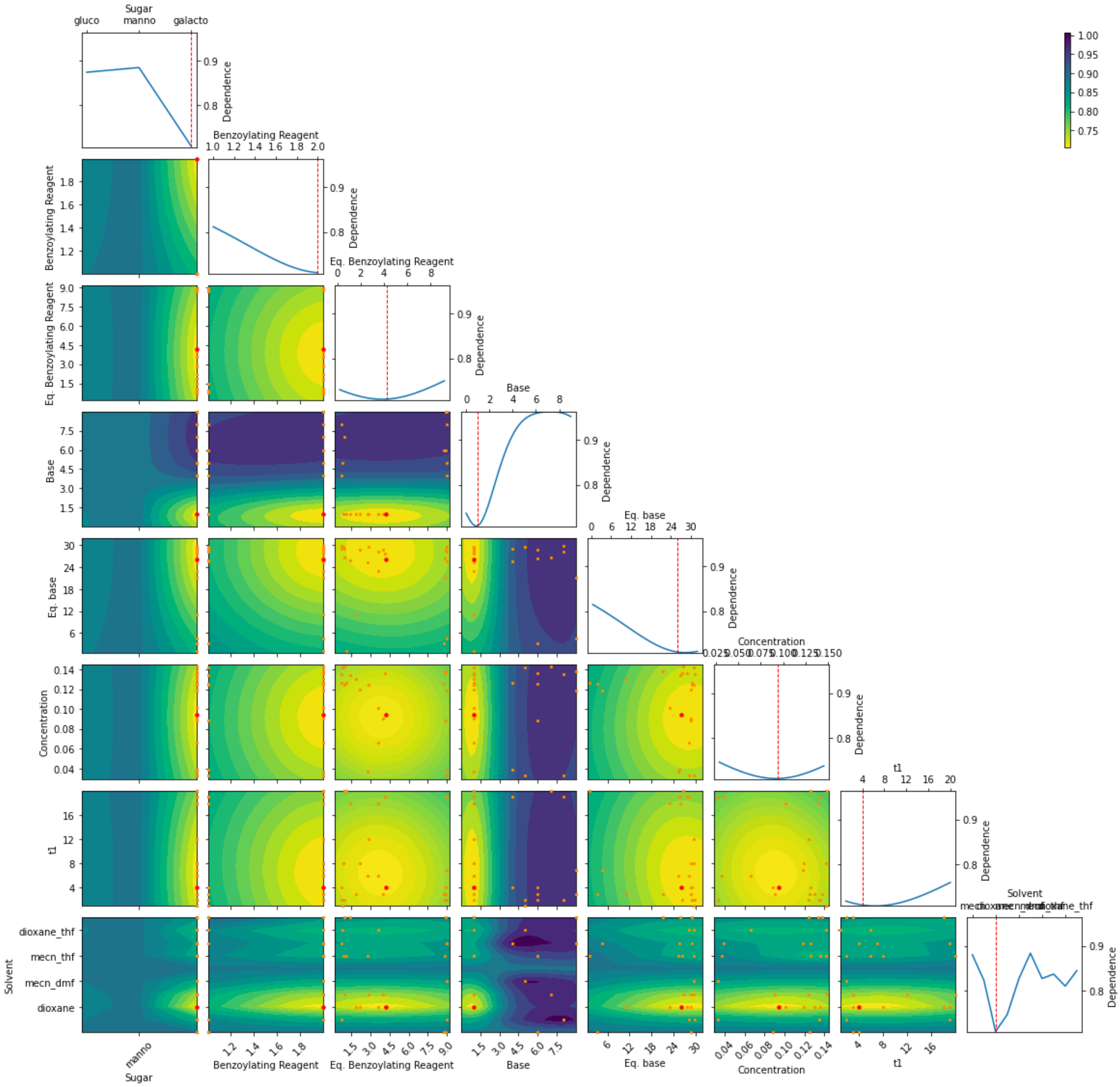

Figure S9g. Dependency plot of the estimated objective function. Black dots correspond to experiments carried out. Red dots and red dotted lines indicate the optimal reaction conditions found.

Closed-Loop 2

Reaction space

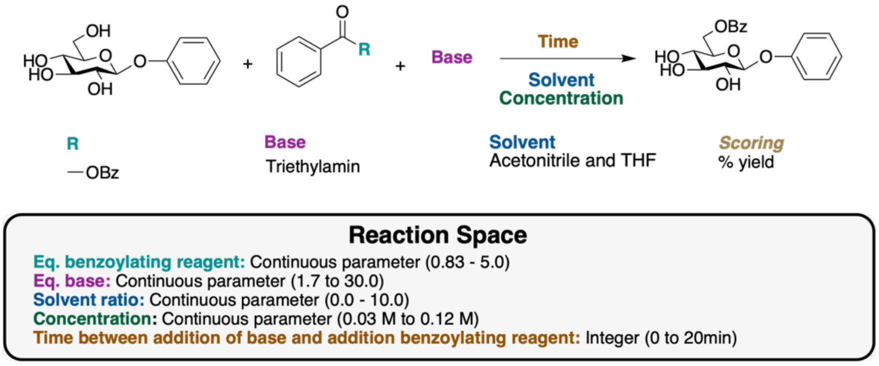

Figure S10. Reaction space for Closed-loop 2 (CL2)

Results

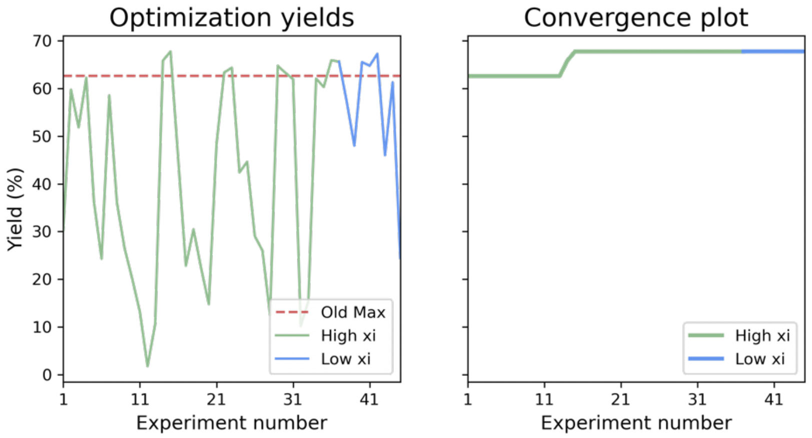

Figure S11. Optimization of monobenzoylation of a phenyl glucoside. High xi = 1.0, Low xi = 0.05, xi changeover = 36, initial data points from Cl2 = 24 (not shown)

All experiments from Cl2, which were within the new reaction space, were given to the optimizer as initial data points. The dotted red line shows the maximum yield from Cl2. It is seen that some experiments surpass the optimal yield observed in the previous Closed-loop, though only small improvements are observed. The convergence plot shows that convergence is reached after 15 experiments.

Pie charts of compound distribution

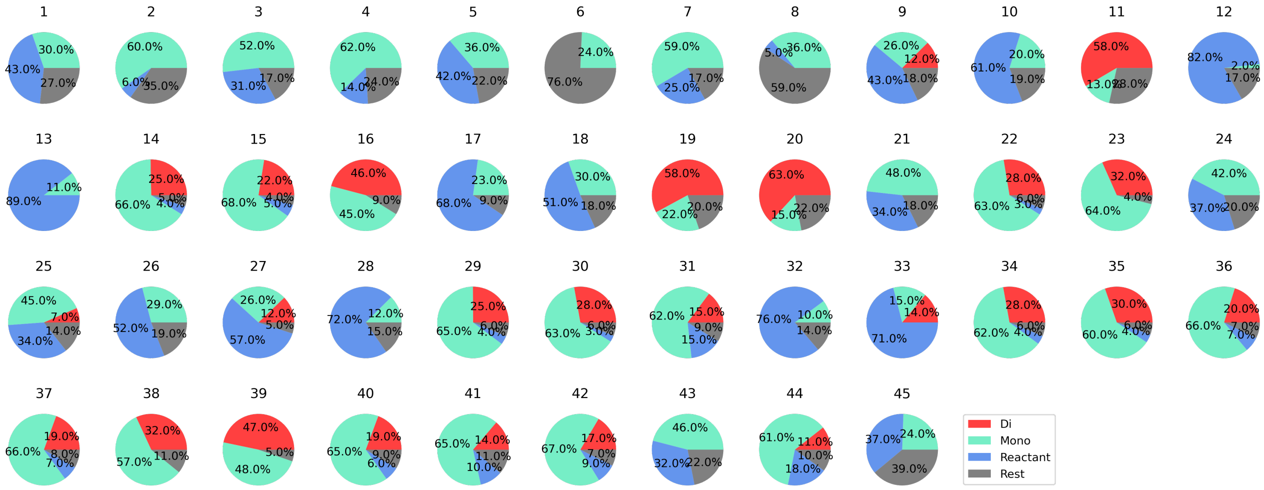

Figure S12. Pie charts showing the distribution of reactant, monobenzoylated and dibenzoylated glucosides in quenched reaction mixtures from Cl2.

Theory

In the following, a brief introduction to Bayesian optimization is provided. The focus will be on the different elements of the algorithm, but without going into detail with the mathematics as this is thoroughly described elsewhere.[3] The descriptions in this section are based on Francesco Archetti and Antonio Candelieri's *Bayesian optimization and Data Science*.[3]

Bayesian optimization

Bayesian optimization is based on Bayes theorem, which was proposed by Thomas Bayes in 1764.[4] Later the idea behind Bayes theorem was evolved into Bayesian optimization by Krige,[5] Kushner,[6] and Močkus.[7] Bayesian optimization is used to solve problems of the form:

$$\mathbf{x}^* = \arg\min_{\mathbf{x}\in X} f(\mathbf{x}) \tag{1}$$

Where $\mathbf{x}^*$ is the set of conditions within the space X, that minimizes the function $f(\mathbf{x})$. For Bayesian optimization to be a suitable optimization strategy three key criteria have to be fulfilled:[8]

1. $f(\mathbf{x})$ is a black box function, that is, $f(\mathbf{x})$ is not known, nor its gradients.
2. $f(\mathbf{x})$ is expensive to evaluate.
3. Evaluations of $f(\mathbf{x})$ are noisy.

If we want to apply Bayesian optimization to chemical reactions, we have to argue that a chemical reaction fulfills these criteria. This can be easily done by looking at a toy example. Let us say we want to maximize the yield of the reaction A + B $\rightarrow$ C by varying the reaction conditions $\mathbf{x}$ (e.g. concentration, solvent, the equivalent of B). Since we want to maximize the yield of C (minimize 100 - yield%), the reaction optimization is by definition a minimization problem. Since we cannot write a closed-form mathematical expression for the yield ($f(\mathbf{x})$) as a function of the reaction conditions, $f(\mathbf{x})$ is indeed a black box function, which fulfills criterion 1. If we want to evaluate $f(\mathrm{x})$, i.e. determine the yield obtained from a specific set of reaction conditions, we have to carry out the experiment, which is time-consuming, and hence expensive, fulfilling criterion 2. The last criterion states that evaluations of $f(\mathrm{x})$ are noisy, which chemical reaction optimization also conforms to, as duplicating an experiment rarely gives the exact same yield. To sum up, it seems that chemical reaction optimization is suitable for Bayesian optimization.

Bayesian optimization is in fact not very different from the human interpretation of the world. We base our beliefs about most things on prior observations. However, new observations might lead us to change, or update, our beliefs. The model that describes the algorithm's beliefs about the task is called the surrogate model. The surrogate models also contain information about how sure the algorithm is about its belief at a given point and using the surrogate model it is possible to come up with a qualified guess of where the minimum is.

In the following three key components of Bayesian optimization algorithms are discussed; Base estimator, Kernel, and Acquisition function.

Base Estimator: The base estimator is a probabilistic surrogate model. It is this model that incorporates prior knowledge and models the objective function and the uncertainties. The most widely used base estimator is a Gaussian process (GP) but different tree regression models are also commonly used.[3,9] To a given set of data points, there are an infinite number of functions that will fit the data. Gaussian processes work by assigning a probability to each of these functions, providing a mean function with uncertainties. A Gaussian process is defined by a mean vector μ and a covariance matrix. The covariance matrix describes how each data point varies according to the other data points and specifies the distribution of the functions. The covariance matrix is calculated using a pre-defined covariance function, which is also termed the kernel.

Kernel: The kernel encodes assumptions about the function that is approximated. Many different kernels exist e.g. the squared exponential kernel, the Rational Quadratic Kernel, and Matérn kernels. The Matérn kernels have two hyperparameters, ν and l, with l describing the length of the correlations, i.e. determining if the data is only strongly correlated to the nearby data points or also to data points far away. ν controls the smoothness of the function.

Acquisition function: The acquisition function is a function that guides the search for an optimum using the probabilistic surrogate model generated by the base estimator, by describing the potential gain. Again, many different acquisition functions are available including the probability of improvement (PI),[6] expected improvement (EI),[10] and lower-confident bound (LCB).[11] One of the major challenges for the acquisition function is to balance exploration and exploitation. An exploring acquisition function will query the points with high uncertainty and explore all parts of $f(\mathrm{x})$, thereby not utilizing the previous data points in an efficient way. An exploiting acquisition function will stay close to the data point that is the current optimum, hence it is likely to get stuck in a local optimum. The hyperparameter that controls the trade-off between exploration and exploitation is denoted ξ (xi). For instance, is LCB, in the case of minimization, given by:

$$LCB(X) = \mu(x) - \xi\sigma(x) \tag{2}$$

$\xi \geq 0$ and $\xi = 0$ corresponds to pure exploration. When an acquisition function is chosen, a method for minimizing the acquisition function also has to be determined. This is commonly done using either random sampling or a genetic algorithm. If the surrogate function is a Gaussian process, optimization algorithms, like the limited-memory Broyden-Fletcher-Goldfarb-Shanno algorithm (L-BFGS), can also be used for minimizing the acquisition function.

References

[1] B. J. Shields, J. Stevens, J. Li, M. Parasram, F. Damani, J. I. M. Alvarado, J. M. Janey, R. P. Adams, and A. G. Doyle, *Nature*, **2021**, *590*, 89–96.

[2] T. Lieser and R. Schweizer, *Justus Liebigs Ann. Chem.*, **1935**, *519*, 271–278.

[3] F. Archetti and A. Candelieri, *Bayesian optimization and data science;* Springer, 2019.

[4] T. Bayes, *Philos. Trans. R. Soc.*, **1763**, (53), 370–418.

[5] D. G. Krige, *J. South. Afr. Inst. Min. Metall.*, **1951**, *52*, 119–139.

[6] H. J. Kushner, *J. Basic Eng.*, **1964**, *86*, 97–106.

[7] J. Močkus, *On Bayesian methods for seeking the extremum. In Optimization Techniques IFIP Technical Conference;* Springer, 1975.

[8] G. Louppe and M. Kumar; Bayesian optimization with skopt, **2016;** https://scikit-optimize.github.io/stable/auto_examples/bayesian-optimization.html (*Accessed July 2022).*

[9] T. K. Ho; Random decision forests; In *Proceedings of 3rd international conference on document analysis and recognition*, Vol. 1, pages 278–282. IEEE, 1995.

[10] J. Mockus, V. Tiesis, and A. Zilinskas, *J. Glob. Optim.*, **1978**, *2*, 2.

[11] P. Auer, *J. Mach. Learn. Res.*, **2002**, *3*, 397–422.

Author Contributions

NVF: Project design, data acquisition, data analysis, synthesis, writing of scripts, and writing of the original draft.

CMP: Supervision and funding acquisition.

RHT: Supervision.
